# Supplementary material for: Structural analysis of Red1 as a conserved scaffold of the RNA-targeting MTREC/PAXT complex
Source: Nat Commun. 2022 Aug 24;13:4969. doi: 10.1038/s41467-022-32542-3 (PMC9402713; doi:10.1038/s41467-022-32542-3)
Supplement: Supplementary file 1 — Supplementary Information [file 41467_2022_32542_MOESM1_ESM.pdf]

SUPPLEMENTARY INFORMATION

Structural analysis of Red1 as a conserved scaffold of the RNA-targeting MTREC/PAXT complex

Anne-Emmanuelle Foucher[1,6], Leila Touat-Todeschini[2,6], Ariadna B. Juarez-Martinez[1,6], Auriane Rakitch[2], Hamida Laroussi[1], Claire Karczewski[2], Samira Acajjaoui[3], Montserrat Soler-López[3], Stephen Cusack[4], Cameron D. Mackereth[5*], André Verdel[2*] and Jan Kadlec[1*]

Contains 15 Supplementary Figures, 4 Supplementary Tables and Supplementary references.

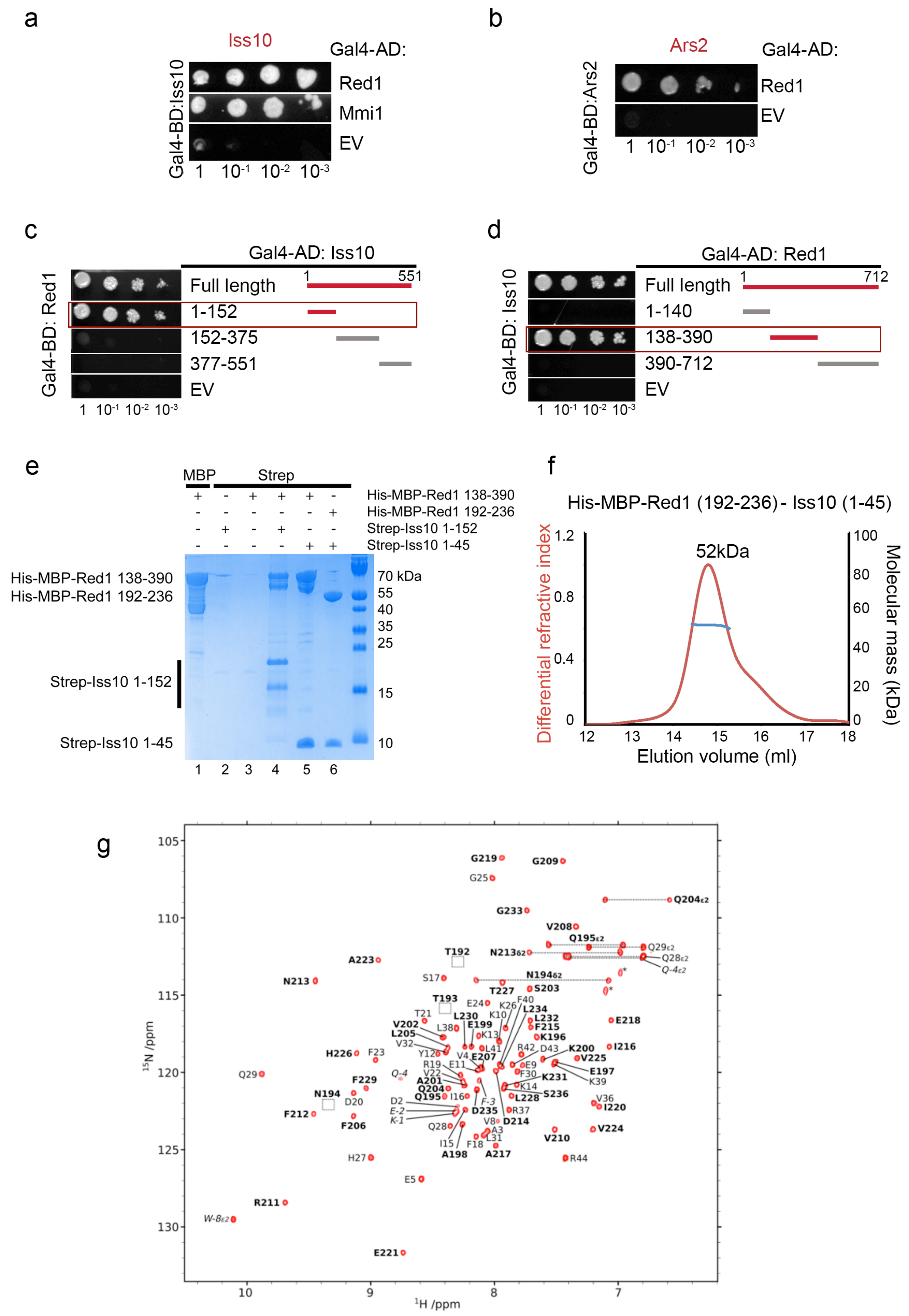

Supplementary Figure 1

a Y2H assays with Red1 as a prey showing that Iss10 interacts with Red1. Iss10 interacts also with Mmi1. Growth was assayed on medium lacking histidine and with 20mM 3AT. **b** Y2H assays with Red1 as a prey showing that Ars2 interacts with Red1. Growth was assayed on medium lacking histidine and with 20mM 3AT**.** **c** Mapping of the Iss10 interacting regions with Red1 by Y2H assays, revealing the Iss10 N-terminus to be sufficient for the interaction. Growth was assayed on medium lacking histidine and with 50mM 3AT. EV – empty vector**.** **d** Mapping of the Red1 interacting regions with Iss10 by Y2H assays, showing that the Red1 middle segment mediates the interaction. **e** SDS-PAGE analysis of Strep-tag pull-down experiments with Red1 and Iss10. The indicated combinations

of His-MBP-Red1 and Strep-tagged Iss10 constructs were co-expressed and then purified on Strep-Tactin columns. His-MBP-Red1$^{138\text{-}390}$ purified alone on Amylose resin is shown as a control in lane 1. Source data are provided as a Source Data file. **f** Molecular mass determination of the His-MBP-Red1$^{192\text{-}236}$ - Strep-Iss10$^{1\text{-}45}$ complex by MALLS. The measured molecular mass of 52 kDa corresponds to a 1:1 stoichiometry. Calculated Mw of the complex is 56 kDa (49.4 + 6.7 kDa). The complex was injected at 2 mg/ml. **g** Annotated ^{15}N-HSQC spectrum of the Iss10-Red1 heterodimer. The spectrum was collected at 298 K at a field strength of 800 MHz on a sample of 150 µM [^{15}N]Iss10-Red1 in 20 mM Tris (pH 7.5), 150 mM NaCl, 5 mM beta-mercaptoethanol and 10% (v/v) D_2O. Amide ^{1}H,^{15}N crosspeaks are annotated by residue type and number. Residues in normal type are from Iss10, and in bold type for Red1. Annotations in italics are for residues in the expression tag, and unassigned side chain amides are indicated with an asterisk. Squares indicate the location of amide crosspeaks with low intensity due to line-broadening.

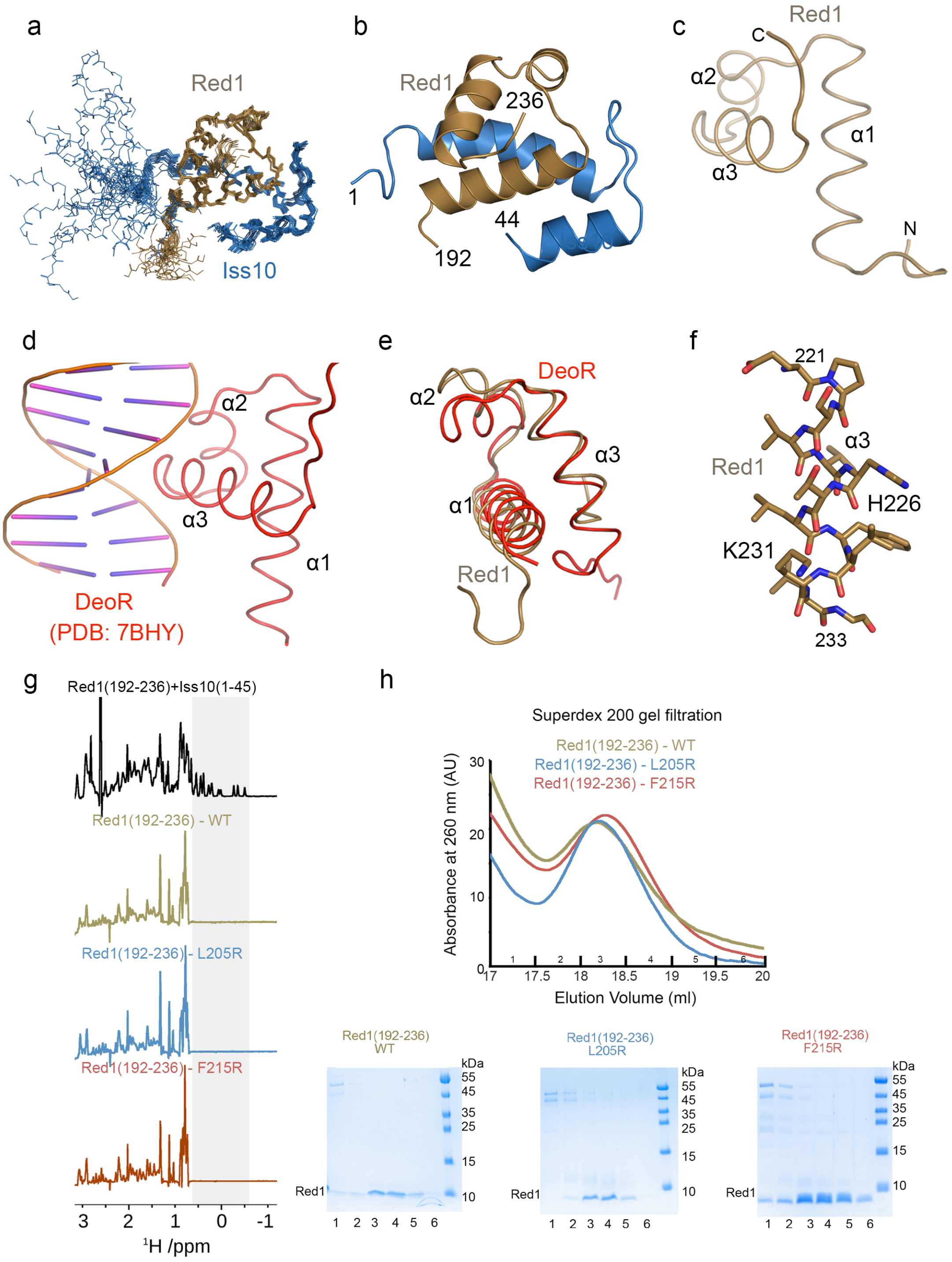

Supplementary Figure 2

a Superposition of 20 energy-minimized conformers representing the 3D NMR structure of the complex between Red1$^{192-236}$ and Iss10$^{1-45}$. Red1 is shown in brown and Iss10 in blue. **b** Cartoon representation of the structured part of the complex including Red1 residues 192-236 and Iss10 residues 1-44. **c** The HTH domain of Red1. **d** The HTH domain of *B. subtilis* DeoR in complex with dsDNA (PDB code-7HBY - http://doi.org/10.2210/pdb7BHY/pdb)[1]. The structure was superimposed on Red1 shown in **c** with r.m.s.d. of 2.3 Å for 74 Cα atoms. **e** Superposition of HTH motifs of Red1 and DeoR. **f** Detail view of side chains of the Red1 helix α3. **g** Methyl region from the 1D NMR spectra of the Red1-Iss10 complex (black, top spectrum), as well as the isolated Red1$^{192-236}$ wild-type, Red1$^{192-236}$ L205R, and Red1$^{192-236}$ F215R constructs. Methyl peaks with chemical shifts less than 0.7

ppm indicate the formation of a stable hydrophobic core within the Red1-Iss10 complex. The wild-type and mutant Red1 constructs all lack evidence of a stable fold, with no methyl peaks having a chemical shift less than 0.7 ppm. In addition, all three isolated Red1 constructs are soluble and show no sign of aggregation or higher order oligomerization as evident by the narrow peak linewidths. Protein concentrations are 20-40 μM in 20mM Tris pH 8, 100mM NaCl and 5mM b-mercaptoethanol, with NMR measured at 298 K at a field strength of 700 MHz. **h** Superdex 200 gel filtration elution profiles of WT, L205R and F215R Red1$^{192-236}$ and SDS-PAGE analysis of fractions 1-6 for each protein. Red1$^{192-236}$ does not contain aromatic residues absorbing well at 280nm, but its elution could be followed by a weak absorbance at 260nm. Source data are provided as a Source Data file.

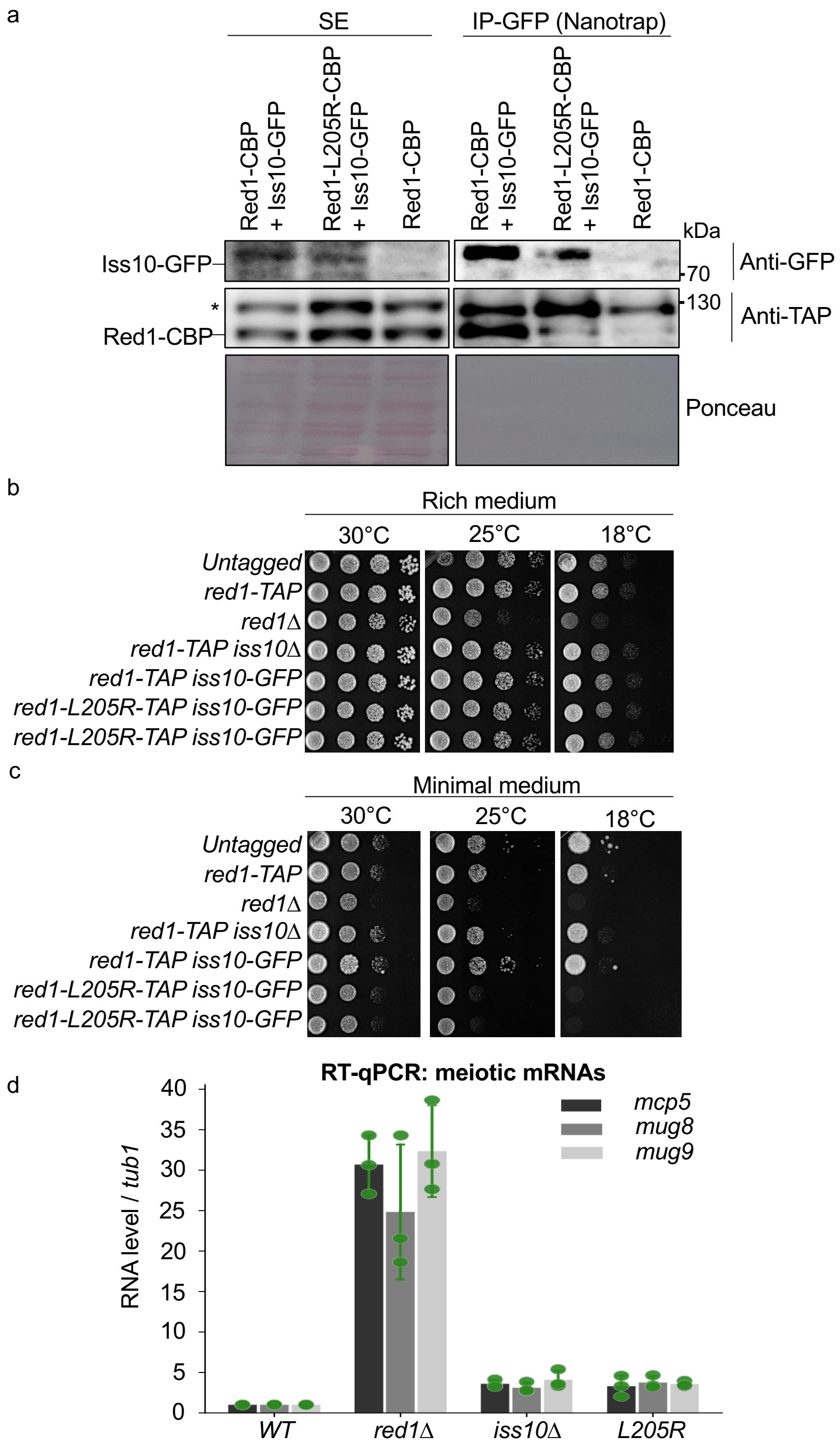

Supplementary Figure 3

a Co-immunoprecipitation experiments of Red1-CBP and Iss10-GFP analysed by western blot. Soluble cell (SE) extracts were prepared from exponentially growing cells in minimal liquid medium. Asterisk indicates a background signal generated from the remaining Red1-TAP proteins after TEV cleavage (see Method for further details). **b,c** Growth assays comparing the proliferation of *red1-L205R-TAP* mutant cells to the parental cells, expressing the endogenous and untagged Red1 (*Untagged*), to cells expressing the wild-type Red1 fused to the TAP tag (*red1-TAP* or *WT*), to cells

deleted for *red1* coding sequence (*red1*Δ), and to cells deleted for *iss10* coding sequence (*iss10*Δ). Cells were grown on rich **b** or minimal **c** medium, at temperatures of 30, 25 or 18ºC. **d** Quantitative RT-PCR showing the level of *mcp5*, *mug8* and *mug9* mRNAs normalized to *tub1* mRNA in *WT* and *red1-L205R* mutant cells. Total RNAs were purified from exponentially growing cells in liquid rich medium. Error bars represent standard deviation from three independent biological replicates. Source data are provided as a Source Data file.

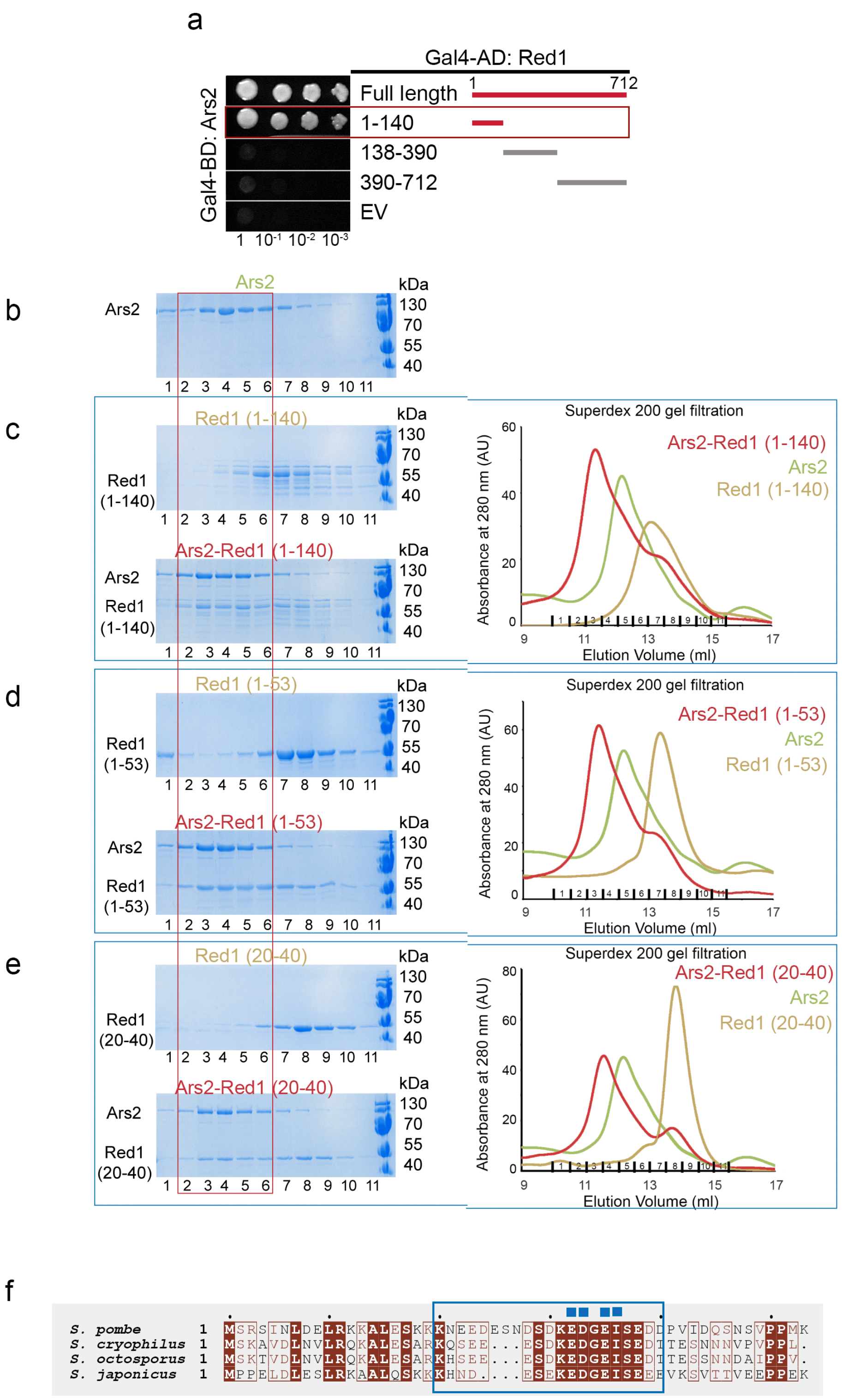

Supplementary Figure 4

a Y2H assays revealed that the N-terminal part of Red1 mediates the interaction with Ars2. Growth was assayed on medium lacking histidine and with 20mM 3AT. EV – empty vector. **b** SDS-PAGE analysis of fractions 1-11 of the Superdex 200 gel filtration elution profile of FL His-MBP-Ars2. **c** Left panel - SDS-PAGE analysis of fractions 1-11 of the S200 gel filtration elution profiles of His-MBP-Red1$^{1\text{-}140}$ and its complex with FL His-MBP-Ars2. Right panel: overlay of Superdex 200 gel filtration elution profiles of FL His-MBP-Ars2, His-MBP-Red1$^{1\text{-}140}$ and their complex. The proteins were expressed individually as MBP fusions and purified on amylose resin. Both proteins were first purified on Superdex 200. **d** Superdex 200 gel filtration profiles and SDS-PAGE analysis of eluted

fractions as described above, of His-MBP-Red1$^{1\text{-}53}$ and its complex with FL His-MBP-Ars2. **e** Superdex 200 gel filtration profiles and SDS-PAGE analysis of eluted fractions as described above, of His-MBP-Red1$^{20\text{-}40}$ and its complex with FL His-MBP-Ars2. **f** Sequence alignment of the Red1 proteins from different *Schizosaccharomyces* species. Only the sequence of residues 1-53 covering the N-terminal fragment involved in the interaction with Ars2 is shown. The minimal binding segment identified above is highlighted with a blue rectangle. Identical residues are in brown boxes. Blue squares indicate residues whose mutation impacts the interaction as shown in Fig. 5d-f. Source data are provided as a Source Data file.

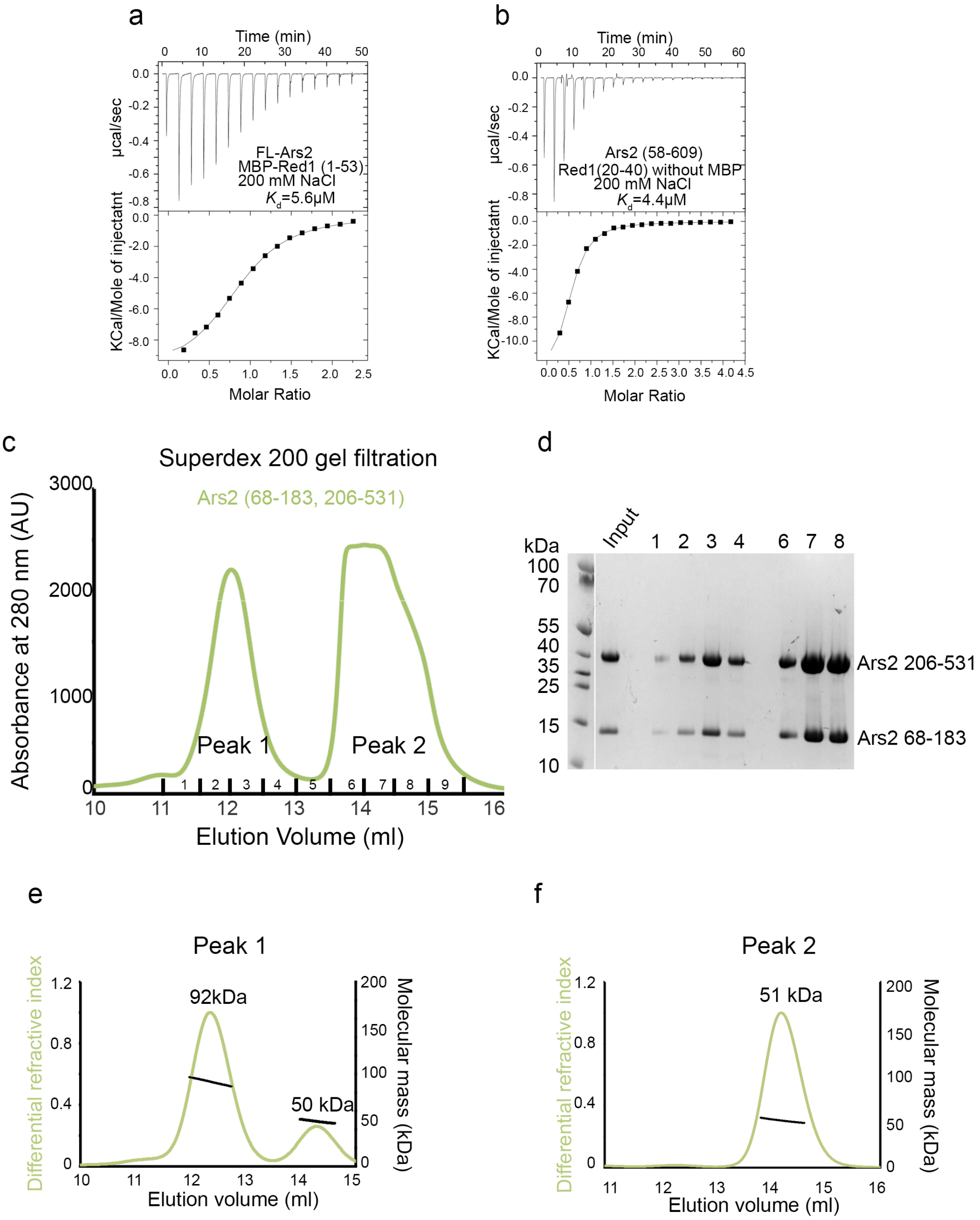

Supplementary Figure 5

a ITC measurement of the interaction affinity between FL Ars2 and His-MBP-Red1$^{1-53}$ in presence of 200mM NaCl. **b** ITC measurement of the interaction affinity between Ars2$^{58-609}$ and Red1$^{20-40}$ peptide in presence of 200mM NaCl. **c** Superdex 200 gel filtration elution profile of Ars2$^{68-183,206-531}$ revealing two elution peaks. **d** SDS-PAGE analysis of fractions 1-11 of the Superdex 200 gel filtration elution profile of Ars2$^{68-183,206-531}$ shown in **c**. Source data are provided as a Source Data file. **e** Molecular mass determination of the *S. pombe* Ars2$^{68-183,206-531}$ originating from the first elution peak shown in **c** by MALLS. The measured molecular masses of 92 and 50 kDa correspond to its dimer and monomer, respectively. The protein was injected at 38 mg/ml. **f** MALLS measurement of the molecular mass of the *S. pombe* Ars2$^{68-183,206-531}$ eluting in the second peak shown in The measured molecular mass of 51 kDa corresponds to a monomer. The protein was injected at 4 mg/ml.

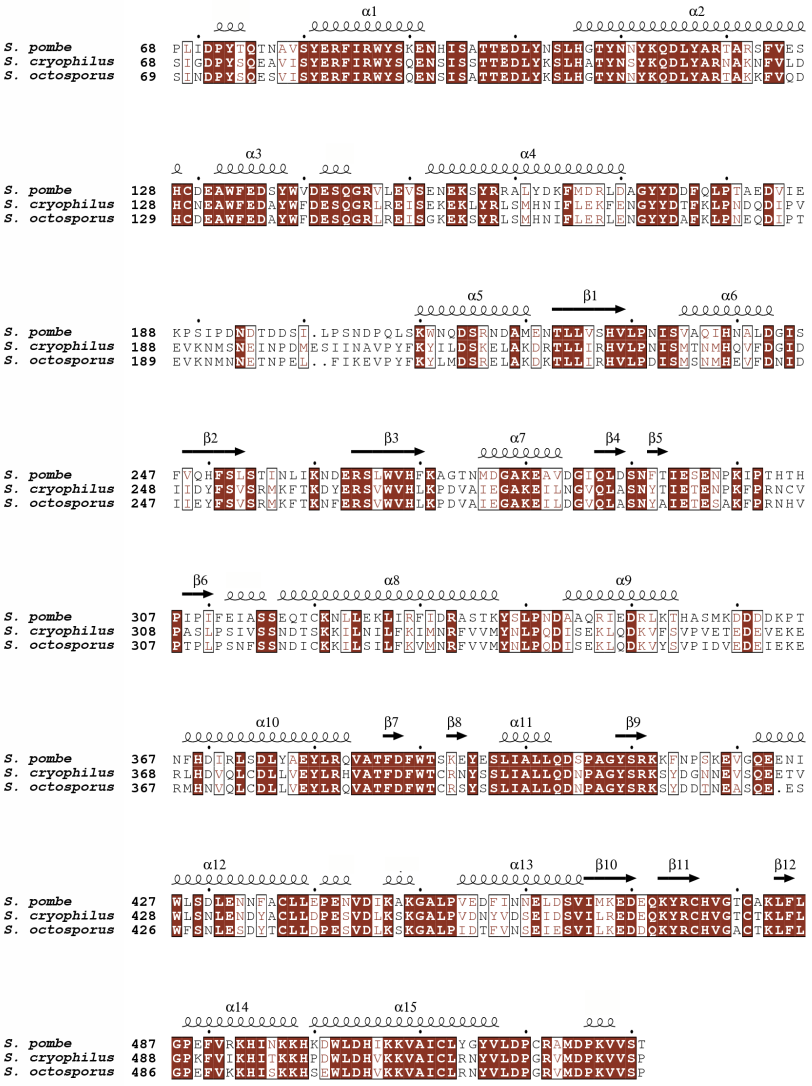

Supplementary Figure 6

Sequence alignment of Ars2 proteins from *Schizosaccharomyces* species. Identical residues are in brown boxes.

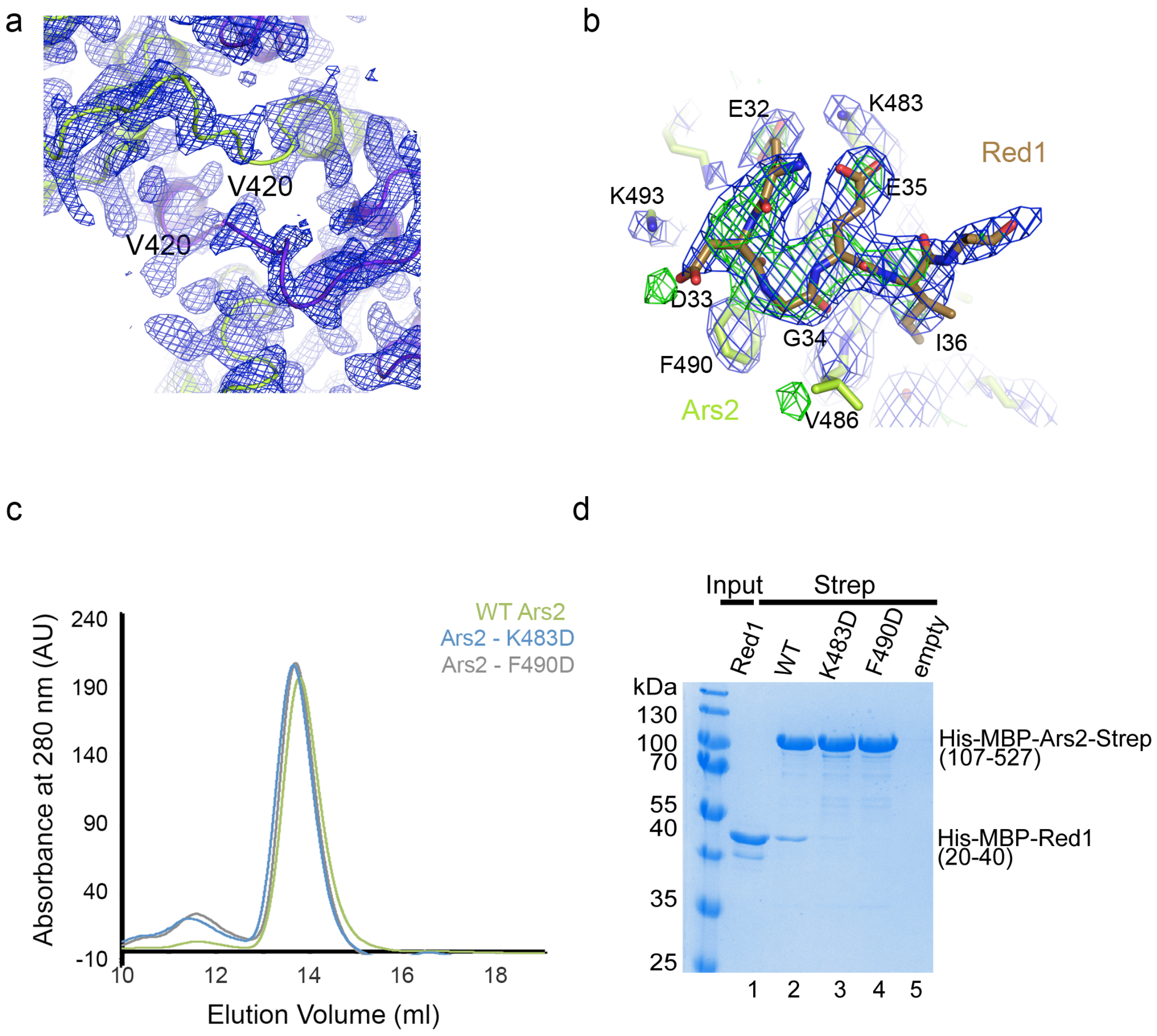

Supplementary Figure 7

a A 2*F*o-*F*c electron density map covering the two hinge regions around V420 mediating the swap of the C-terminal domain between the two Ars2 protomers contoured at 1.0 σ. **b** 2*F*o-*F*c (blue) and omit *F*o-*F*c (green) electron density maps covering the Red1 residues interacting with Ars2 contoured at 1.0 and 2.5 σ, respectively. **c** Superdex 200 gel filtration elution profiles of WT and mutant ARS2 constructs used in panel **d**. **d** Strep-tag pull-down experiments with His-MBP-Red1$^{20\text{-}40}$ and Strep-Ars2$^{107\text{-}527}$ mutants indicated above the lanes. Ars2 and Red1 proteins were first purified using Ni^{2+} resin (lane 1). Red1 with Ars2 variants were then co-purified on Strep-Tactin resin (lanes 2-4). Source data are provided as a Source Data file.

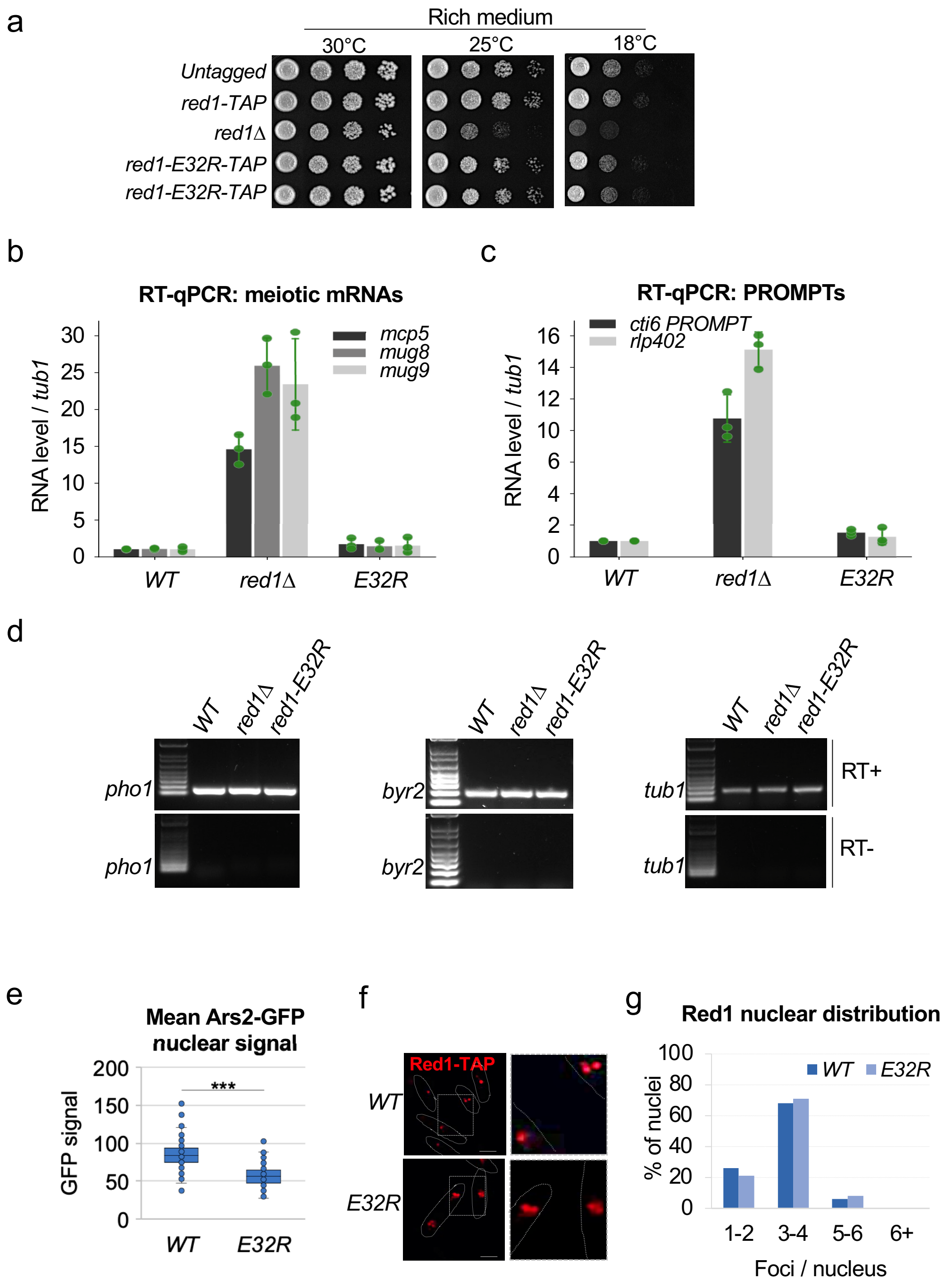

Supplementary Figure 8

a Growth assays comparing the growth of *red1-E32R-TAP* mutant cells to the parental cells, which express endogenous and untagged Red1 (*Untagged*), to cells expressing wild-type Red1 fused to the TAP tag (*red1-TAP* or *WT*), to cells deleted for *red1* coding sequence (*red1*Δ). Cells were grown on rich medium, at temperatures of 30, 25 or 18ºC. **b** Quantitative RT-PCR monitoring the level of meiotic DSR-containing mRNAs (*ssm4*, *spo5*, *rec8*) and **c** of PROMPTs-CUTs (*cti6* and *rlp402* PROMPTs) in *WT* and *red1-L205R* mutants. Total RNAs were purified from exponentially growing cells in liquid minimal medium. Variation of RNA levels is expressed in fold - change relative to the

WT levels and normalized to *tub1* mRNA. Error bars represent standard deviation from three independent biological replicates. **d** Agarose gel pictures showing RT-PCR amplification products to monitor the level of *pho1* and *byr2* mRNAs in *WT*, *red1Δ* and *red1-E32R* mutant cells. **e** Boxplots showing the mean intensity of the nuclear Ars2-GFP signal in *WT* and *red1-E32R* mutant cells. The analyses were performed on a minimum of 100 nuclei from two independent biological replicates. Wild-type box plot has a minima of 37.3, maxima of 152.2 and a centre value of 84.1. *red1-E32R* boxplot has a minima of 29.9, maxima of 102.6 and a centre value of 56.3. Each boxplot is divided in four 25% quartiles. The mean difference was examined with a student t-Test. ***= a p-value of 3.915×10^{-50}, determined from the two-tailed test. **f** Images of immuno-fluorescence microscopy showing Red1-TAP localization in *WT* and *red1-E32R* cells. Scale bar = 5 µm. **g** Graph representing the foci number per nucleus of Red1-TAP in *WT* and *red1-E32R* cells. Count performed on 100 nuclei from two different biological isolates. Source data are provided as a Source Data file.

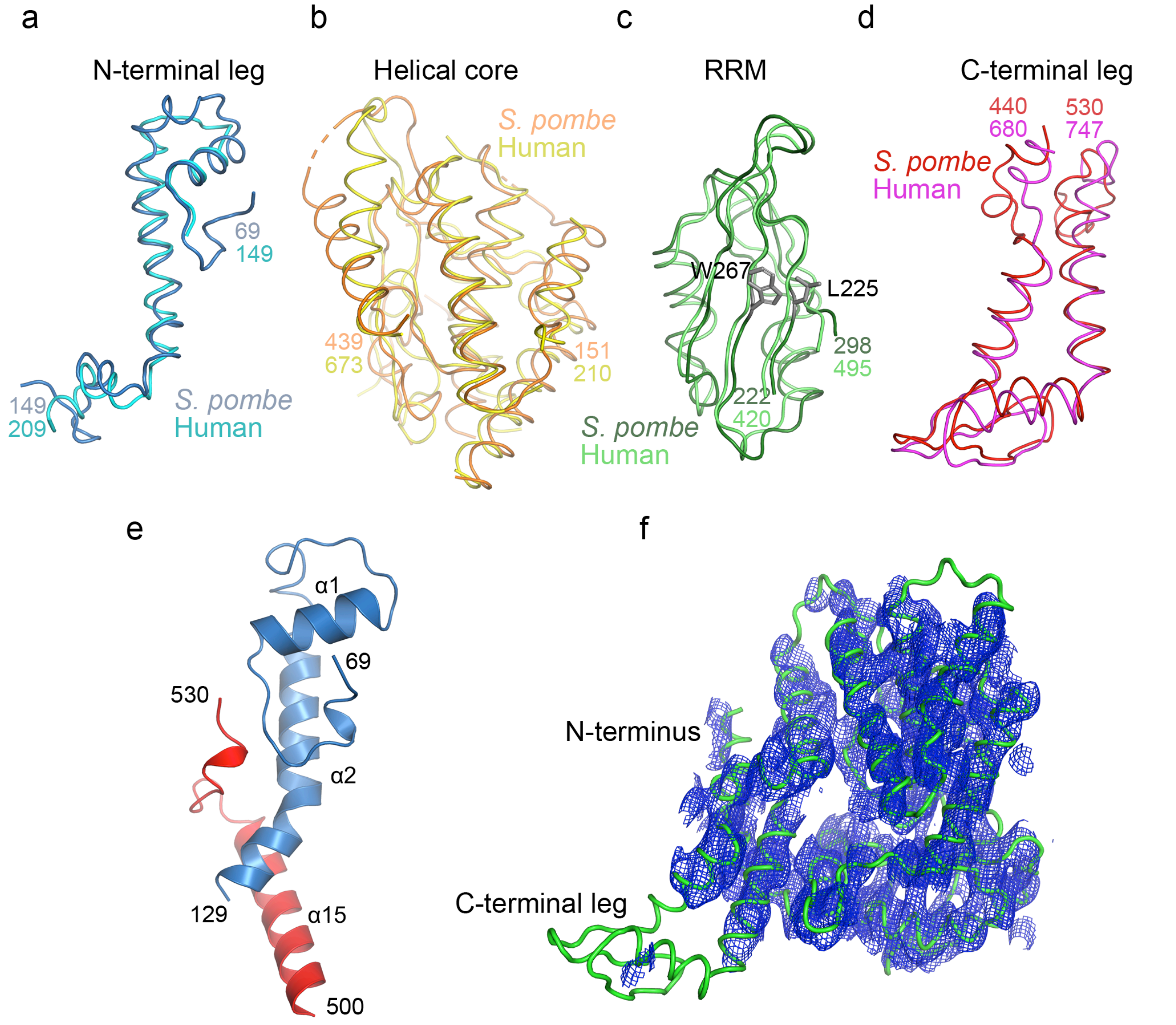

Supplementary Figure 9
a Comparison of *S. pombe* and human Ars2 N-terminal leg (r.ms.d.- 2.2 Å for 52 Cα atoms).
b Comparison of *S. pombe* and human Ars2 helical core (r.ms.d.- 2.8 Å for 153 Cα atoms).
c Comparison of *S. pombe* and human Ars2 RRM domain (r.ms.d.- 1.6 Å for 74 Cα atoms).
d Comparison of *S. pombe* and human Ars2 C-terminal leg (r.ms.d.- 4.2 Å for 60 Cα atoms). **e** The N-terminal segment (residues 69-80) packs against helices α1 and α2 and interact with C-terminal residues 527-530. **f** A 5 Å resolution 2*F*o-*F*c electron density map covering a monomer of Ars2$^{107-527}$. The N-terminal helix and Zn-finger domain of the C-terminal leg are disordered. The structure was determined by molecular replacement using the Alphafold *S. pombe* Ars2 model (AF-094326). The model bias was reduced using the Prime-and-switch phasing in Resolve[2].

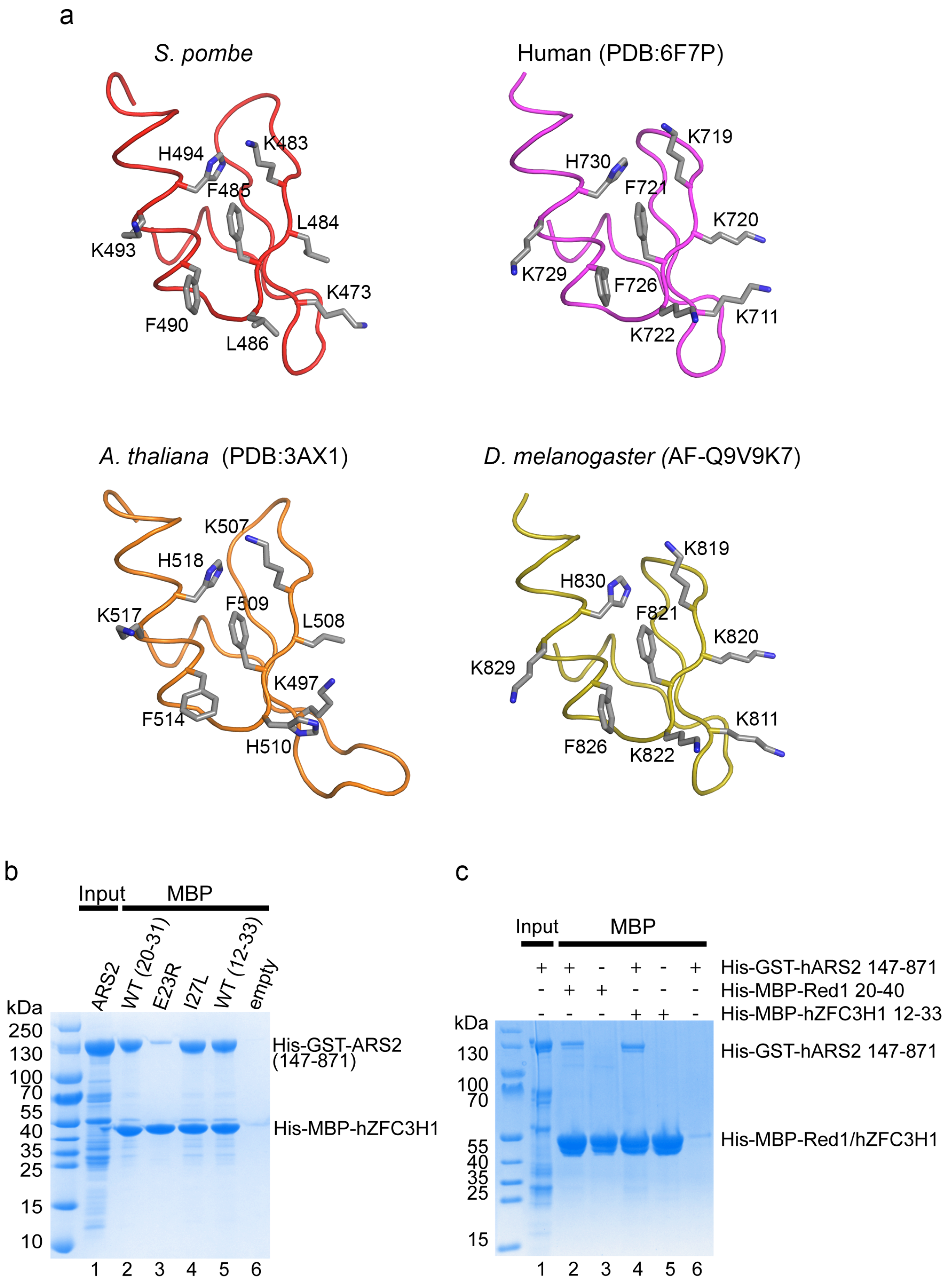

Supplementary Figure 10

a Comparison of the conserved Red1-binding surface among *S. pombe* (this study), human (PDB code-6F7P - http://doi.org/10.2210/pdb6F7P/pdb) and *A. thaliana* (PDB code-3AX1-http://doi.org/10.2210/pdb3AX1/pdb) structures and the AplhaFold2 model of *D. melanogaster* ARS2 (AF-Q9V9K7). **b** MBP-pull down experiment with human His-GST-ARS2 and His-MBP-ZFC3H1. ZFC3H1$^{20\text{-}33}$ is sufficient for ARS2$^{147\text{-}871}$ binding (lane 2). E23R mutation abolished the interaction (lane 3) while I27L retains a WT level of binding (lane 4). **c** MBP-pull down experiment, showing that human His-GST-ARS2$^{147\text{-}871}$ can interact with both His-MBP-ZFC3H1$^{12\text{-}33}$ and His-MBP-Red1$^{20\text{-}40}$. Source data are provided as a Source Data file.

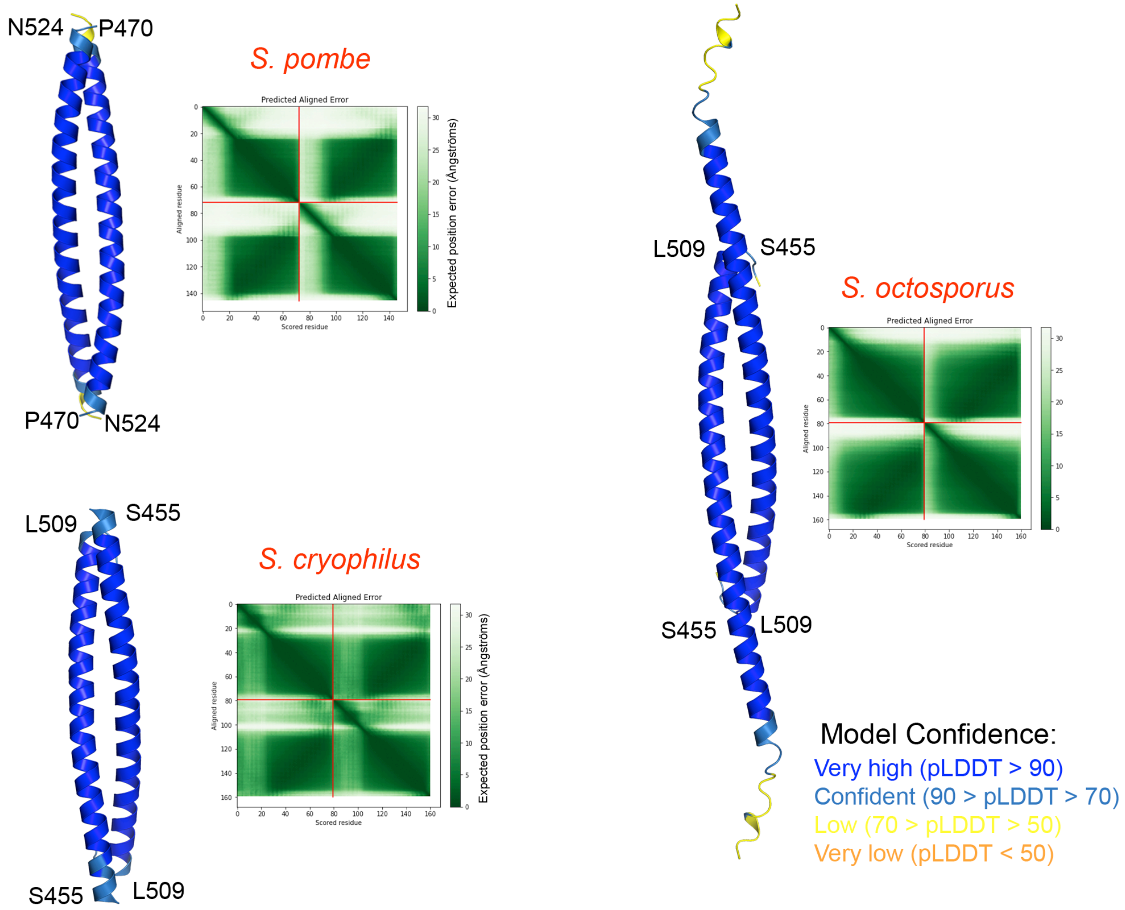

Supplementary Figure 11

AlphaFold2 models of the *S. pombe, S. octosporus and S. cryophilus* Red1 C terminus. Only the dimeric coiled-coil regions, corresponding to the alignment in Fig. 7c are shown. For *S. octosporus*, longer chains, predicted with high confidence are shown. The structures are colored according to the Aplhafold per-residue confidence score (pLDDT). Predicted aligned error plot is shown for each model.

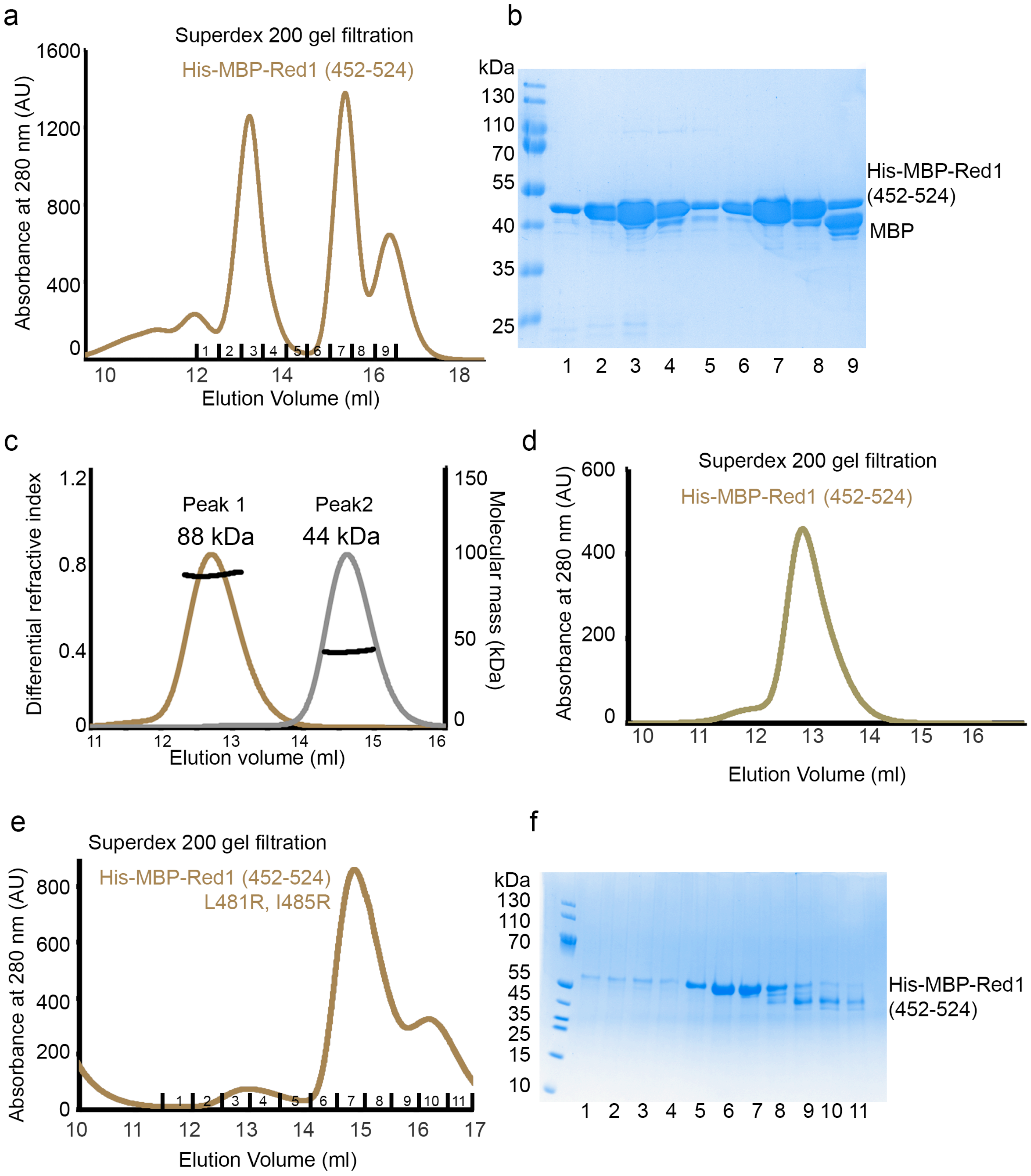

Supplementary Figure 12

a Superdex 200 gel filtration elution profile of His-MBP-Red1$^{452\text{-}524}$ revealing two elution peaks (third peak corresponds to MBP). **b** SDS-PAGE analysis of fractions 1-10 of the Superdex 200 gel filtration elution profile of His-MBP-Red1$^{452\text{-}524}$ shown in **a**. **c** Molecular mass determination of the His-MBP-Red$^{452\text{-}524}$ by MALLS. The measured molecular masses of 88 kDa and 44 kDa correspond to a dimer and monomer of Red1 (peak 1 and 2 of gel filtration shown in **a**). Calculated molecular mass of a monomer is 53 kDa. The two samples were injected at 9 and 5 mg/ml, respectively. **d** 2nd round Superdex 200 gel filtration of His-MBP-Red1$^{452\text{-}524}$ originating from the first elution peak shown in **a.** **e** Superdex 200 gel filtration profile of His-MBP-Red1$^{452\text{-}524}$ (L481R, I485R) mutant. The protein mostly elutes in a peak corresponding to the monomer. A large portion of the mutated protein also elutes in the void volume, indicating that monomeric His-MBP-Red1$^{452\text{-}524}$ becomes unstable. **f** SDS-PAGE-Red1$^{452\text{-}524}$ (L481R, I485R) shown in **e**. Source Data are provided as a Source Data file.

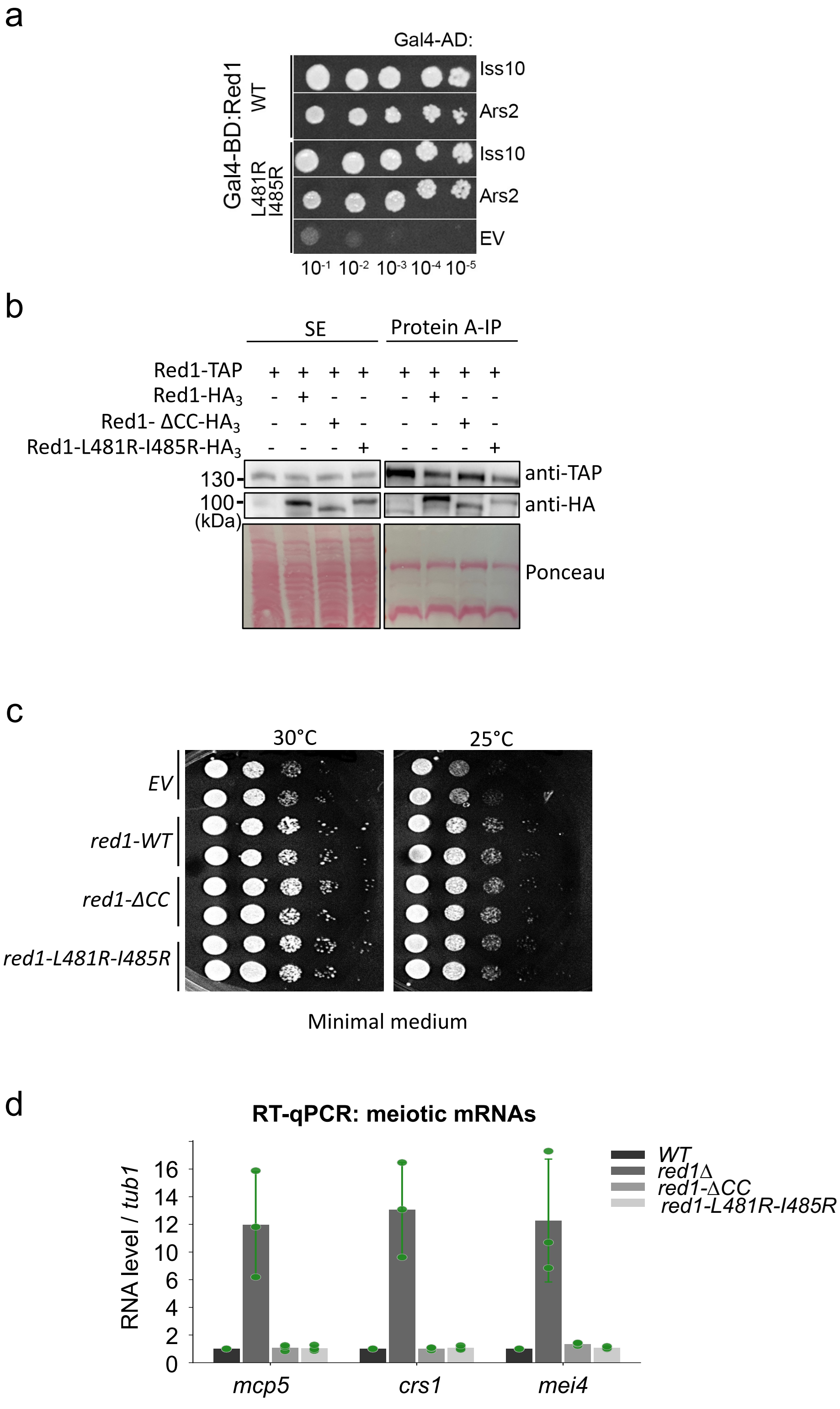

Supplementary Figure 13

a Y2H assay showing that the L481R, I485R double mutant in Red1 does not impact the interactions of FL Red1 with Iss10 and Ars2. Growth was assayed on medium lacking histidine and with 30mM 3AT. **b** Co-immunoprecipitation experiments of Red1-TAP and Red1-HA_3, Red1-ΔCC-HA_3 or Red1-L481R-I485R-HA_3, analysed by western blot. Soluble cell (SE) extracts were prepared from exponentially growing cells in minimal liquid medium. **c** Growth assays comparing the growth of *red1*Δ cells expressing from a plasmid wild-type Red1, Red1-ΔCC or Red1-L481R-I485R. Cells were

grown on minimal medium, at temperatures of 30 or 25ºC. **d** Quantitative RT-PCR monitoring the level of *mcp5*, *crs1* and *mei4* meiotic mRNAs in *red1*Δ cells expressing from a plasmid wild-type Red1, Red1-ΔCC or Red1-L481R-I485R mutants. Total RNAs were purified from exponentially growing cells in liquid minimal medium. Error bars represent standard deviation from three independent biological replicates. Source data are provided as a Source Data file.

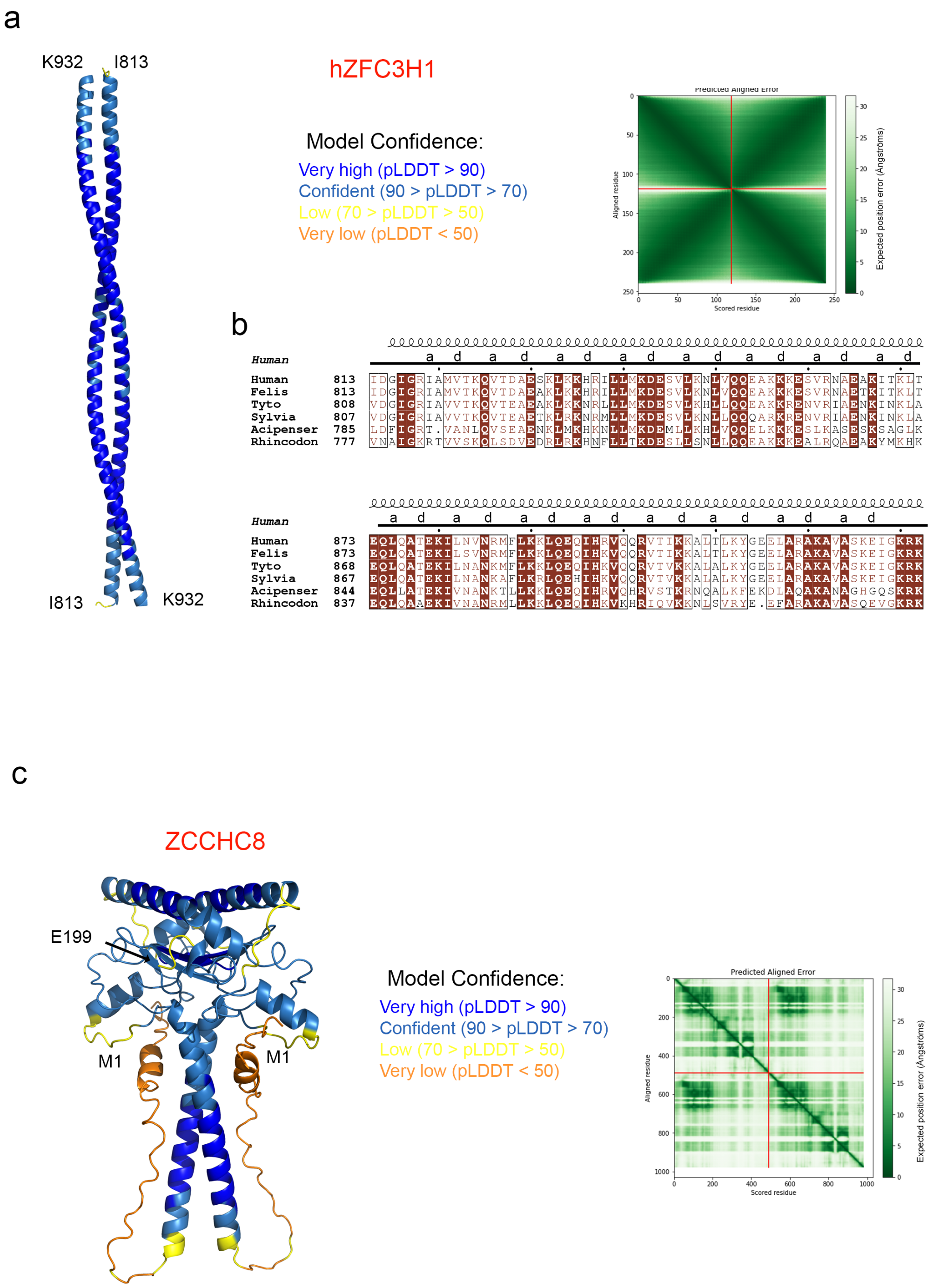

Supplementary Figure 14

a AlphaFold2 model of human ZFC3H1 dimer. Only the dimeric coiled-coil regions, corresponding to the alignment in **b** are shown. The structure is colored according to the AlphaFold2 pLDDT score. Predicted aligned error plot is shown. **b** Sequence alignment of ZFC3H1 proteins. Only the sequence of the coiled-coil region is shown. Identical residues are in brown boxes, and conserved residues are shown in brown. Heptad positions are indicated. **c** AlphaFold2 model of human ZCCHC8 dimer. Only residues 1-199 are shown, tht were predicted with confidence of mutual positions of the two protomers according to the predicted aligned error plot. The structure is colored according to the AlphaFold2 pLDDT score.

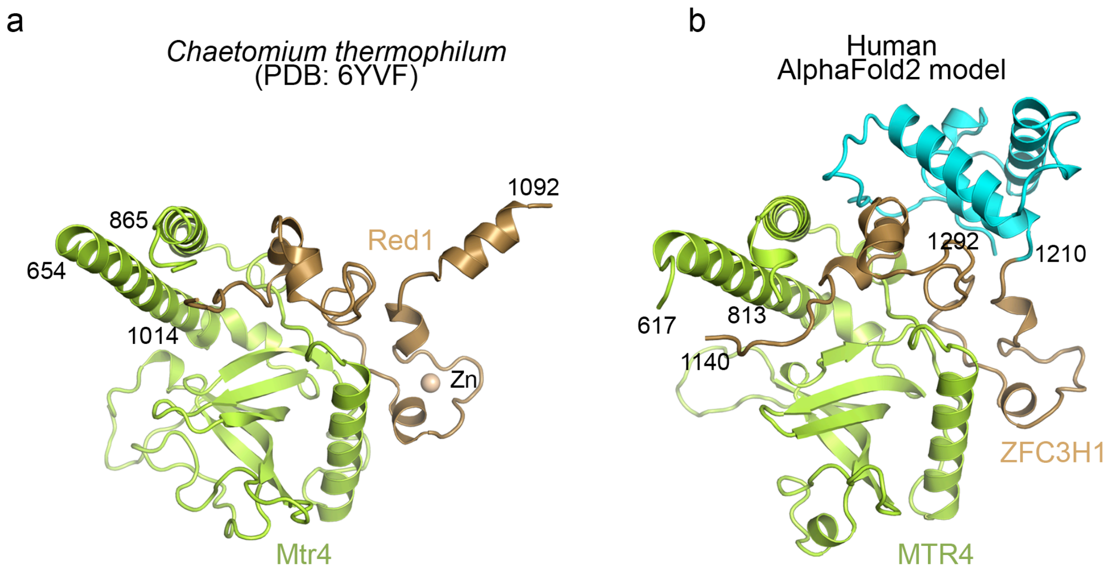

Supplementary Figure 15
a Crystal structure of the *Cheatomium thermophiulum* Mtr4-Red1 complex (PDB code- 6YVF- http://doi.org/10.2210/pdb6YVF/pdb). **b** AlphaFold2 model of human MTR4-ZFC3H1 complex. Only residues with pLDDT score above 70 are shown. The MTR4-binding region of ZFC3H1 packs against an additional helical domain shown in cyan.

Supplementary Table 1. NMR and refinement statistics for the minimal heterodimer Iss10/Red1.

	Iss10/Red1
**Number of structures in ensemble**	20
**Distance Constraints**	
Total	1993
Protein Intra-residue	692
Protein Inter-residue	
Sequential (|i-j|=1)	321
Short Range (1<|i-j|<4)	142
Medium Range (3<|i-j|<6)	79
Long Range (|i-j|>5)	74
Intermolecular	87
Ambiguous	528
Hydrogen bond	70
**Total dihedral angle constraints**	
Backbone (φ,ψ)	154 (77,77)
Sidechain (χ_1,χ_2)	58 (46,12)
**Structure Statistics**	
**Violations (mean and SD)**	
Distance Constraints (Å)[a]	0.023 ± 0.002
Dihedral Angle Constraints (°)[b]	2.28 ± 0.09
RDC, Q[c]	0.24 ± 0.01
**Deviations from idealized geometry**	
Bond Lengths (Å)	0.003 ± 0.000
Bond Angles (°)	0.45 ± 0.01
Impropers (°)	1.2 ± 0.1
**Ramachandran plot (%)[d]**	
Most favored	92.9 (88.2)
Additionally favored	6.6 (10.7)
Generally allowed	0.4 (0.7)
Disallowed	0.1 (0.4)
**Average pairwise rmsd (Å)**	
Protein backbone 2° structure	0.5 ± 0.2
Protein heavy 2° structure	1.0 ± 0.2
Protein backbone all	1.2 ± 0.3
Protein heavy all	1.3 ± 0.2

[a] No distance violation greater than 0.5 Å.
[a] No dihedral restraint violation greater than 10°.
[c] Calculated for each structure in the ensemble using the method described by [3].
[d] Determined by using PROCHECK-NMR[4]. Values do not include the expression tag. Values that include the tag are in parentheses.

Supplementary Table 2. Crystallographic data collection and refinement statistics.

	**Red1-Ars2**
**Data collection**	
Space group	$P2_1$
Cell dimensions	
a, b, c (Å)	64.7, 128.3, 89.3
α β γ (°)	90, 108, 90
Resolution (Å)	85-2.8 (3-2.8)[a]
R_{merge} (%)	29.6 (148)
I / σI	5.3 (1.5)
$CC_{½}$ (%)	99 (55)
Completeness (%)	76 (18)
Redundancy	5.6 (6.2)
**Refinement**	
Resolution (Å)	84.8-2.8
No. reflections	25473
R_{work} / R_{free}	0.245/0.302
B-factors	53
R.m.s. deviations	
Bond lengths (Å)	0.002
Bond angles (°)	1.17

[a] Values in parentheses are for highest resolution shell.

Supplementary Table 3. List of *S. pombe* strains used in this study.

Name	Genotype	Source
SPV8	*h+ leu1-32 ori1 ade6 M216 ura4-D18 imr1R::ura4+*	Lab Stock
SPV2180	*h+ leu1-32 ori1 ade6 M216 ura4-D18 imr1R::ura4+ red1::TAP KanR*	Lab Stock
SPV3106	*h+ leu1-32 ori1 ade6 M216 ura4-D18 imr1R::ura4+ red1Δ::hphR*	Lab Stock
SPV5699	*h+ leu1-32 ori1 ade6 M216 ura4-D18 red1::TAP KanR*	This study
SPV5714	*h+ leu1-32 ori1 ade6 M216 ura4-D18 red1Δ30-207::TAP KanR*	This study
SPV5785	*h+ leu1-32 ori1 ade6 M216 ura4-D18 red1E32R::TAP KanR Clone 29*	This study
SPV5786	*h+ leu1-32 ori1 ade6 M216 ura4-D18 red1E32R::TAP KanR Clone 30*	This study
SPV5787	*h+ leu1-32 ori1 ade6 M216 ura4-D18 red1L205R::TAP KanR Clone 4*	This study
SPV5788	*h+ leu1-32 ori1 ade6 M216 ura4-D18 red1L205R::TAP KanR Clone 14*	This study
SPV5994	*h- leu1-32 ori1 ade6 M216 ura4-D18 imr1R::ura4+ iss10Δ::hphR*	This study
SPV5995	*h+ leu1-32 ori1 ade6 M216 ura4-D18 imr1R::ura4+ iss10Δ::hphR*	This study
SPV6013	*h- leu1-32 ori1 ade6 M216 ura4-D18 imr1R::ura4+ red1L205R::TAP KanR iss10Δ::hphR Clone 3*	This study
SPV6015	*h+ leu1-32 ori1 ade6 M216 ura4-D18 imr1R::ura4+ red1L205R::TAP KanR iss10Δ::hphR Clone 7*	This study
SPV6017	*h+ leu1-32 ori1 ade6 M216 ura4-D18 red1::TAP KanR iss10::GFP NatR Clone 5*	This study
SPV6018	*h+ leu1-32 ori1 ade6 M216 ura4-D18 red1::TAP KanR iss10::GFP NatR Clone 6*	This study
SPV6020	*h+ leu1-32 ori1 ade6 M216 ura4-D18 red1L205R::TAP KanR iss10::GFP NatR Clone 5*	This study
SPV6021	*h+ leu1-32 ori1 ade6 M216 ura4-D18 red1L205R::TAP KanR iss10::GFP NatR Clone 6*	This study
SPV6023	*h- leu1-32 ori1 ade6 M216 ura4-D18 imr1R::ura4+ ars2-GFP Clone 1*	This study
SPV6024	*h- leu1-32 ori1 ade6 M216 ura4-D18 imr1R::ura4+ ars2-GFP Clone 2*	This study
SPV6026	*h+ leu1-32 ori1 ade6 M216 ura4-D18 red1E32R::TAP KanR ars2-GFP Clone 1*	This study
SPV6027	*h+ leu1-32 ori1 ade6 M216 ura4-D18 red1E32R::TAP KanR ars2-GFP Clone 2*	This study
SPV6029	*h+ leu1-32 ori1 ade6 M216 ura4-D18 imr1R::ura4+ red1::TAP KanR ars2-GFP Clone 1*	This study
SPV6030	*h+ leu1-32 ori1 ade6 M216 ura4-D18 imr1R::ura4+ red1::TAP KanR ars2-GFP Clone 2*	This study
SPV6065	*h+ leu1-32 ori1 ade6 M216 ura4-D18 imr1R::ura4+ red1Δ::hphR + plasmide pRJ 81 XL Clone 1*	This study
SPV6066	*h+ leu1-32 ori1 ade6 M216 ura4-D18 imr1R::ura4+ red1Δ::hphR + plasmide pRJ 81 XL Clone 2*	This study
SPV6067	*h+ leu1-32 ori1 ade6 M216 ura4-D18 imr1R::ura4+ red1Δ::hphR + plasmide pRJ 81 XL Clone 3*	This study
SPV6068	*h+ leu1-32 ori1 ade6 M216 ura4-D18 imr1R::ura4+ red1Δ::hphR + plasmide pRJ 81 XL red1-3HA Clone 1*	This study
SPV6069	*h+ leu1-32 ori1 ade6 M216 ura4-D18 imr1R::ura4+ red1Δ::hphR + plasmide pRJ 81 XL red1-3HA Clone 2*	This study
SPV6070	*h+ leu1-32 ori1 ade6 M216 ura4-D18 imr1R::ura4+ red1Δ::hphR + plasmide pRJ 81 XL red1-3HA Clone 3*	This study
SPV6071	*h+ leu1-32 ori1 ade6 M216 ura4-D18 imr1R::ura4+ red1Δ::hphR + plasmide pRJ 81 XL red1-452-524Δ-3HA Clone 1*	This study
SPV6072	*h+ leu1-32 ori1 ade6 M216 ura4-D18 imr1R::ura4+ red1Δ::hphR + plasmide pRJ 81 XL red1-452-524Δ-3HA Clone 2*	This study
SPV6073	*h+ leu1-32 ori1 ade6 M216 ura4-D18 imr1R::ura4+ red1Δ::hphR + plasmide pRJ 81 XL red1-452-524Δ-3HA Clone 3*	This study
SPV6074	*h+ leu1-32 ori1 ade6 M216 ura4-D18 imr1R::ura4+ red1Δ::hphR + plasmide pRJ 81 XL red1-L481R-I485R-3HA Clone 1*	This study
SPV6075	*h+ leu1-32 ori1 ade6 M216 ura4-D18 imr1R::ura4+ red1Δ::hphR + plasmide pRJ 81 XL red1-L481R-I485R-3HA Clone 2*	This study
SPV6076	*h+ leu1-32 ori1 ade6 M216 ura4-D18 imr1R::ura4+ red1Δ::hphR + plasmide pRJ 81 XL red1-L481R-I485R-3HA Clone 3*	This study
SPV6095	*h+ leu1-32 ori1 ade6 M216 ura4-D18 imr1R::ura4+ red1::TAP-KanR + plasmide pRJ 81 XL Clone 1*	This study
SPV6096	*h+ leu1-32 ori1 ade6 M216 ura4-D18 imr1R::ura4+ red1::TAP-KanR + plasmide pRJ 81 XL Clone 2*	This study
SPV6098	*h+ leu1-32 ori1 ade6 M216 ura4-D18 imr1R::ura4+ red1::TAP-KanR + plasmide pRJ 81 XL red1-3HA Clone 4*	This study
SPV6099	*h+ leu1-32 ori1 ade6 M216 ura4-D18 imr1R::ura4+ red1::TAP-KanR + plasmide pRJ 81 XL red1-3HA Clone 5*	This study
SPV6101	*h+ leu1-32 ori1 ade6 M216 ura4-D18 imr1R::ura4+ red1::TAP-KanR + plasmide pRJ 81 XL red1-452-524Δ-3HA Clone 7*	This study
SPV6102	*h+ leu1-32 ori1 ade6 M216 ura4-D18 imr1R::ura4+ red1::TAP-KanR + plasmide pRJ 81 XL red1-452-524Δ-3HA Clone 8*	This study
SPV6104	*h+ leu1-32 ori1 ade6 M216 ura4-D18 imr1R::ura4+ red1::TAP-KanR + plasmide pRJ 81 XL red1-L481R-I485R-3HA Clone 10*	This study
SPV6105	*h+ leu1-32 ori1 ade6 M216 ura4-D18 imr1R::ura4+ red1::TAP-KanR + plasmide pRJ 81 XL red1-L481R-I485R-3HA Clone 11*	This study
SPV6107	*h$^+$ leu1-32 ura4-D18 ade6-210 otr1R(Sph1)::ade6$^+$ 3xFlag-clr4 red1::13Myc-hphR + plasmide pJR 81 XL Clone 1*	This study
SPV6108	*h$^+$ leu1-32 ura4-D18 ade6-210 otr1R(Sph1)::ade6$^+$ 3xFlag-clr4 red1::13Myc-hphR + plasmide pJR 81 XL Clone 2*	This study
SPV6110	*h$^+$ leu1-32 ura4-D18 ade6-210 otr1R(Sph1)::ade6$^+$ 3xFlag-clr4 red1::13Myc-hphR + plasmide pJR 81 XL red1-3HA Clone 4*	This study
SPV6111	*h$^+$ leu1-32 ura4-D18 ade6-210 otr1R(Sph1)::ade6$^+$ 3xFlag-clr4 red1::13Myc-hphR + plasmide pJR 81 XL red1-3HA Clone 5*	This study
SPV6113	*h$^+$ leu1-32 ura4-D18 ade6-210 otr1R(Sph1)::ade6$^+$ 3xFlag-clr4 red1::13Myc-hphR + plasmide pJR 81 XL red1-452-524Δ-3HA Clone 7*	This study
SPV6114	*h$^+$ leu1-32 ura4-D18 ade6-210 otr1R(Sph1)::ade6$^+$ 3xFlag-clr4 red1::13Myc-hphR + plasmide pJR 81 XL red1-452-524Δ-3HA Clone 8*	This study
SPV6116	*h$^+$ leu1-32 ura4-D18 ade6-210 otr1R(Sph1)::ade6$^+$ 3xFlag-clr4 red1::13Myc-hphR + plasmide pJR 81 XL red1-L481R-I485R-3HA Clone 10*	This study
SPV6117	*h$^+$ leu1-32 ura4-D18 ade6-210 otr1R(Sph1)::ade6$^+$ 3xFlag-clr4 red1::13Myc-hphR + plasmide pJR 81 XL red1-L481R-I485R-3HA Clone 11*	This study

Supplementary Table 4. List of primers used for the *S. pombe* analysis.

gene	Sequence 5' > 3'
**strain construction**	
Ars2-GFP tag for	TTCTATAGAACTTATCAAGATTTGGATGCGCCTAACCAGGAGGTTCCAGAATTAGATTA Ccggatccccgggttaattaa
Ars2-GFP tag rev	TAACGTCTATACAGGAAATGATATTAACCAAAAATCTTTCTGACTAAGAACATAAATTT Agaattcgagctcgtttaaac
Iss10-GFP tag for	AACGACTATGAATTAGCAGAGGAAATTGAGAGGCGTCGTCATCATAGCTTTTATGATT ATcggatccccgggttaattaa
Iss10-GFP tag rev	TCAAATATTCCGCCAAGCAATTTTTAAATTGTTGCGCGCACTTACGGAAGAGTATAGC TAgaattcgagctcgtttaaac
red1Δ30-207 for	GGTCAATAAATCTCGATGAGTTGCGAAAAAAGGCTTTAGAATCAAAAAAGAAGAATGA AGAAGATGAGAGTAATGACTCTCGCCAGGGTTTTCCCAGTCACGAC
red1Δ30-207 rev	AGTCCGAGCTTTAAAAATAAAGTATGAACAACCGAAGGCTCGATGCCCTCAGCAATA AAATCGTTAAAGCGCACTCCTACAGCGGATAACAATTTCACACAGGA
red1-3HA tag for	acgtcccggactatgcaggatcctatccatatgacgttccagattacgctTAGGGATCCCCGGGTAAA
red1-3HA tag rev	catacggatagcccgcatagtcaggaacatcgtatgggtaaccggcggaaATGGAAACCTTTGCAACAGG
red1Δ452-524 for	GTTGCATGTTTACCTAAAG
red1Δ452-524 rev	TAATATCTGCTCAGTTGTATC
red1 L481R-I485R for	cttaagaGAAAAAGAGGAAGGAGAAC
red1 L481R-I485R for	ttatttctAATGTCAATCTCGGATTTTTTAATC
**genotyping**	
red1Δ30-207 for	TTGCTACATTCAGTATACCTGTTCA
red1Δ30-207 rev	TTCGCTAATTTCGCCATCTT
Ars2-GFP tag for	GGATCCCAAAGTGGTAAGCA
Ars2-GFP tag rev	TTCTATCTGTGCAGCGTTCG
Iss10-GFP tag for	GCCTCCTATTGAAACGCACT
Iss10-GFP tag rev	TGCGAAACCAATTTATGCAA
red1-3HA tag for	TTGAAATATCGGTCGCTTACCT
red1-3HA tag rev	CAGTACTGGCAAGGGAGACATTCC
red1Δ452-524 for	gtagctgcggttgacaacaa
red1 L481R-I485R for	GAG ATT GAC ATT AGA AAT AAC TTA AGA
red1 crib rev	atctgtttccgtcgtcccat
**qPCR**	
tub1 for	GTACTGGCCCATACCGTGAT
tub1 rev	CGAATGGAAGACGAGAAAGC
ssm4 for	TGCAAGAGGAAACTCAAAGG
ssm4 rev	TTCCTCCTCCACTTGTTTTGA
spo5 for	TTATGGCGTGCTCGTGAATA
spo5 rev	TTCGACTCCAATGGCACATA
mug8 for	GGCTGACCAAATGAATGCTT
mug8 rev	GATCTGCCCAACCAACAATC
mug9 for	CTCTGTTGGCTCCTTCAAGC
mug9 rev	TAATGTTTGAGCACGCATGG
mcp5 for	TTTGGATTAACCCGCATTGT
mcp5 rev	TGGCGTTCTTTAAGGCATCT
rec8 for	GTTGAAGTTGGACGGGATGT
rec8 rev	TTCTACCCTACTCGGCATCG
crs1 for	AGGCCGACGGAAATTTTATG
crs1 rev	ACCATTTGTGGGCAAAGAAC
mei4 for	AAAAGCGACCTTCAAGCAAA
mei4 rev	TTGCATCGTTTGAGACTTCG
cti6 PROMPT for	CATCGGAGAACCTGGTTTGT
cti6 PROMPT rev	TGCATTCCCAAATCAAGTC
rlp402 PROMPT for	GAAAGAGGAACAATGTTTAGAAGTC
rlp402 PROMPT rev	AAGCCCTACGATTGCTGTG
pho1 for	GACTTCCCGTAGCCCCTATC
pho1 rev	TCCAACAGCATCGAAAGCAG
pho1ncRNA for	TGAGATTTACGGGAAGTGCC
pho1ncRNA rev	AGCGACTATTCCCCTGTTGT
byr2 for	TCATCTTCACCGGAACGCG
byr2 rev	AGCGGACACTTCGTTTCTG
nam1-L for	CCAGTAGTATCTGAACGGTG
nam1-L rev	CATGGACGTGGAAACTCTCG
nam1 for	TCGGCAATTCAACCATAACA
nam1 rev	TGGCTTCATTCCTTCCTTTG
**RT**	
RT pho1	AATAACTCGACACGGCCACT
RT pho1ncRNA	CAAACGAAACCAACAACGCC
RT byr2	AGCGGACACTTCGTTTCTG
RT nam1	CAATCACGACGGATCGTACA

Supplementary Reference

1. Soltysova, M. *et al.* Structural insight into DNA recognition by bacterial transcriptional regulators of the SorC/DeoR family. *Acta Crystallogr. Sect. D Struct. Biol.* **77**, 1411-1424 (2021).
2. Terwilliger, T. C. Maximum-likelihood density modification. *Acta Crystallogr. Sect. D Biol. Crystallogr.* **56**, 965-972 (2000).
3. Cornilescu, G., Marquardt, J. L., Ottiger, M. & Bax, A. Validation of protein structure from anisotropic carbonyl chemical shifts in a dilute liquid crystalline phase. *J. Am. Chem. Soc.* **120**, 6836-6837 (1998).
4. Laskowski, R. A., Rullmann, J. A. C., MacArthur, M. W., Kaptein, R. & Thornton, J. M. AQUA and PROCHECK-NMR: Programs for checking the quality of protein structures solved by NMR. *J. Biomol. NMR* **8**, 477-486 (1996).
