# Supplementary material for: Structural analysis of Red1 as a conserved scaffold of the RNA-targeting MTREC/PAXT complex
Source: Nat Commun. 2022 Aug 24;13:4969. doi: 10.1038/s41467-022-32542-3 (PMC9402713; doi:10.1038/s41467-022-32542-3)
Supplement: Supplementary file 3 — Reporting Summary [file 41467_2022_32542_MOESM3_ESM.pdf]

nature portfolio

Corresponding author(s): Cameron D. Mackereth, André Verdel and Jan Kadlec

Last updated by author(s): Jul 22, 2022

Reporting Summary

Nature Portfolio wishes to improve the reproducibility of the work that we publish. This form provides structure for consistency and transparency in reporting. For further information on Nature Portfolio policies, see our Editorial Policies and the Editorial Policy Checklist.

Statistics

For all statistical analyses, confirm that the following items are present in the figure legend, table legend, main text, or Methods section.

n/a	Confirmed	
☐	☒	The exact sample size (n) for each experimental group/condition, given as a discrete number and unit of measurement
☐	☒	A statement on whether measurements were taken from distinct samples or whether the same sample was measured repeatedly
☐	☒	The statistical test(s) used AND whether they are one- or two-sided<br>*Only common tests should be described solely by name; describe more complex techniques in the Methods section.*
☒	☐	A description of all covariates tested
☒	☐	A description of any assumptions or corrections, such as tests of normality and adjustment for multiple comparisons
☐	☒	A full description of the statistical parameters including central tendency (e.g. means) or other basic estimates (e.g. regression coefficient) AND variation (e.g. standard deviation) or associated estimates of uncertainty (e.g. confidence intervals)
☐	☒	For null hypothesis testing, the test statistic (e.g. F, t, r) with confidence intervals, effect sizes, degrees of freedom and P value noted<br>*Give P values as exact values whenever suitable.*
☒	☐	For Bayesian analysis, information on the choice of priors and Markov chain Monte Carlo settings
☒	☐	For hierarchical and complex designs, identification of the appropriate level for tests and full reporting of outcomes
☒	☐	Estimates of effect sizes (e.g. Cohen's d, Pearson's r), indicating how they were calculated

Our web collection on statistics for biologists contains articles on many of the points above.

Software and code

Policy information about availability of computer code

Data collection	For X-ray data collection at the ESRF, MXcuBE3 ESRF software was used. Raw live cell Images were acquired with a Zeiss Apotome microscope (Carl Zeiss MicroImaging). Raw immunofluoerescence images were acquired using the AxioVision software (Carl Zeiss MicroImaging).
Data analysis	All the software used for NMR and X-ray data analysis and structure modeling is publicly available with full referencing in the manuscript. ITC data were analysed using using the Origin software, version 7.0 (MicroCal). The MALLS data analysis was carried out with the software ASTRA, v5.4.3.20 (Wyatt, Santa-Barbara, USA). Raw live cell and immunofleuorescent images were analyzed using the AxioVision software (Carl Zeiss MicroImaging), and processed using ImageJ. Chemiluminescence signals were quantified with the imageLab software (Biorad) or the Fusion-Capt software (Vilber Lourmat).

For manuscripts utilizing custom algorithms or software that are central to the research but not yet described in published literature, software must be made available to editors and reviewers. We strongly encourage code deposition in a community repository (e.g. GitHub). See the Nature Portfolio guidelines for submitting code & software for further information.

Data

Policy information about availability of data

All manuscripts must include a data availability statement. This statement should provide the following information, where applicable:

- Accession codes, unique identifiers, or web links for publicly available datasets
- A description of any restrictions on data availability
- For clinical datasets or third party data, please ensure that the statement adheres to our policy

The structure ensemble of the Red1 192-236-Iss10 1-45 heterodimer has been deposited at the Protein Data Bank (http://www.ebi.ac.uk/pdbe/) with accession ID 7QUU. Corresponding chemical shift assignments have been deposited in the Biological Magnetic Resonance Data Bank (http://bmrb.wisc.edu/) under BMRB

accession number 34702.
The atomic coordinates and structure factors of the S. pombe Ars2-Red1 complex have been deposited under the PDB accession codes 7QY5.
Source data are provided with this paper.
The primers used in this study are listed in Supplementary Table 4. The strains used in this study are listed in Supplementary Table 3.

Field-specific reporting

Please select the one below that is the best fit for your research. If you are not sure, read the appropriate sections before making your selection.

- [x] Life sciences
- [ ] Behavioural & social sciences
- [ ] Ecological, evolutionary & environmental sciences

For a reference copy of the document with all sections, see nature.com/documents/nr-reporting-summary-flat.pdf

Life sciences study design

All studies must disclose on these points even when the disclosure is negative.

Sample size	Sample size were established according to the effects observed in the preliminary experiments. Number of biological replicates minimum of 2 for the co-immunoprecipiations and 3 for all of the other experiments; Cell numbers were determined by classical optical density and/or cell counting with the use of Zeiss Apotome microscope (Carl Zeiss MicroImaging) and using a 63X oil immersion objective with a numerical aperture of 1.4 (WD 190, DICIII and GFP filters). Raw images were analyzed using the AxioVision software (Carl Zeiss MicroImaging), and processed using ImageJ.
Data exclusions	No data was excluded from the study
Replication	Number of independent biological replicates were two for the co-IPs, three for the RT-qPCRs and for the RT-PCRs; each RT-qPCR was also done with two technical replicates, but all of the statistical analyses conducted on the RT-qPCR data were conducted only on the biological replicates.
Randomization	No experimental groups involved in this study, thus randomization was not performed.
Blinding	No experiments required bliding in this study.

Reporting for specific materials, systems and methods

We require information from authors about some types of materials, experimental systems and methods used in many studies. Here, indicate whether each material, system or method listed is relevant to your study. If you are not sure if a list item applies to your research, read the appropriate section before selecting a response.

Materials & experimental systems

n/a	Involved in the study	
[ ]	[x]	Antibodies
[ ]	[x]	Eukaryotic cell lines
[x]	[ ]	Palaeontology and archaeology
[x]	[ ]	Animals and other organisms
[x]	[ ]	Human research participants
[x]	[ ]	Clinical data
[x]	[ ]	Dual use research of concern

Methods

n/a	Involved in the study	
[x]	[ ]	ChIP-seq
[x]	[ ]	Flow cytometry
[x]	[ ]	MRI-based neuroimaging

Antibodies

Antibodies used	anti-TAP primary antibody (Thermo scientific #CAB1001) ; secondary antibody DyLight® 549 (VECTOR lab # DI 1549); anti-GFP -Sigma # 11814460001; secondary antibodies HRP goat anti-Mousse -Dako #P0447 and HRP goat anti-Rabbit -Dako #0448.
Validation	For the anti-TAP and anti-GFP the respective manufacturers indicate the proper reactivity of these ani-bodies using WB and immunofluorescence experiments. These anti-bodies have been extensively used for co-IPs and WB, including from S. pombe cell extracts. We further validated these anti-bodies in WB and IF using tagged and untagged cells.

Eukaryotic cell lines

Policy information about cell lines

Cell line source(s)	Fission yeast S. pombe cells
Authentication	Clones of S. pombe were selected by growth on selective media, genotyped by PCR and DNA sequencing.

Mycoplasma contamination	Not applicable
Commonly misidentified lines<br>(See ICLAC register)	Not applicable
