# Supplementary figures and images for: Structural analysis of Red1 as a conserved scaffold of the RNA-targeting MTREC/PAXT complex
Source: Nat Commun. 2022 Aug 24;13:4969. doi: 10.1038/s41467-022-32542-3 (PMC9402713; doi:10.1038/s41467-022-32542-3)

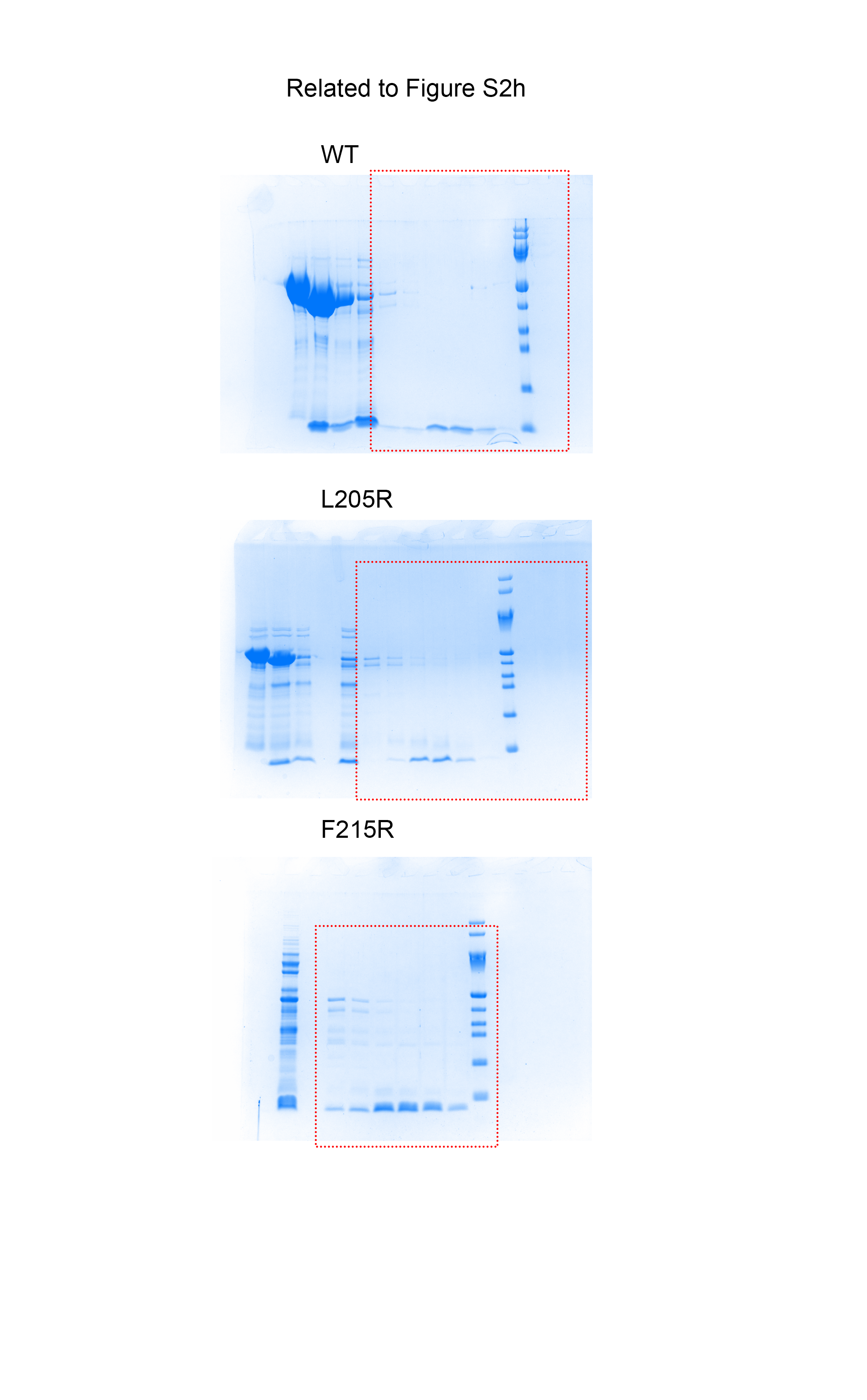

Supplement: Supplementary file 4 — Source Data [file 41467_2022_32542_MOESM4_ESM.zip › Source_data/SupFigure2/Sup2h.tif]

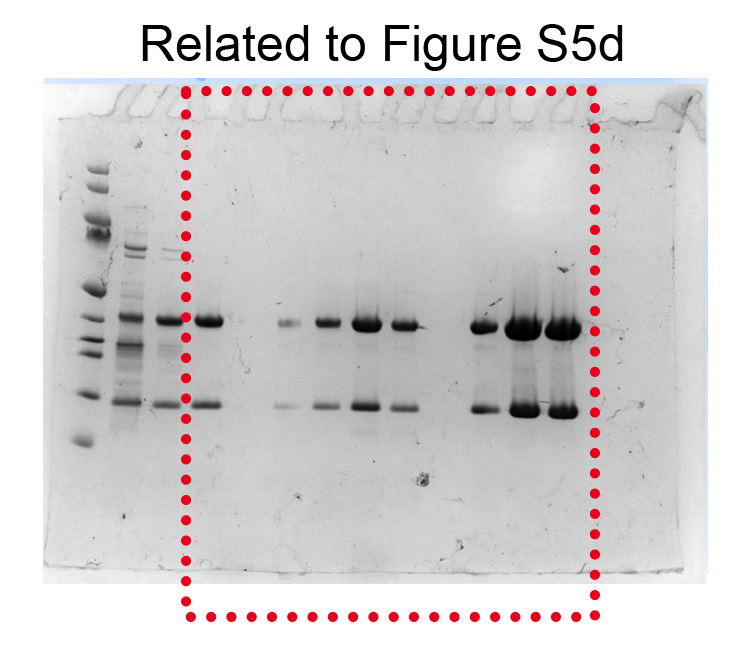

Supplement: Supplementary file 4 — Source Data [file 41467_2022_32542_MOESM4_ESM.zip › Source_data/SupFigure5/Sup5d.tif]

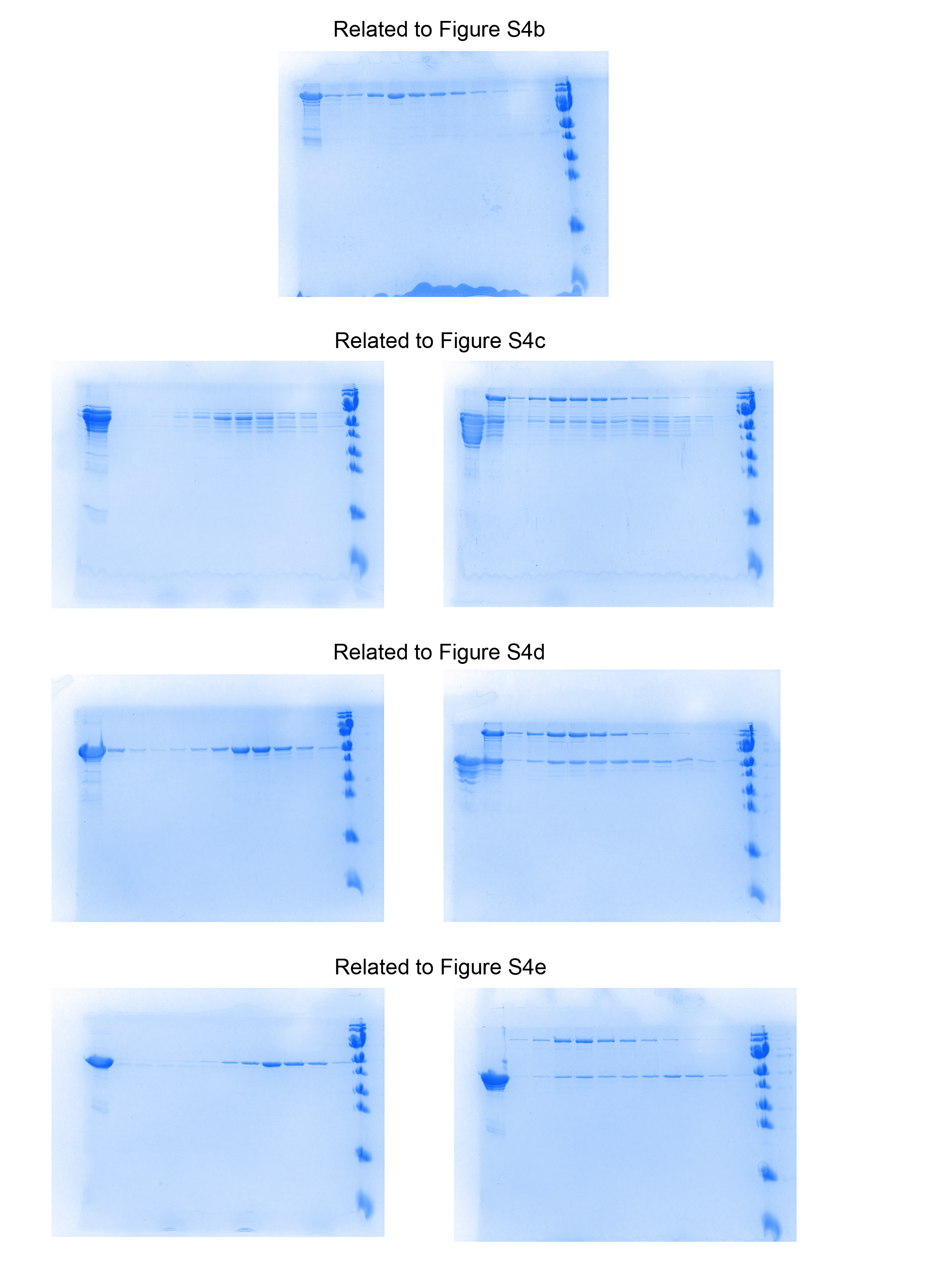

Supplement: Supplementary file 4 — Source Data [file 41467_2022_32542_MOESM4_ESM.zip › Source_data/SupFigure4/Sup4.tif]

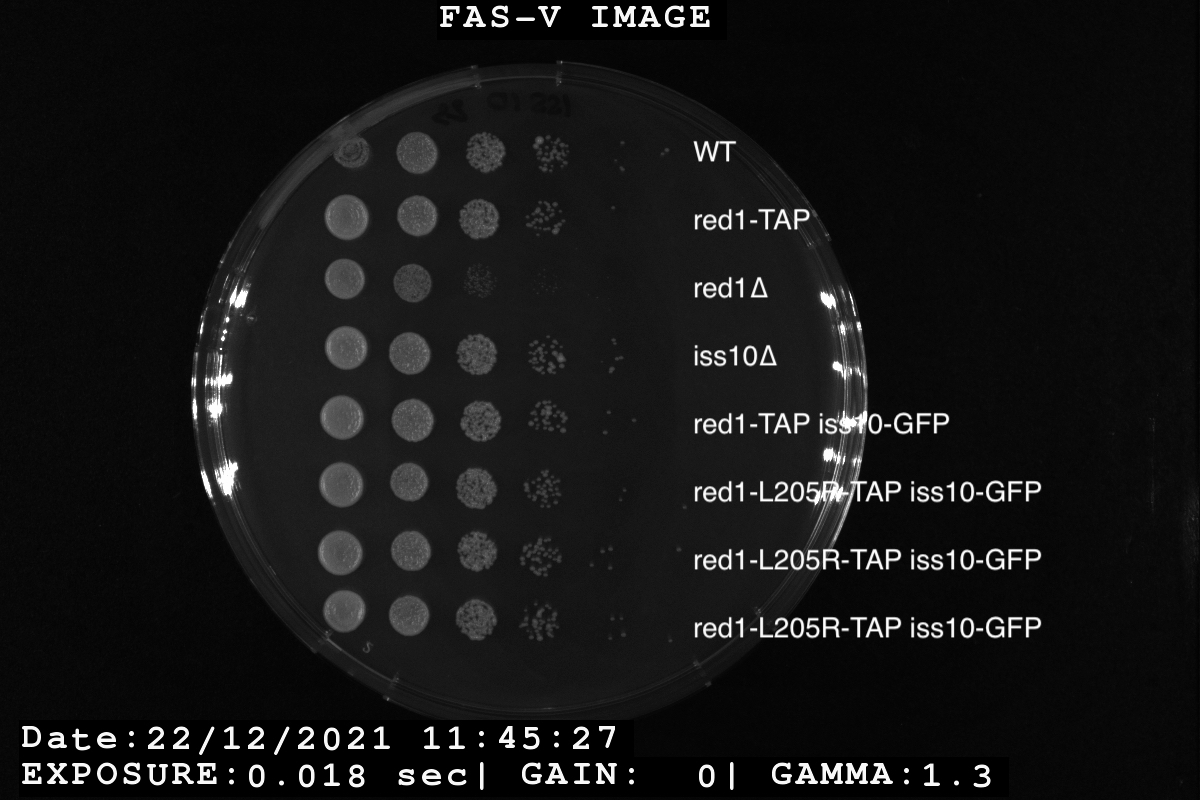

Supplement: Supplementary file 4 — Source Data [file 41467_2022_32542_MOESM4_ESM.zip › Source_data/Figure3&SupFigure3/3b/Fig3b_RM_25.jpeg]

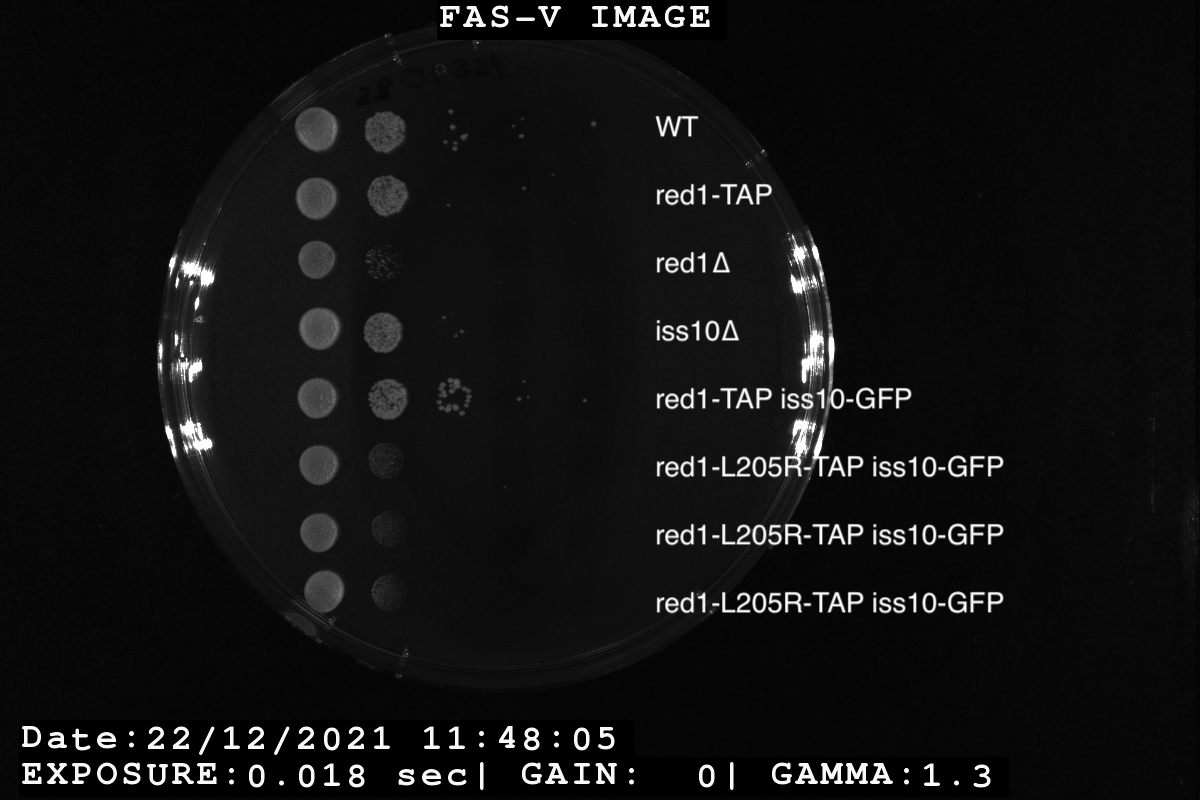

Supplement: Supplementary file 4 — Source Data [file 41467_2022_32542_MOESM4_ESM.zip › Source_data/Figure3&SupFigure3/3b/Fig3b_MM_25.jpeg]

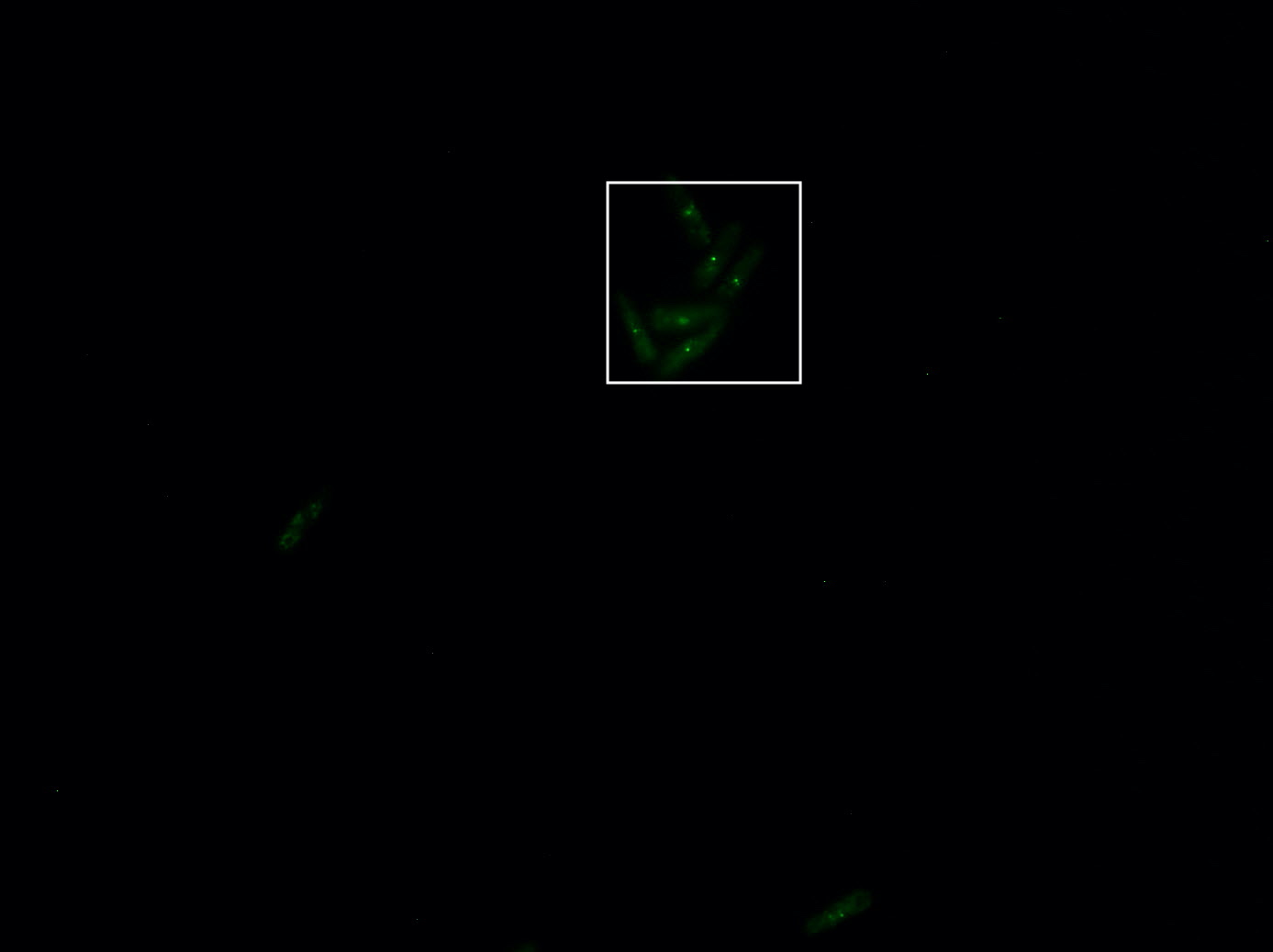

Supplement: Supplementary file 4 — Source Data [file 41467_2022_32542_MOESM4_ESM.zip › Source_data/Figure3&SupFigure3/3c/Fig3c_WT.JPG]

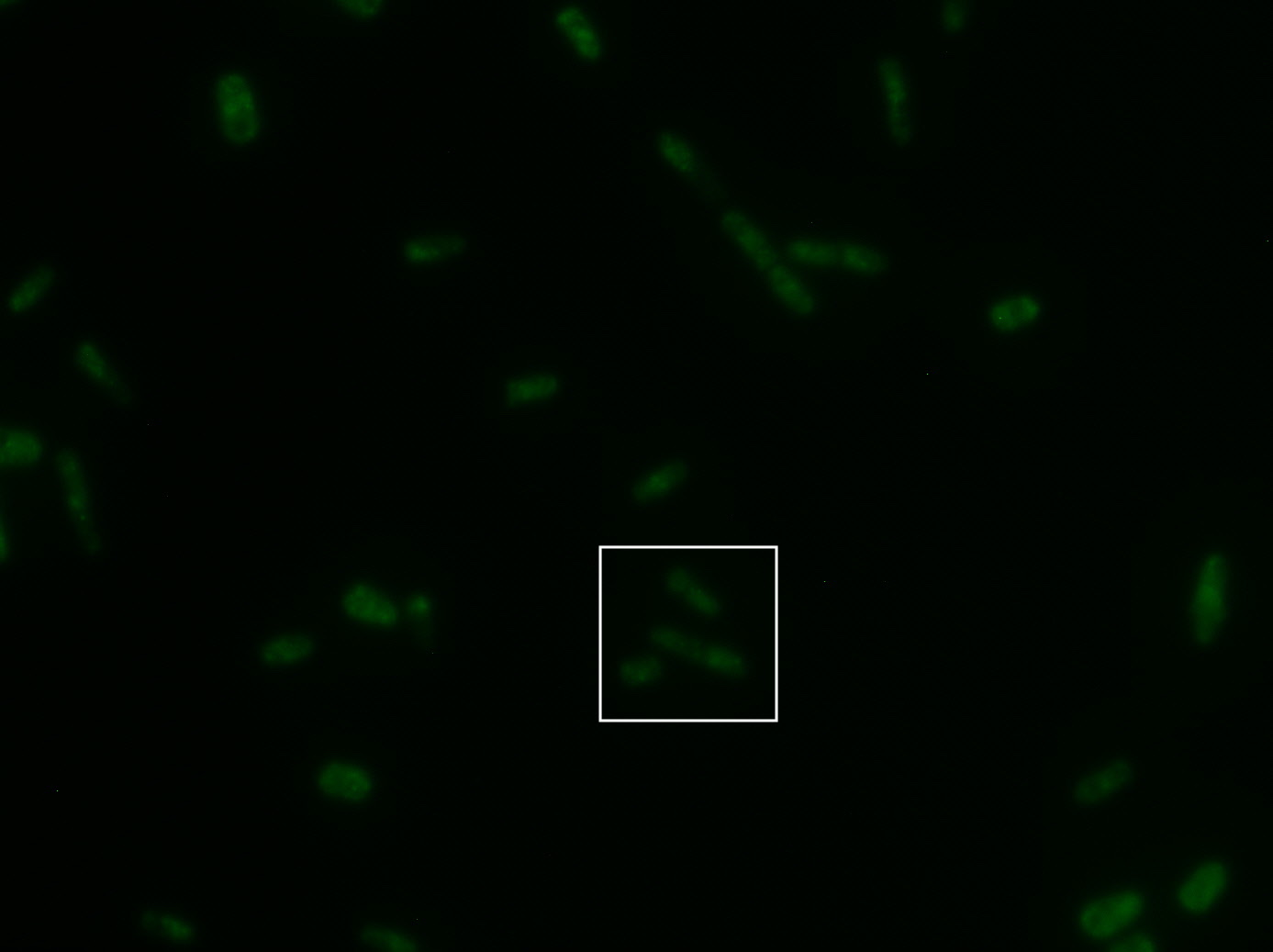

Supplement: Supplementary file 4 — Source Data [file 41467_2022_32542_MOESM4_ESM.zip › Source_data/Figure3&SupFigure3/3c/Fig3c_L205R.JPG]

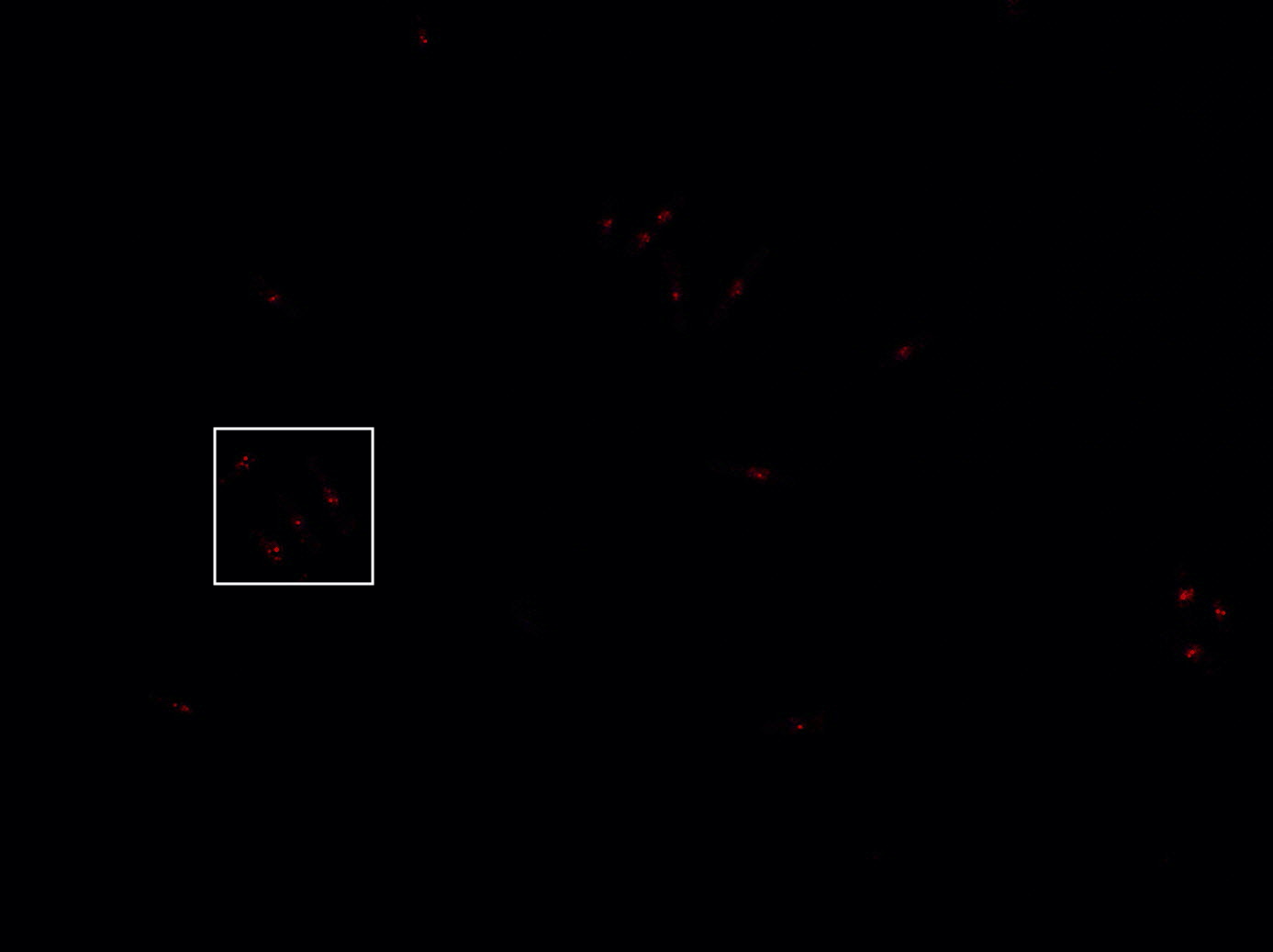

Supplement: Supplementary file 4 — Source Data [file 41467_2022_32542_MOESM4_ESM.zip › Source_data/Figure3&SupFigure3/3d/Fig3d_WT.JPG]

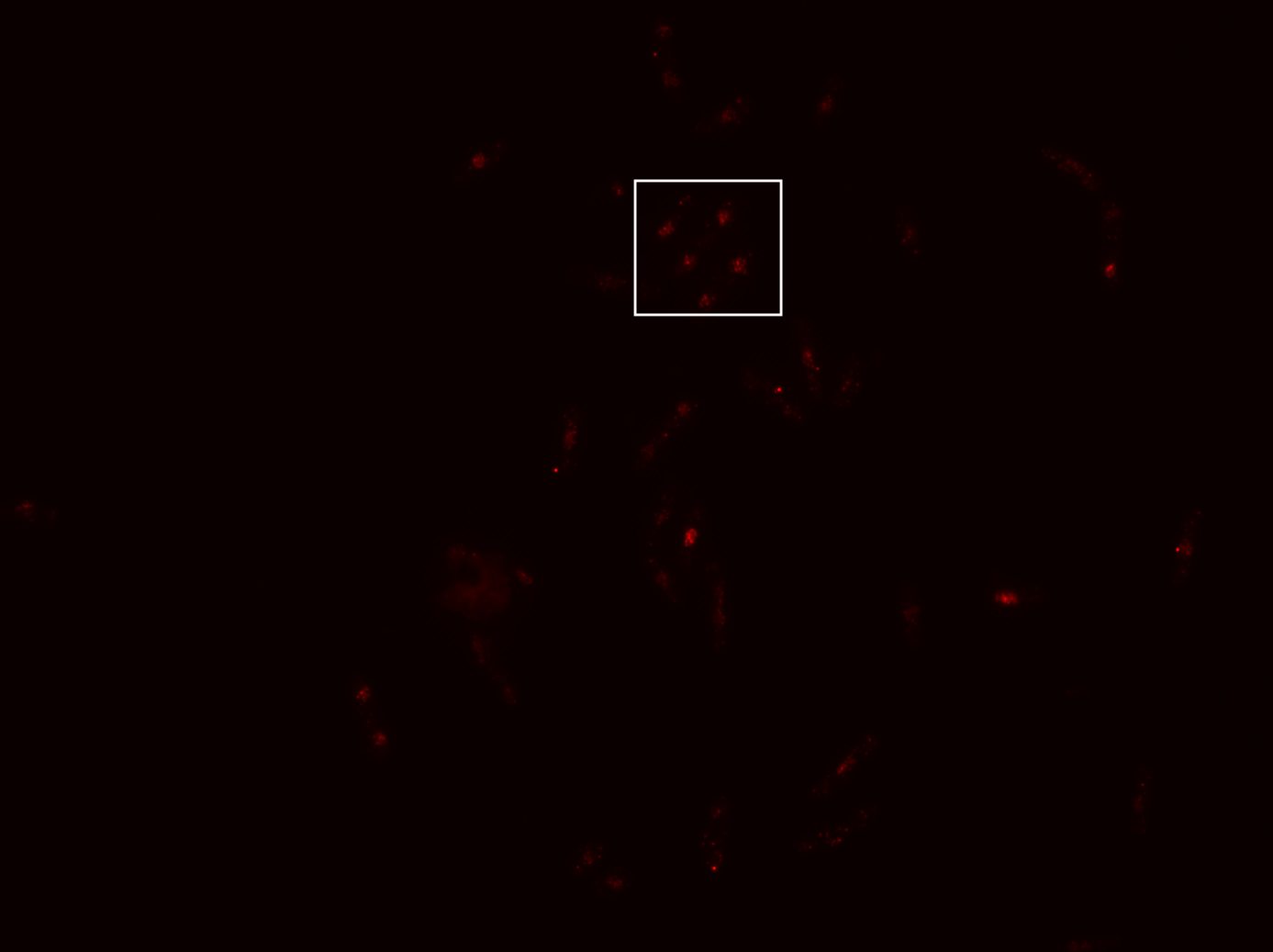

Supplement: Supplementary file 4 — Source Data [file 41467_2022_32542_MOESM4_ESM.zip › Source_data/Figure3&SupFigure3/3d/Fig3d_L205R.png]

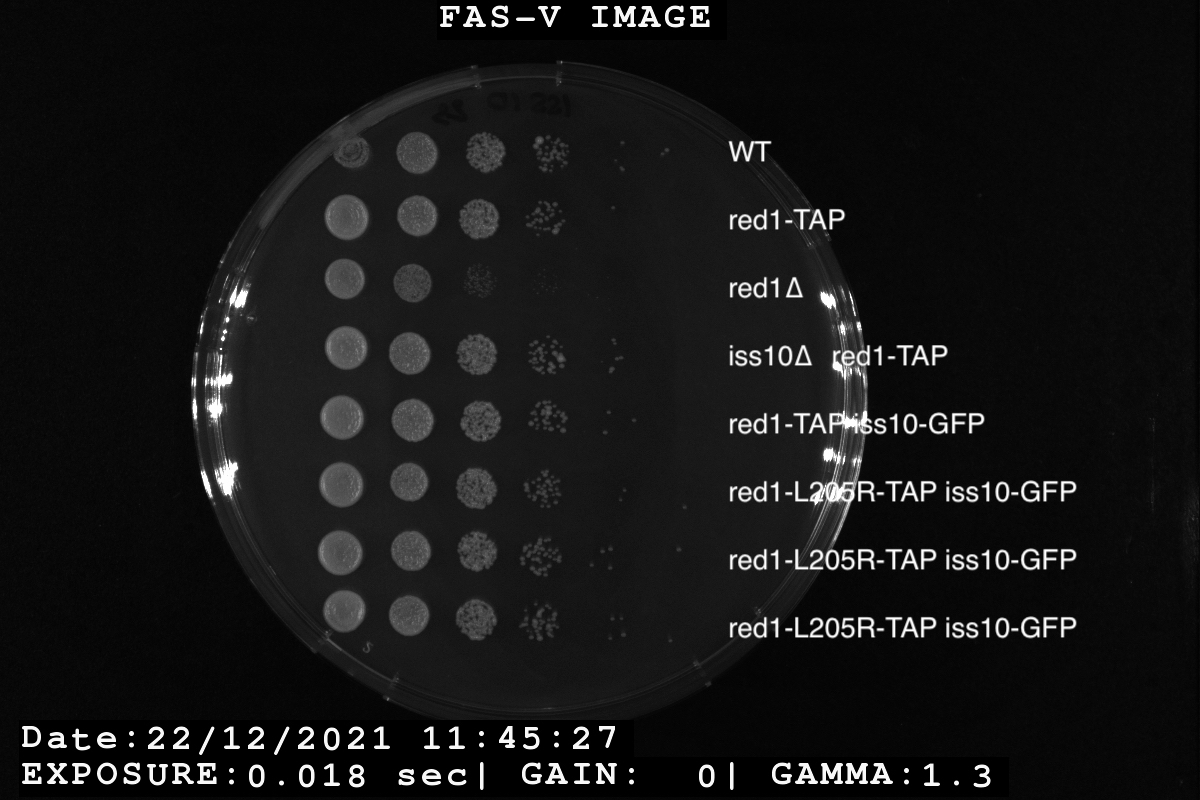

Supplement: Supplementary file 4 — Source Data [file 41467_2022_32542_MOESM4_ESM.zip › Source_data/Figure3&SupFigure3/Sup3b/SupFig3b_RM_25.jpeg]

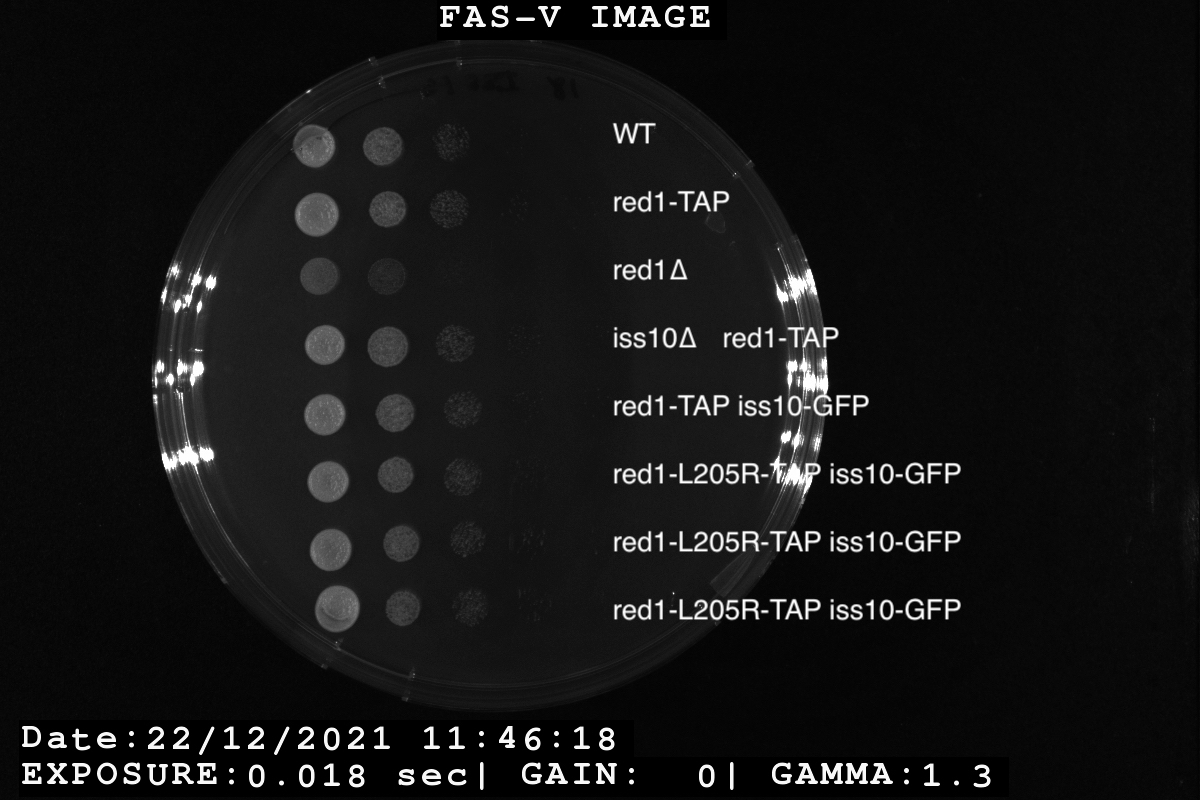

Supplement: Supplementary file 4 — Source Data [file 41467_2022_32542_MOESM4_ESM.zip › Source_data/Figure3&SupFigure3/Sup3b/SupFig3b_RM_18.jpeg]

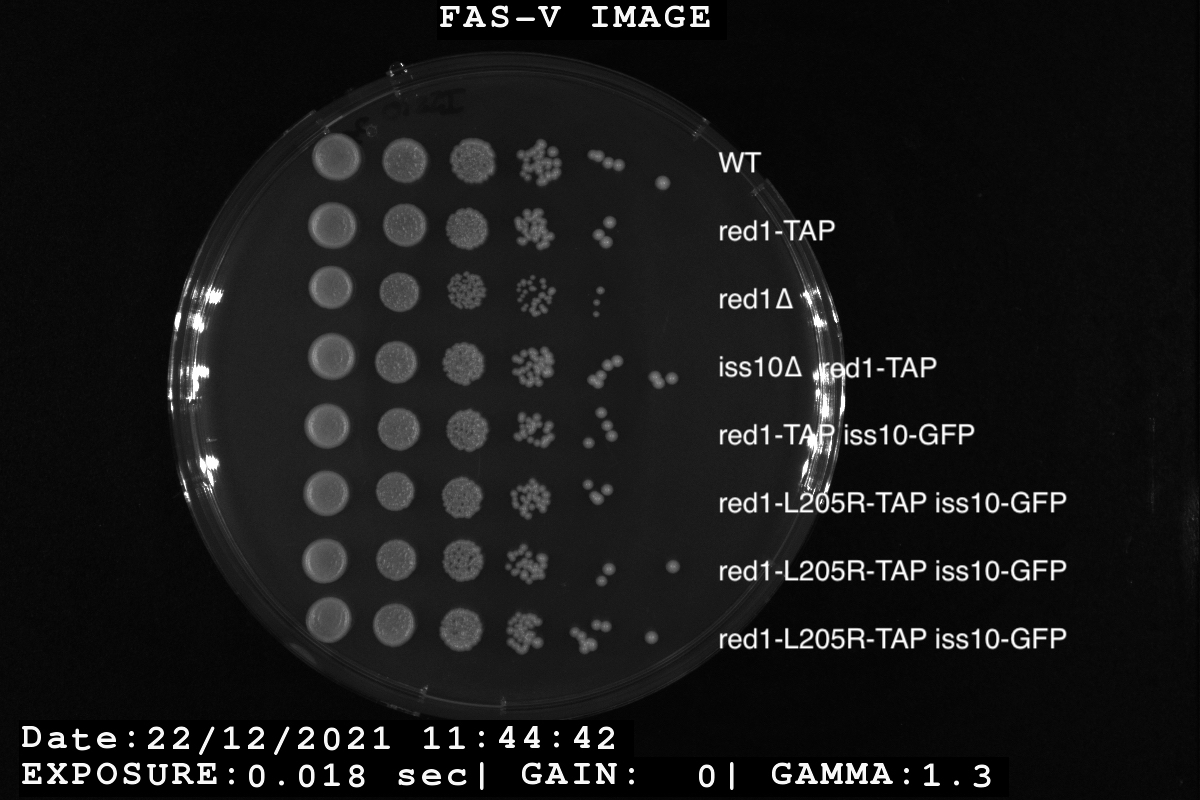

Supplement: Supplementary file 4 — Source Data [file 41467_2022_32542_MOESM4_ESM.zip › Source_data/Figure3&SupFigure3/Sup3b/SupFig3b_RM_30.jpeg]

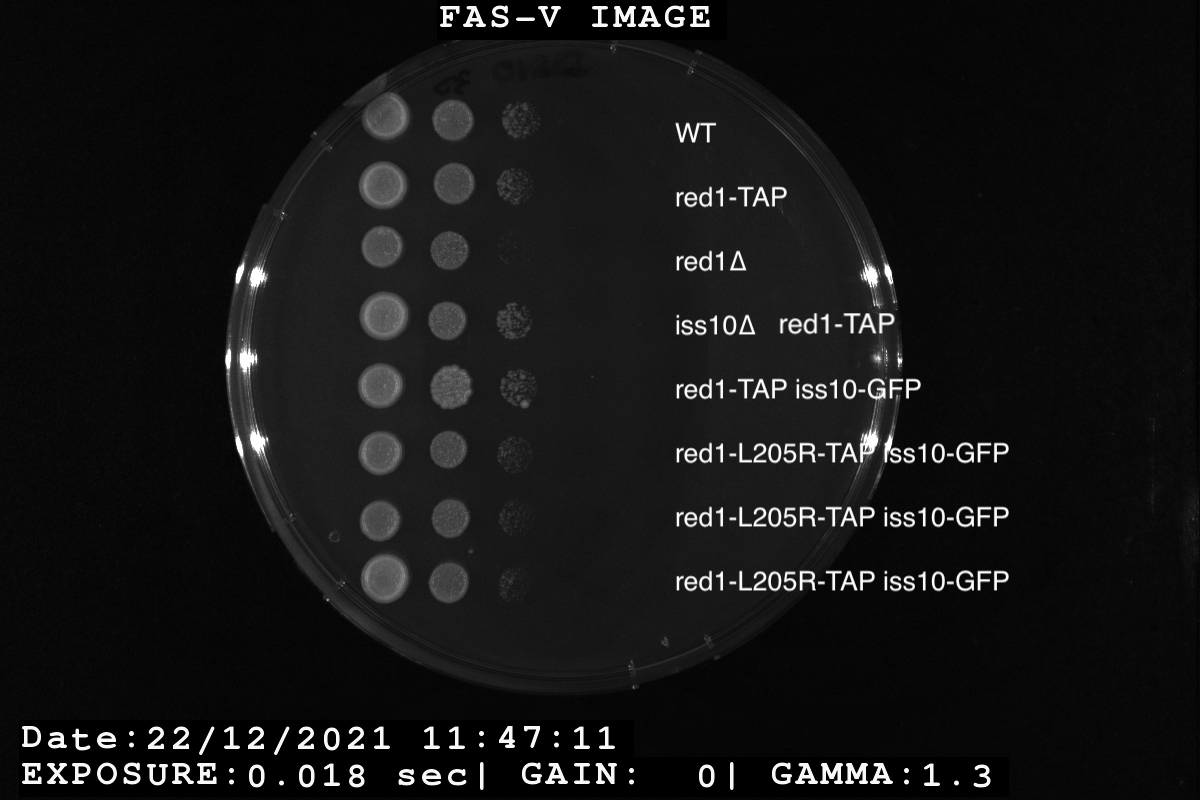

Supplement: Supplementary file 4 — Source Data [file 41467_2022_32542_MOESM4_ESM.zip › Source_data/Figure3&SupFigure3/Sup3c/SupFig3c_MM_30.jpeg]

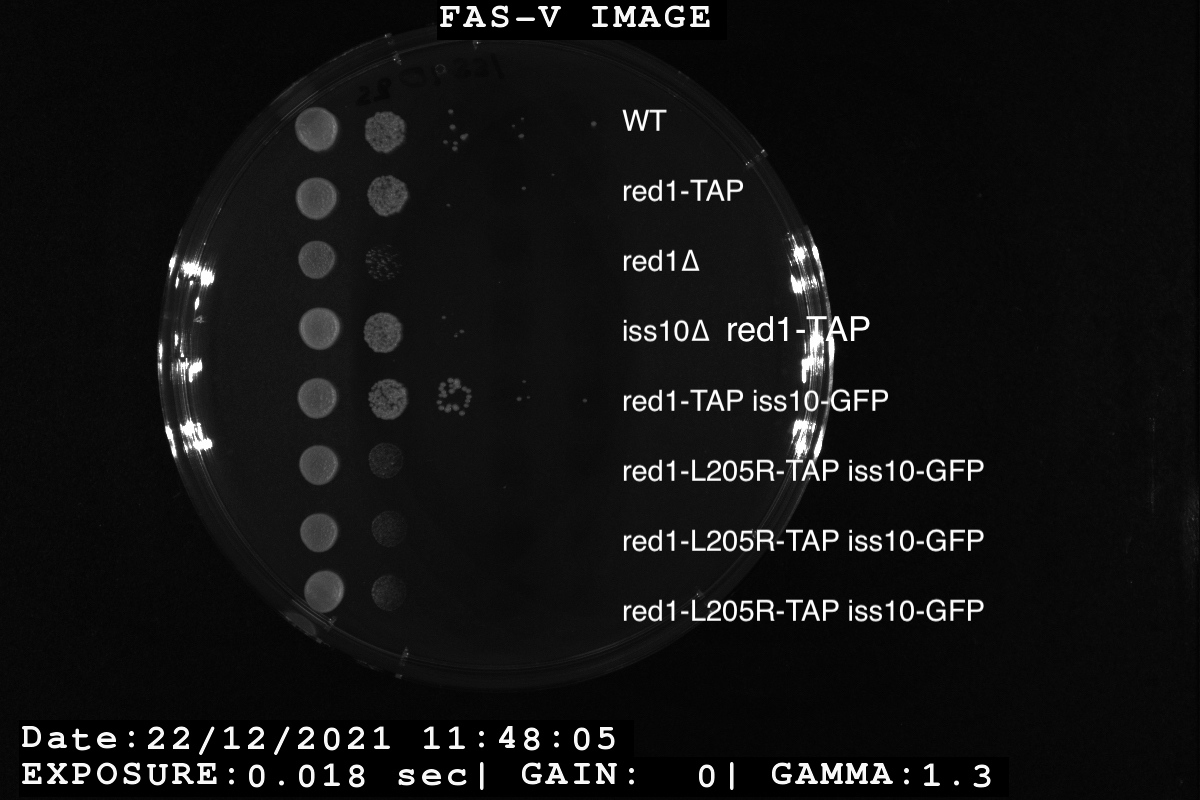

Supplement: Supplementary file 4 — Source Data [file 41467_2022_32542_MOESM4_ESM.zip › Source_data/Figure3&SupFigure3/Sup3c/SupFig3c_MM_25.jpeg]

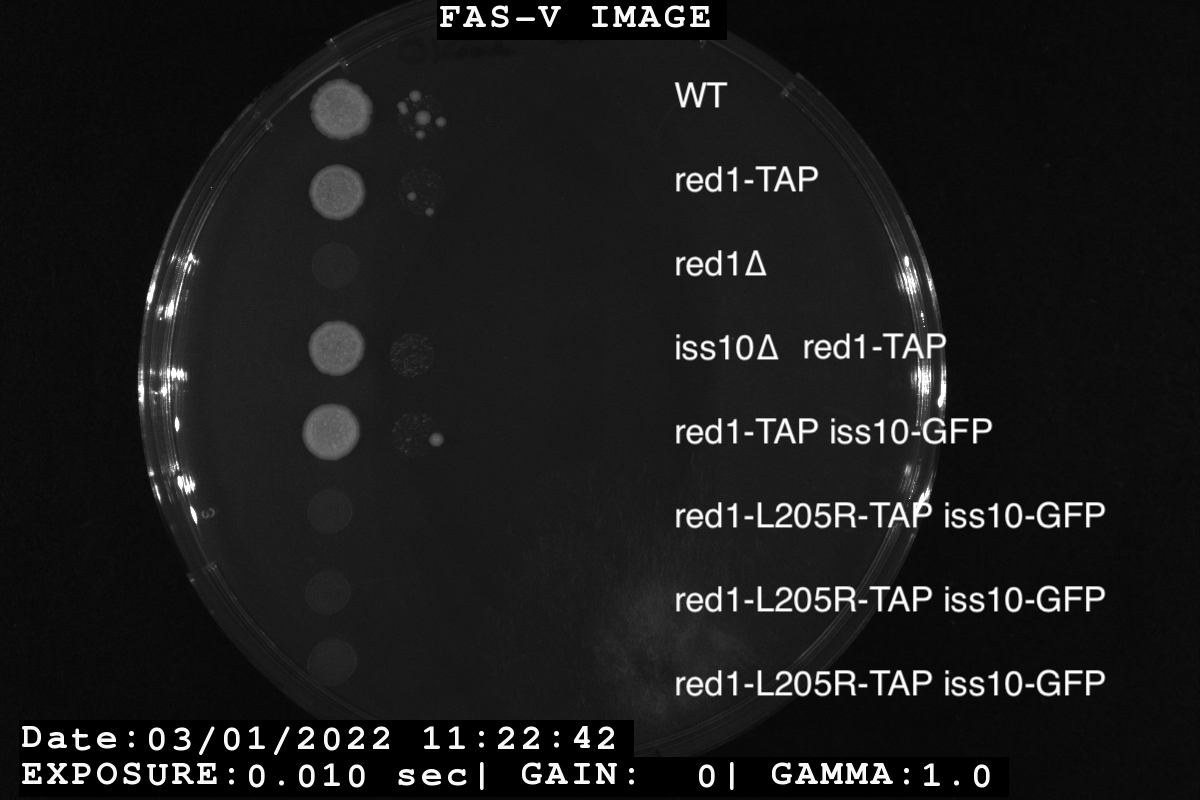

Supplement: Supplementary file 4 — Source Data [file 41467_2022_32542_MOESM4_ESM.zip › Source_data/Figure3&SupFigure3/Sup3c/SupFig3c_MM_18.jpeg]

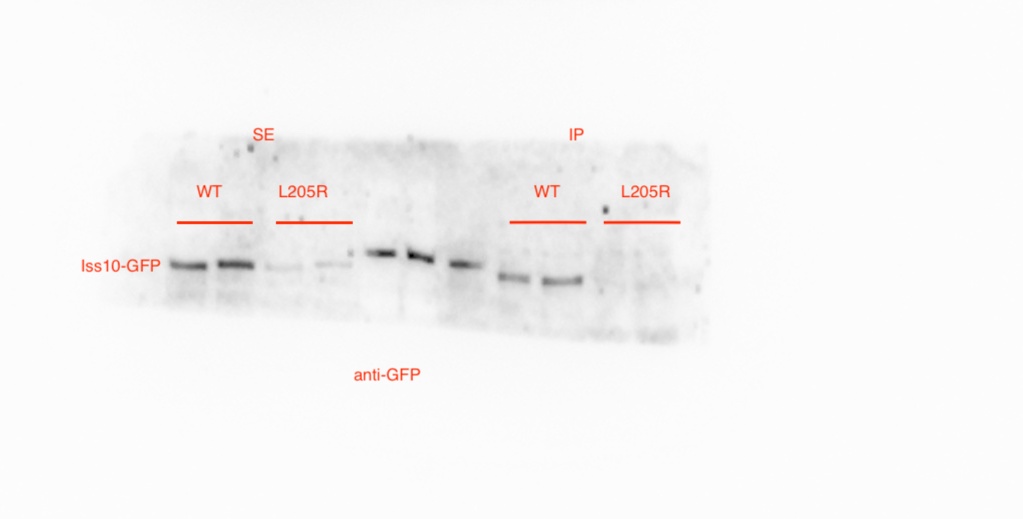

Supplement: Supplementary file 4 — Source Data [file 41467_2022_32542_MOESM4_ESM.zip › Source_data/Figure3&SupFigure3/3a/Fig3a_GFP.JPG]

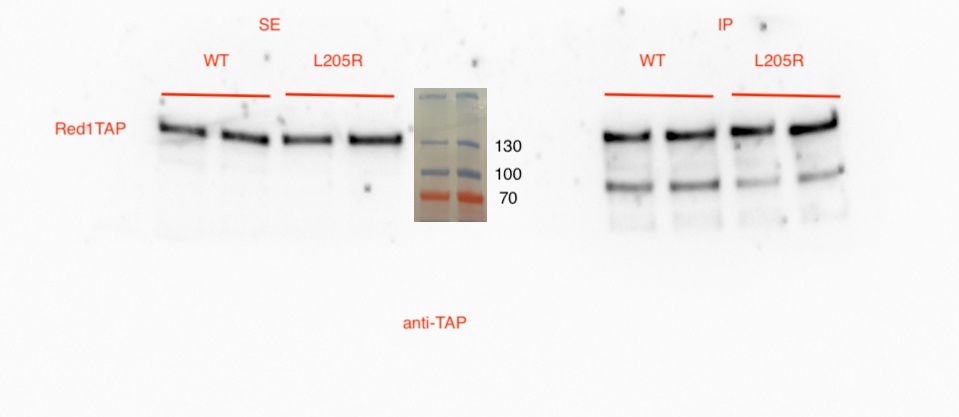

Supplement: Supplementary file 4 — Source Data [file 41467_2022_32542_MOESM4_ESM.zip › Source_data/Figure3&SupFigure3/3a/Fig3a_TAP_WithMolecularWeight.jpg]

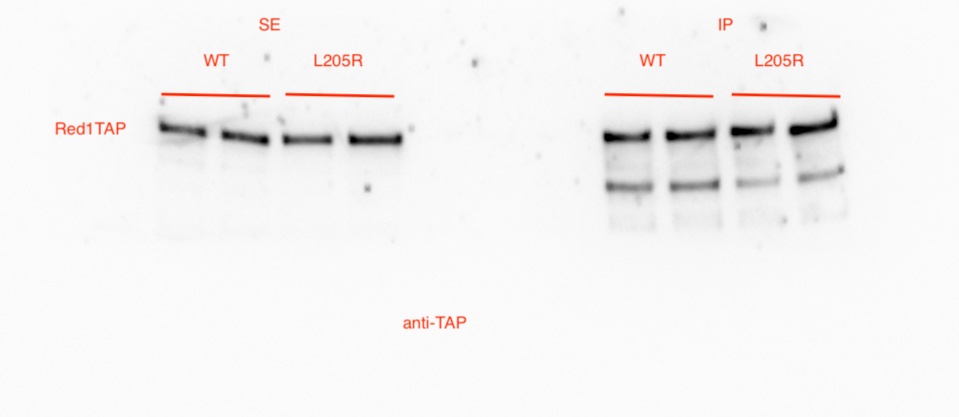

Supplement: Supplementary file 4 — Source Data [file 41467_2022_32542_MOESM4_ESM.zip › Source_data/Figure3&SupFigure3/3a/Fig3a_TAP.JPG]

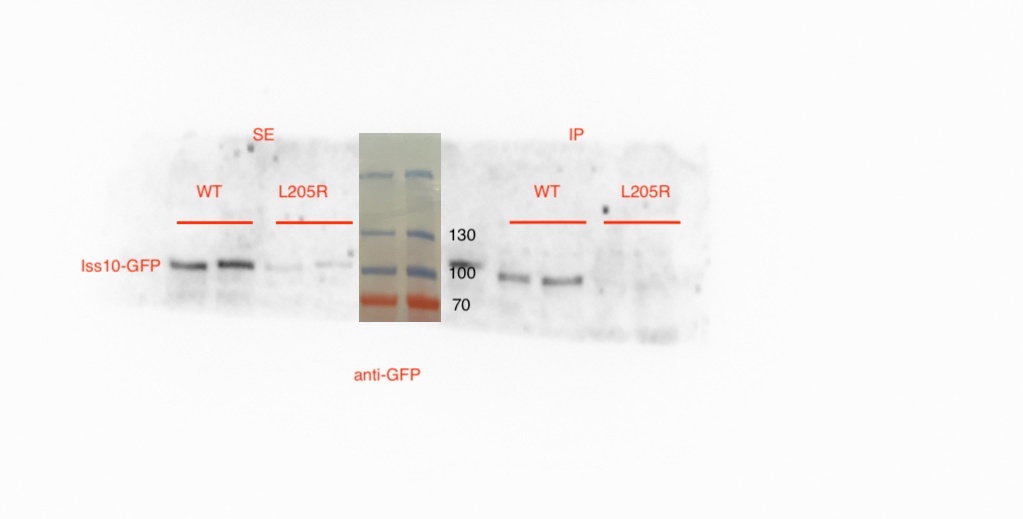

Supplement: Supplementary file 4 — Source Data [file 41467_2022_32542_MOESM4_ESM.zip › Source_data/Figure3&SupFigure3/3a/Fig3a_GFP_WithMolecularWeight.JPG]

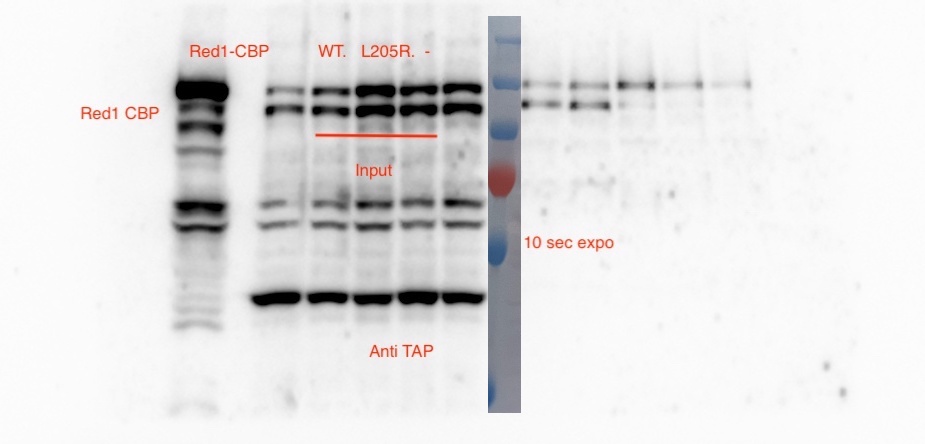

Supplement: Supplementary file 4 — Source Data [file 41467_2022_32542_MOESM4_ESM.zip › Source_data/Figure3&SupFigure3/Sup3a/SupFig3a_TAP_WithMolecularWeight.jpg]

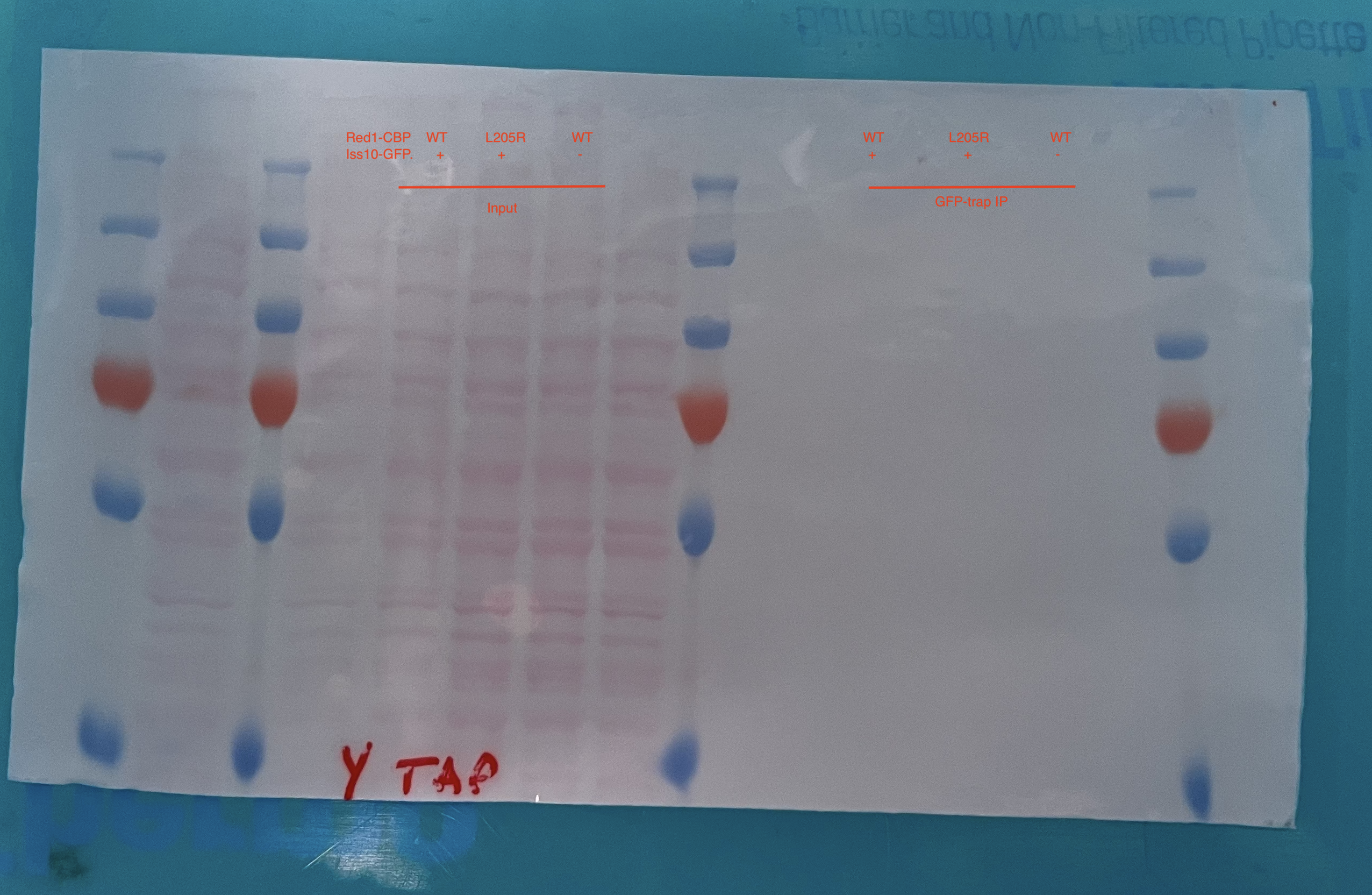

Supplement: Supplementary file 4 — Source Data [file 41467_2022_32542_MOESM4_ESM.zip › Source_data/Figure3&SupFigure3/Sup3a/SupFig3a_Ponceau.tif]

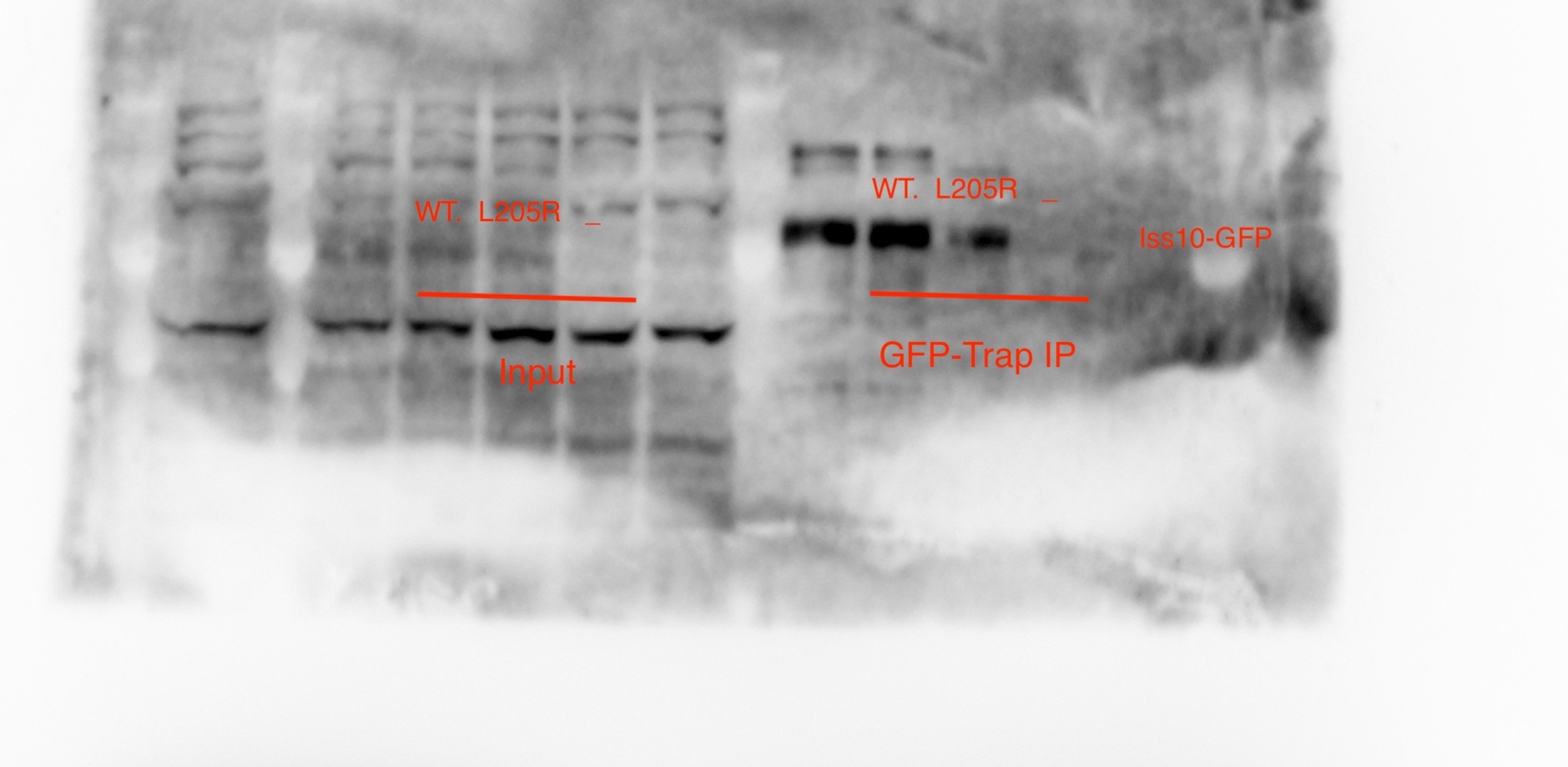

Supplement: Supplementary file 4 — Source Data [file 41467_2022_32542_MOESM4_ESM.zip › Source_data/Figure3&SupFigure3/Sup3a/SupFig3a_GFP.JPG]

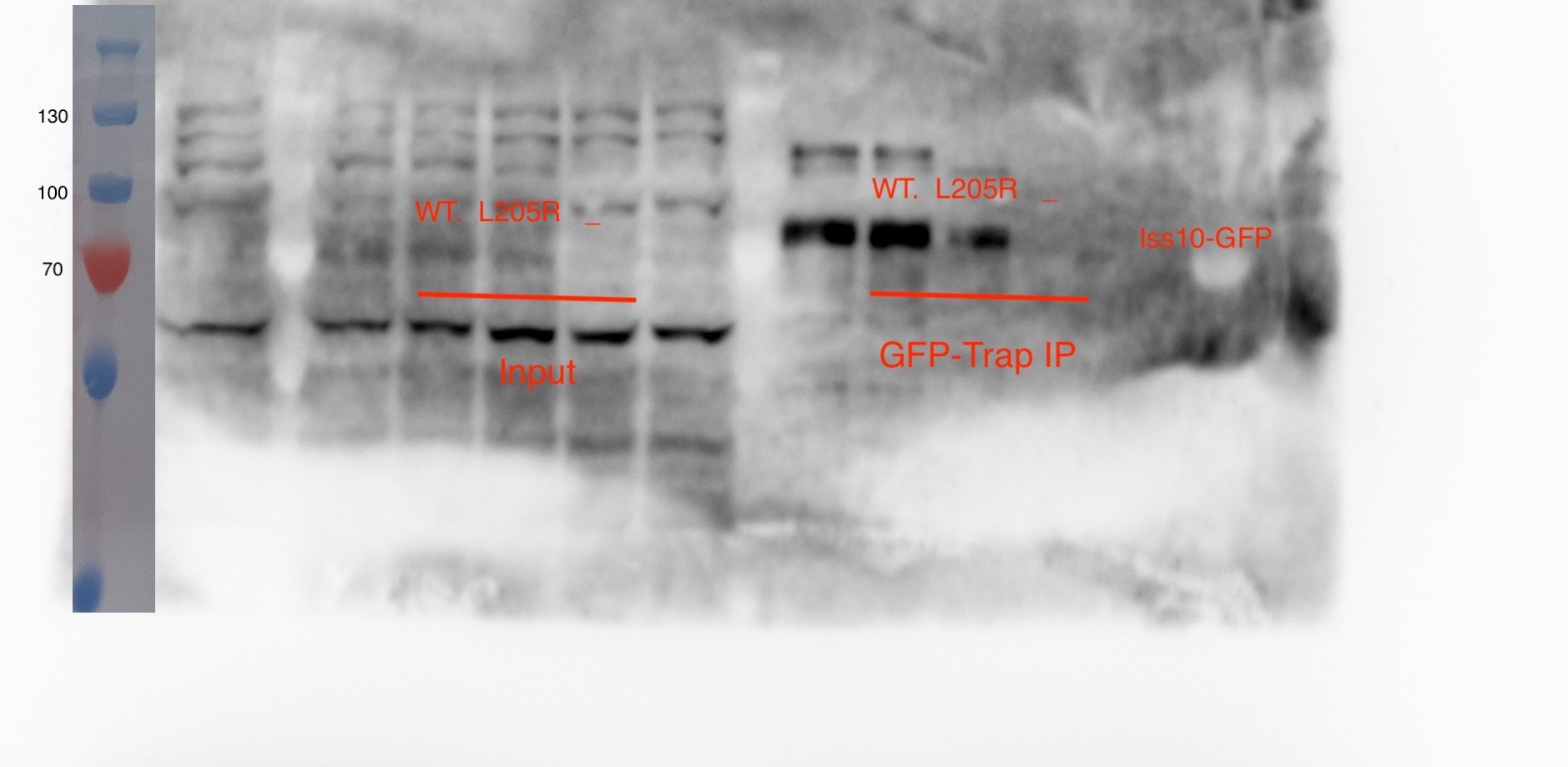

Supplement: Supplementary file 4 — Source Data [file 41467_2022_32542_MOESM4_ESM.zip › Source_data/Figure3&SupFigure3/Sup3a/SupFig3a_GFP_WithMolecularWeight.jpg]

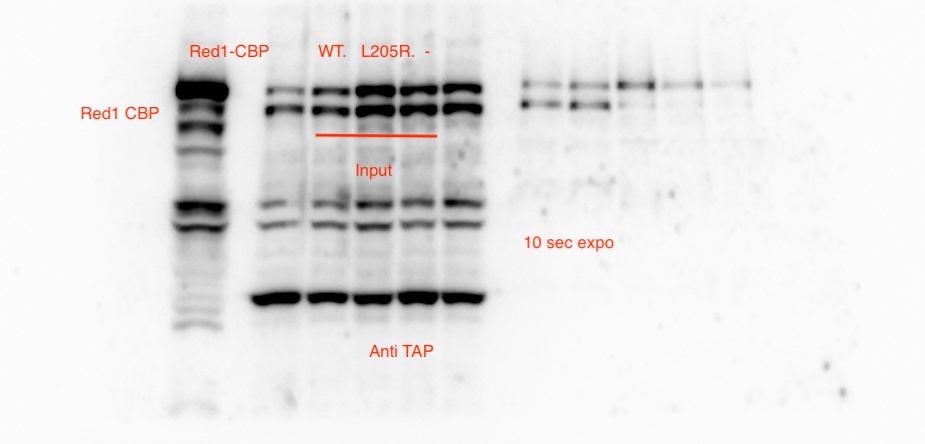

Supplement: Supplementary file 4 — Source Data [file 41467_2022_32542_MOESM4_ESM.zip › Source_data/Figure3&SupFigure3/Sup3a/SupFig3a_TAP.JPG]

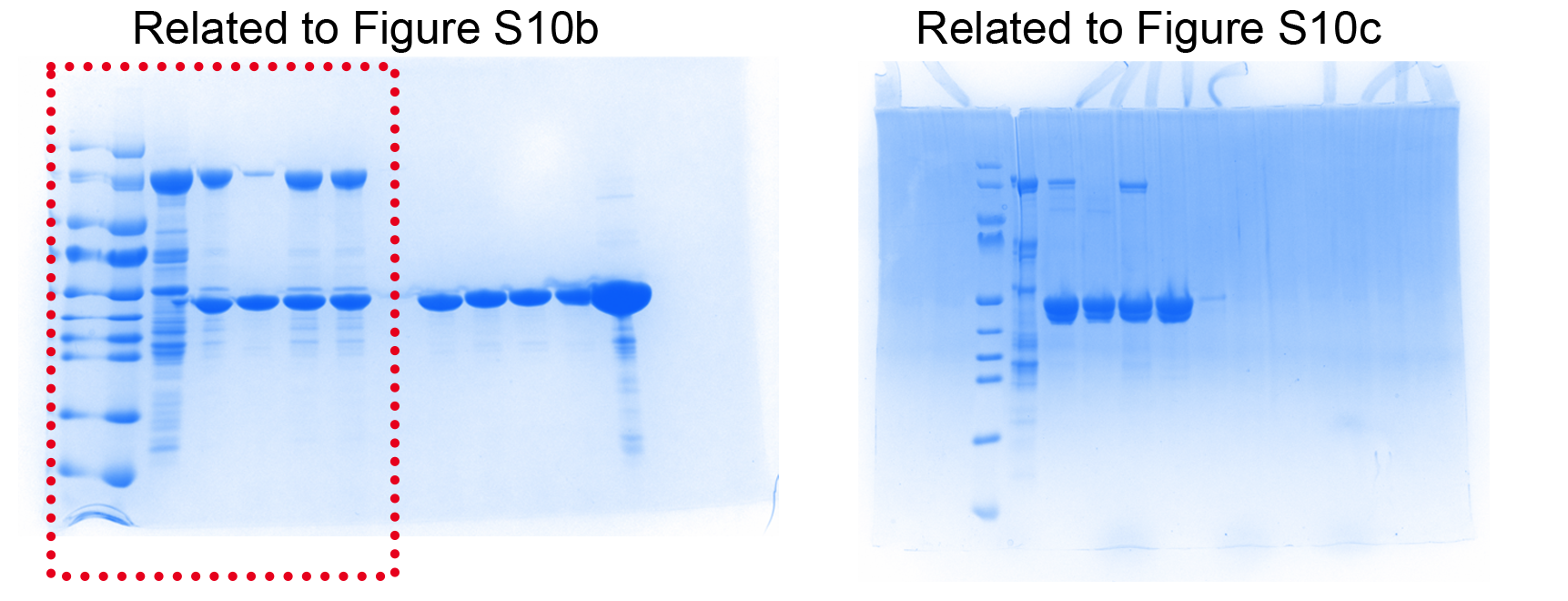

Supplement: Supplementary file 4 — Source Data [file 41467_2022_32542_MOESM4_ESM.zip › Source_data/Figure 6&SupFigure10/Sup10bc/Sup10bc.tif]

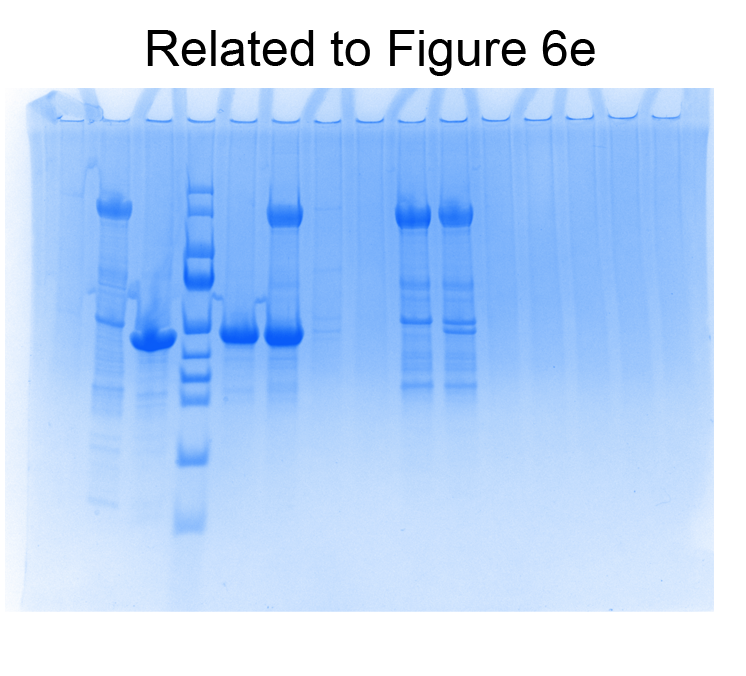

Supplement: Supplementary file 4 — Source Data [file 41467_2022_32542_MOESM4_ESM.zip › Source_data/Figure 6&SupFigure10/6e/6e.tif]

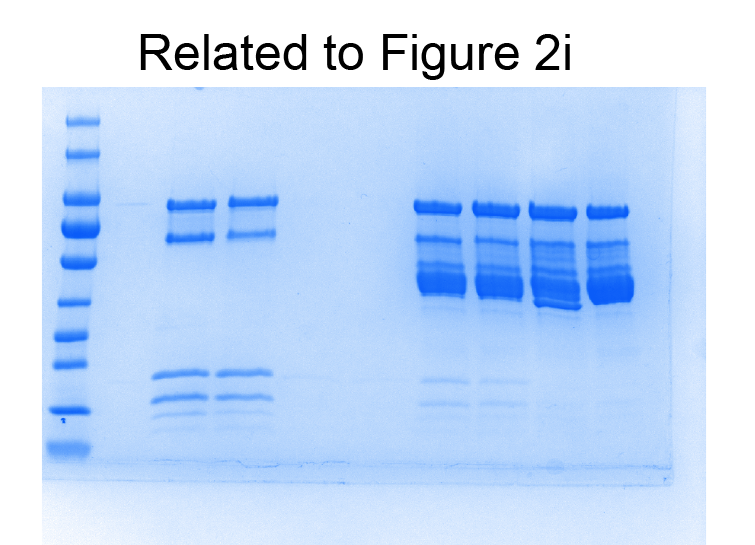

Supplement: Supplementary file 4 — Source Data [file 41467_2022_32542_MOESM4_ESM.zip › Source_data/Figure 2&SupFigure1/2i/Figure_2i.tif]

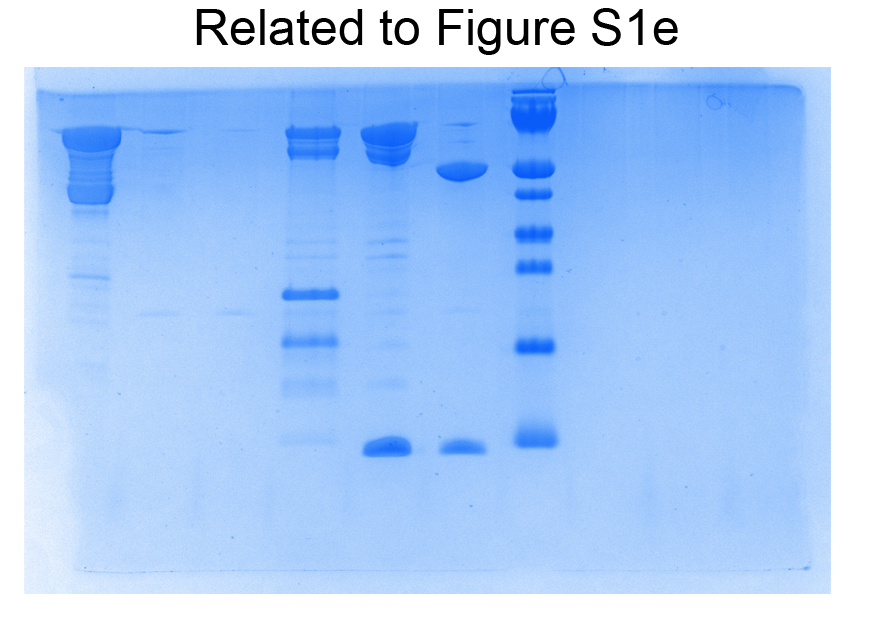

Supplement: Supplementary file 4 — Source Data [file 41467_2022_32542_MOESM4_ESM.zip › Source_data/Figure 2&SupFigure1/Sup1e/Sup1e.tif]

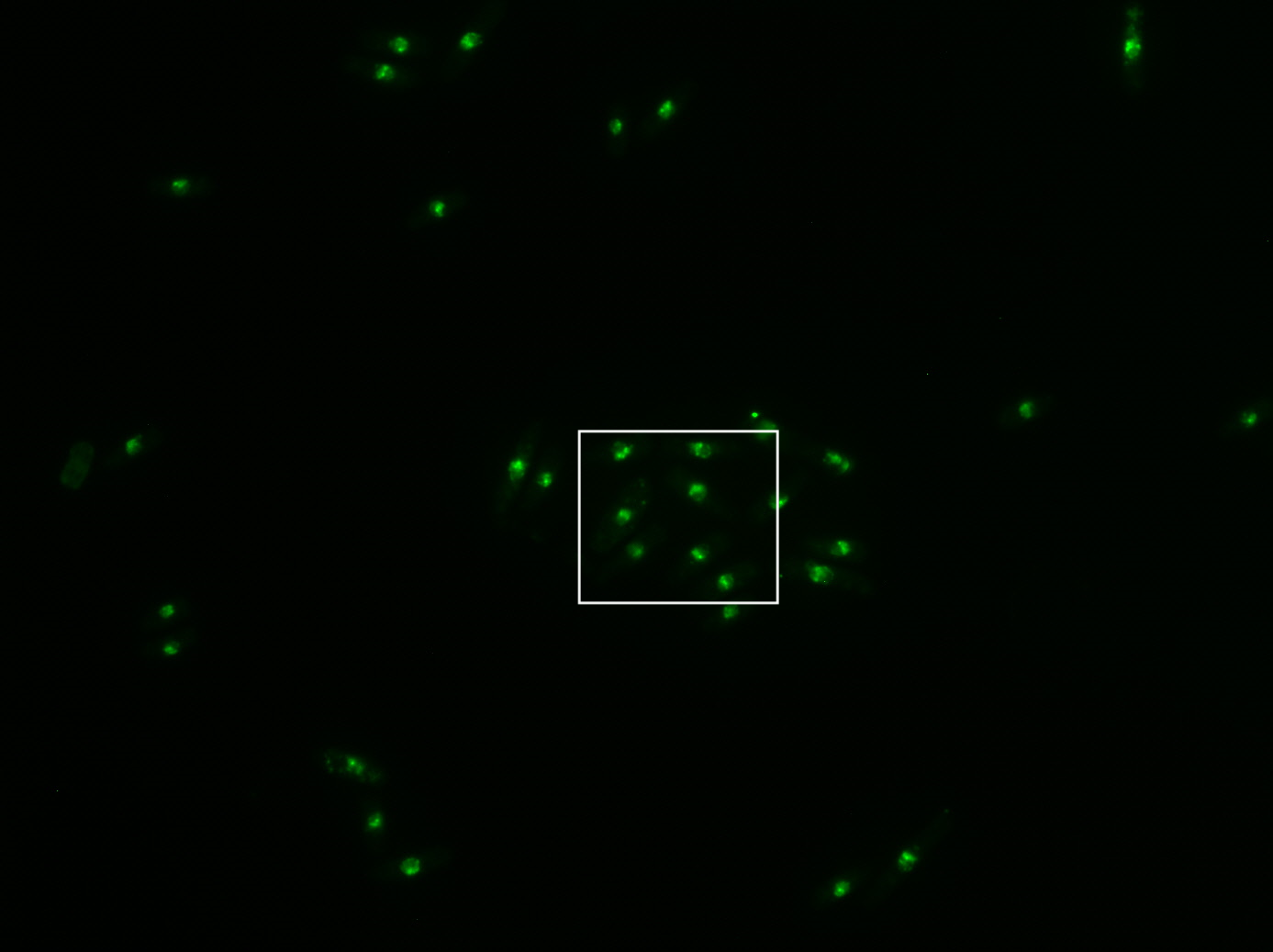

Supplement: Supplementary file 4 — Source Data [file 41467_2022_32542_MOESM4_ESM.zip › Source_data/Figure5&SupFigure8/5i/Fig5i_WT.tif]

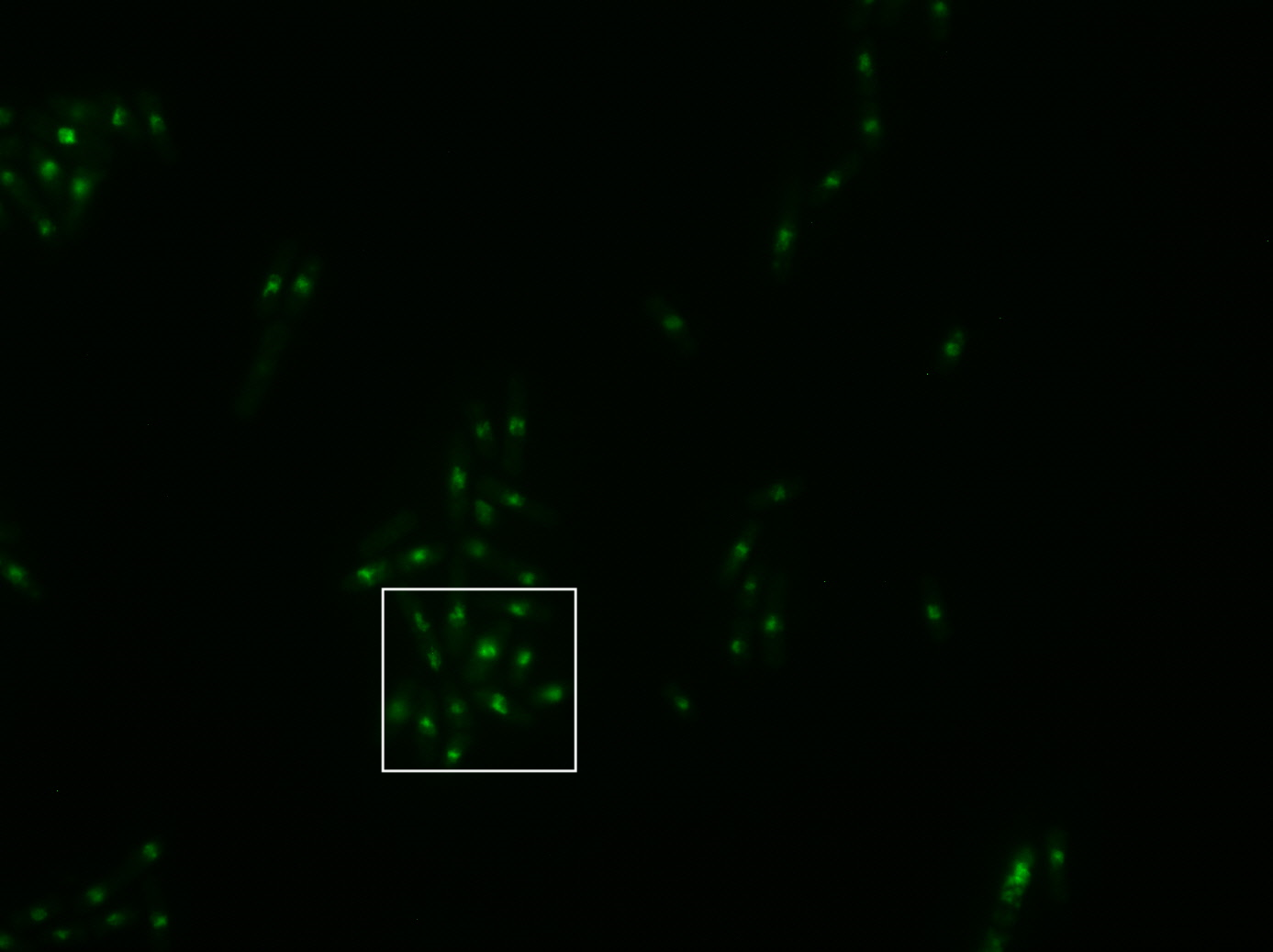

Supplement: Supplementary file 4 — Source Data [file 41467_2022_32542_MOESM4_ESM.zip › Source_data/Figure5&SupFigure8/5i/Fig5i_E32R.tif]

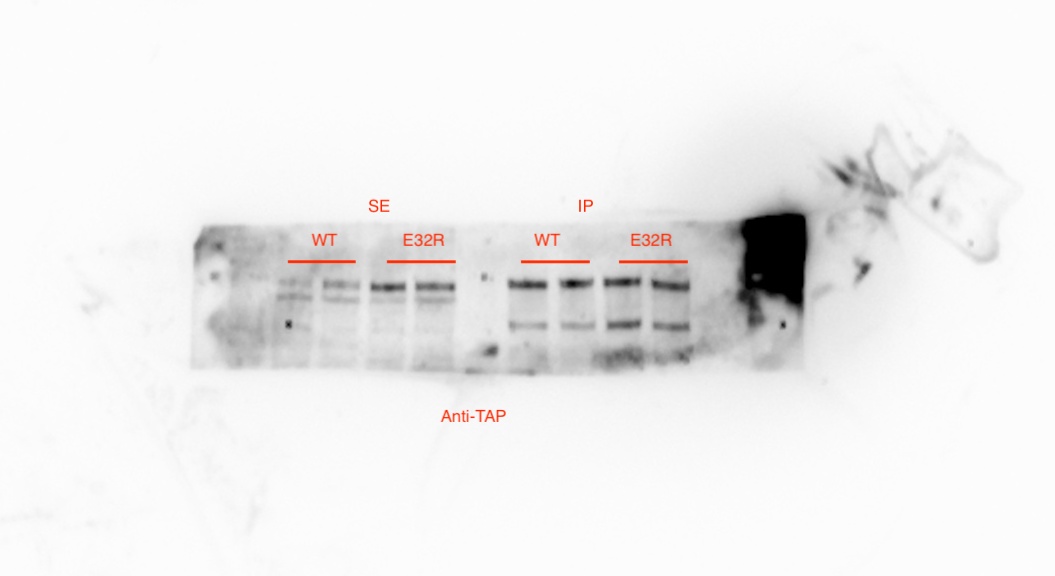

Supplement: Supplementary file 4 — Source Data [file 41467_2022_32542_MOESM4_ESM.zip › Source_data/Figure5&SupFigure8/5g/5g-TAP.JPG]

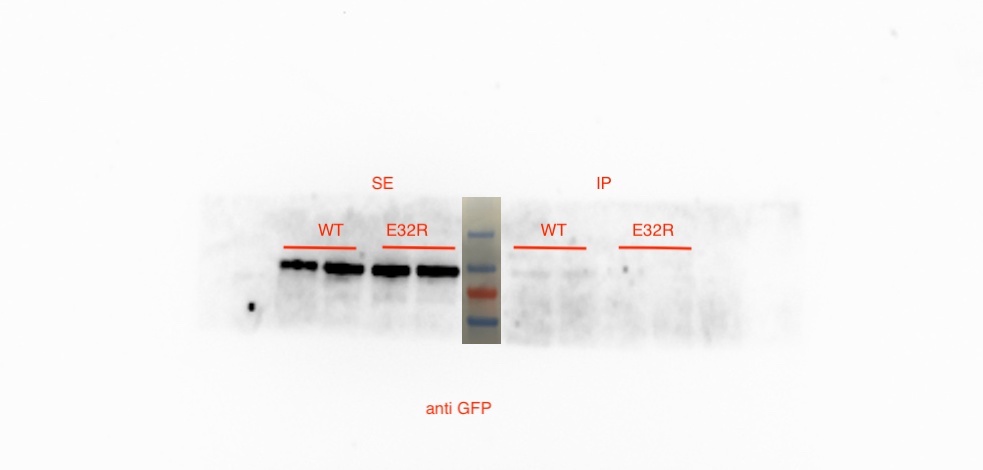

Supplement: Supplementary file 4 — Source Data [file 41467_2022_32542_MOESM4_ESM.zip › Source_data/Figure5&SupFigure8/5g/Fig5g_GFP_WithMolecularWeight.jpg]

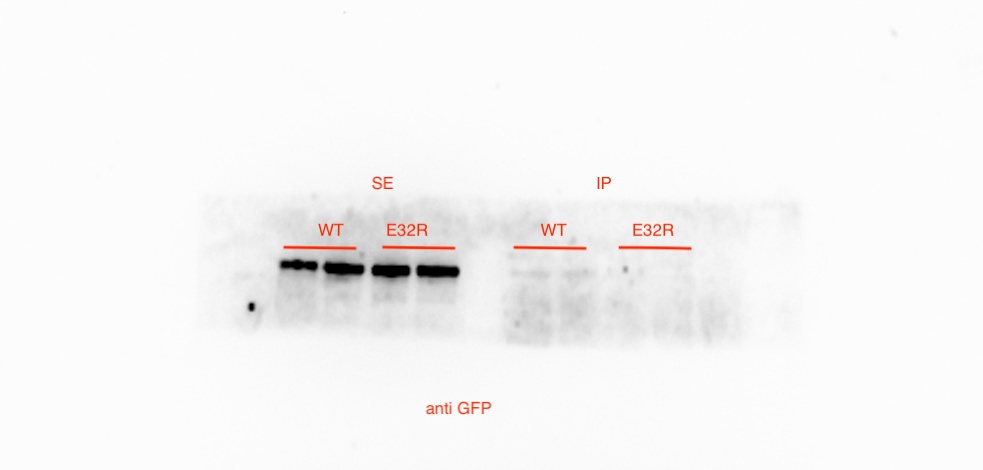

Supplement: Supplementary file 4 — Source Data [file 41467_2022_32542_MOESM4_ESM.zip › Source_data/Figure5&SupFigure8/5g/5g-GFP.jpeg]

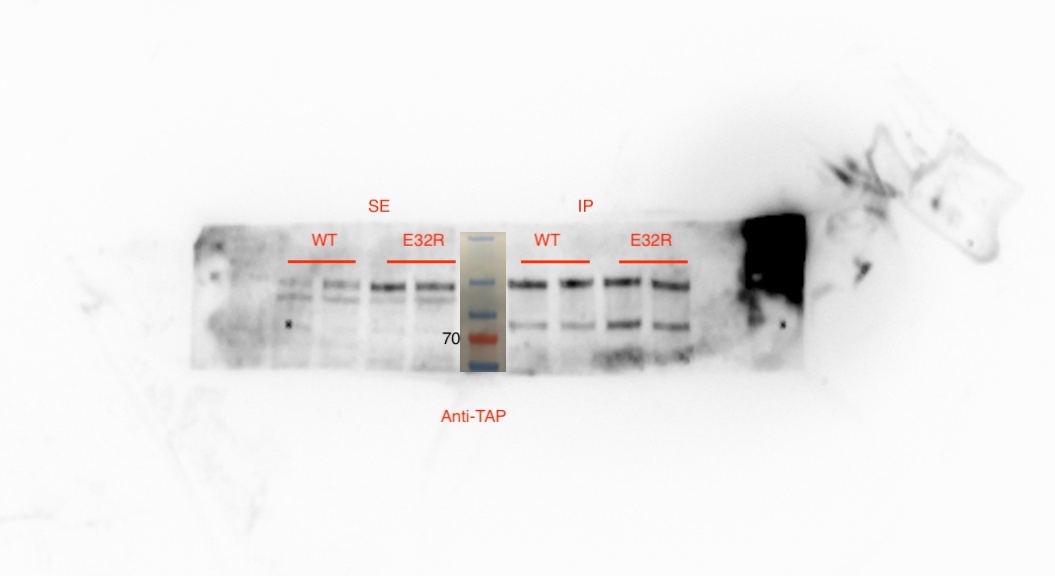

Supplement: Supplementary file 4 — Source Data [file 41467_2022_32542_MOESM4_ESM.zip › Source_data/Figure5&SupFigure8/5g/Fig5g_TAP_WithMolecularWeight.jpg]

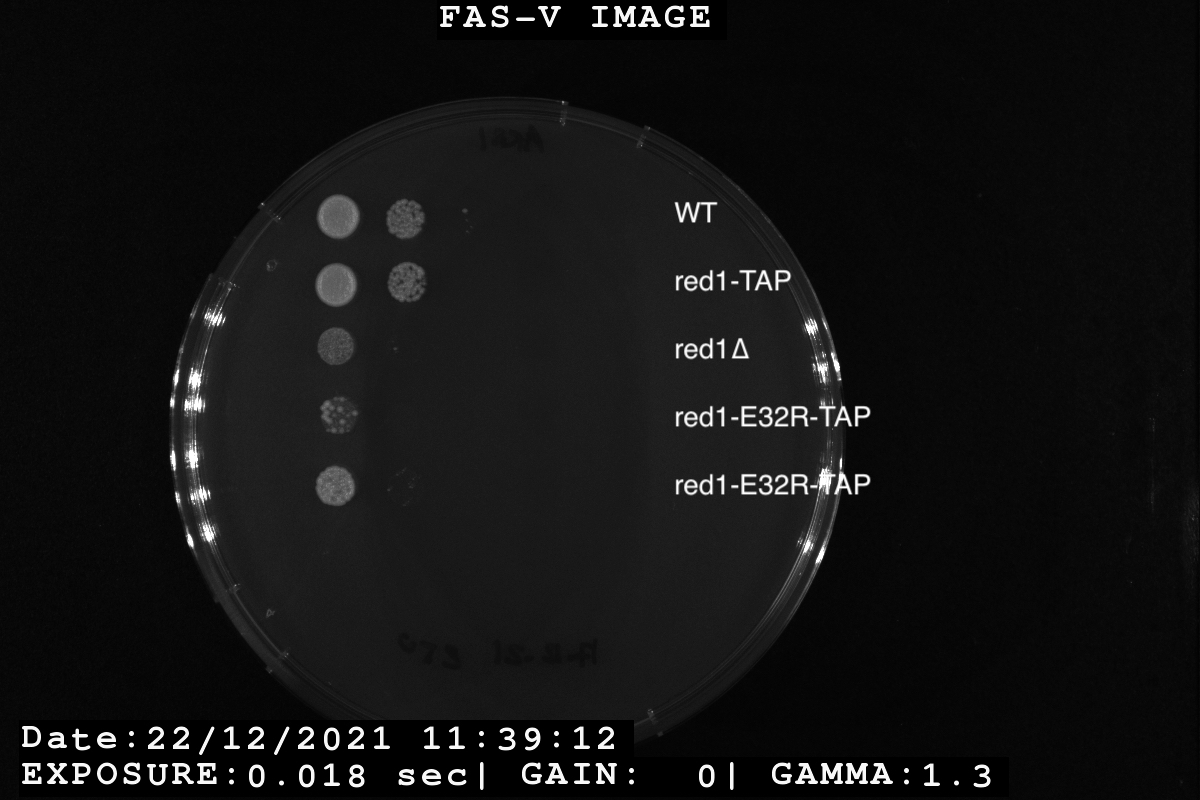

Supplement: Supplementary file 4 — Source Data [file 41467_2022_32542_MOESM4_ESM.zip › Source_data/Figure5&SupFigure8/5h/Fig5h_MM_25.jpeg]

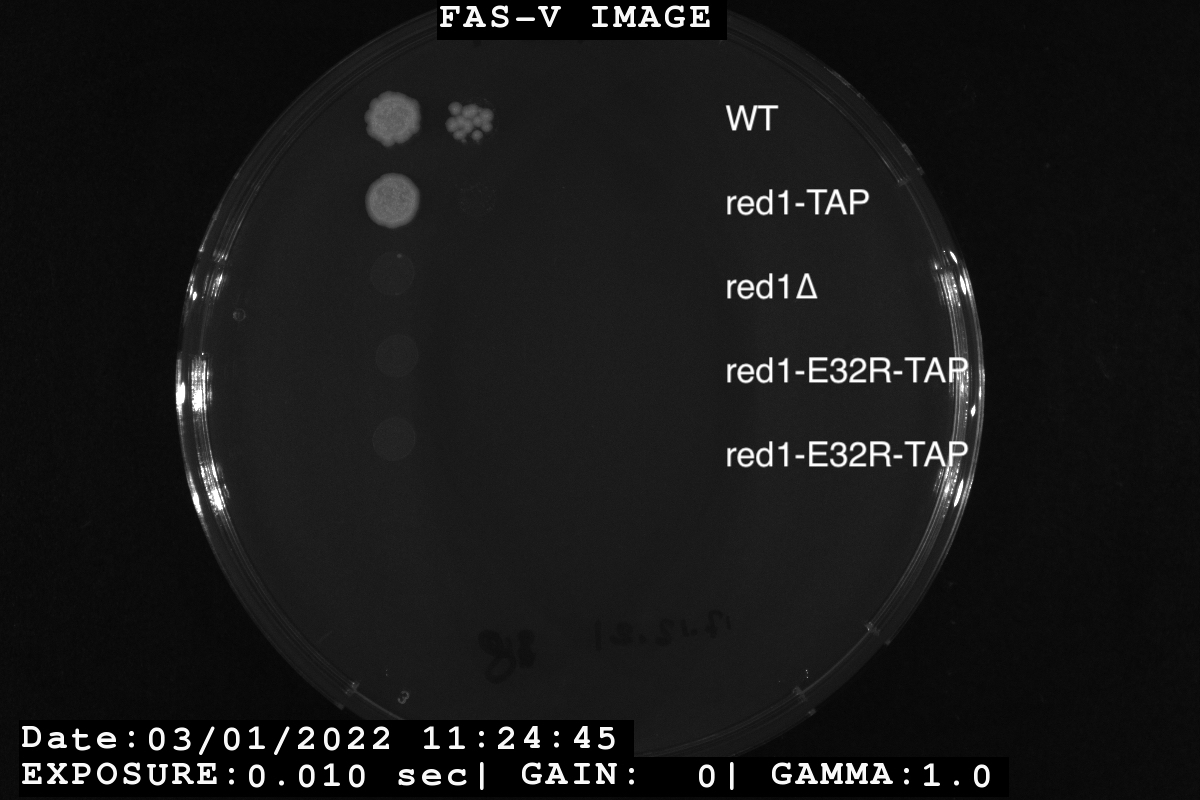

Supplement: Supplementary file 4 — Source Data [file 41467_2022_32542_MOESM4_ESM.zip › Source_data/Figure5&SupFigure8/5h/Fig5h_MM_18.jpeg]

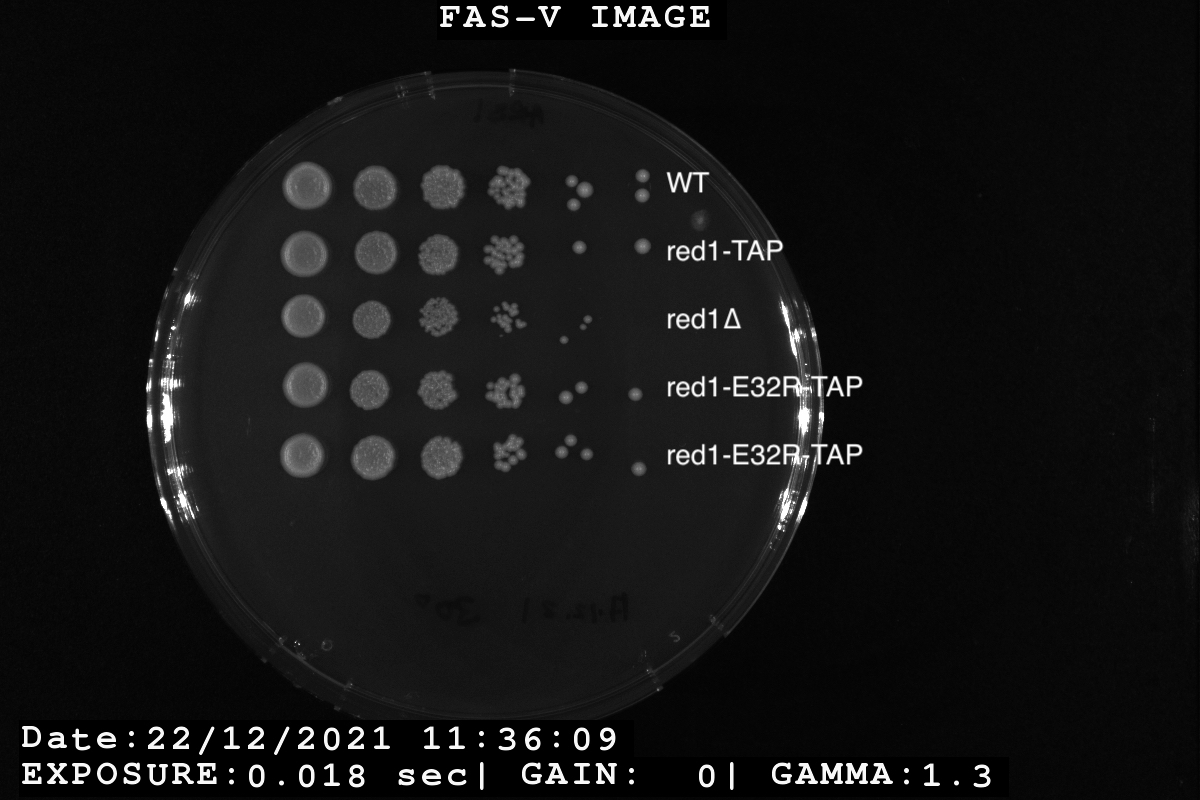

Supplement: Supplementary file 4 — Source Data [file 41467_2022_32542_MOESM4_ESM.zip › Source_data/Figure5&SupFigure8/5h/Fig5h_RM_30.jpeg]

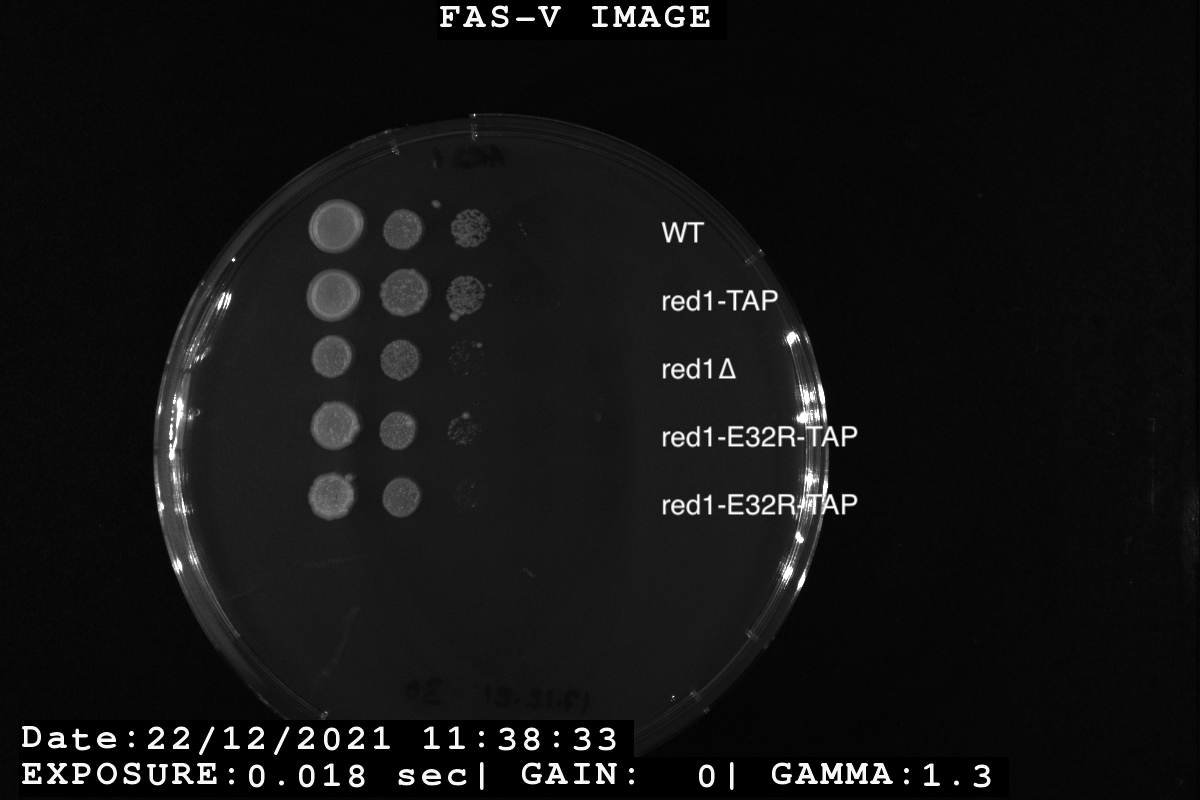

Supplement: Supplementary file 4 — Source Data [file 41467_2022_32542_MOESM4_ESM.zip › Source_data/Figure5&SupFigure8/5h/Fig5h_MM_30.jpeg]

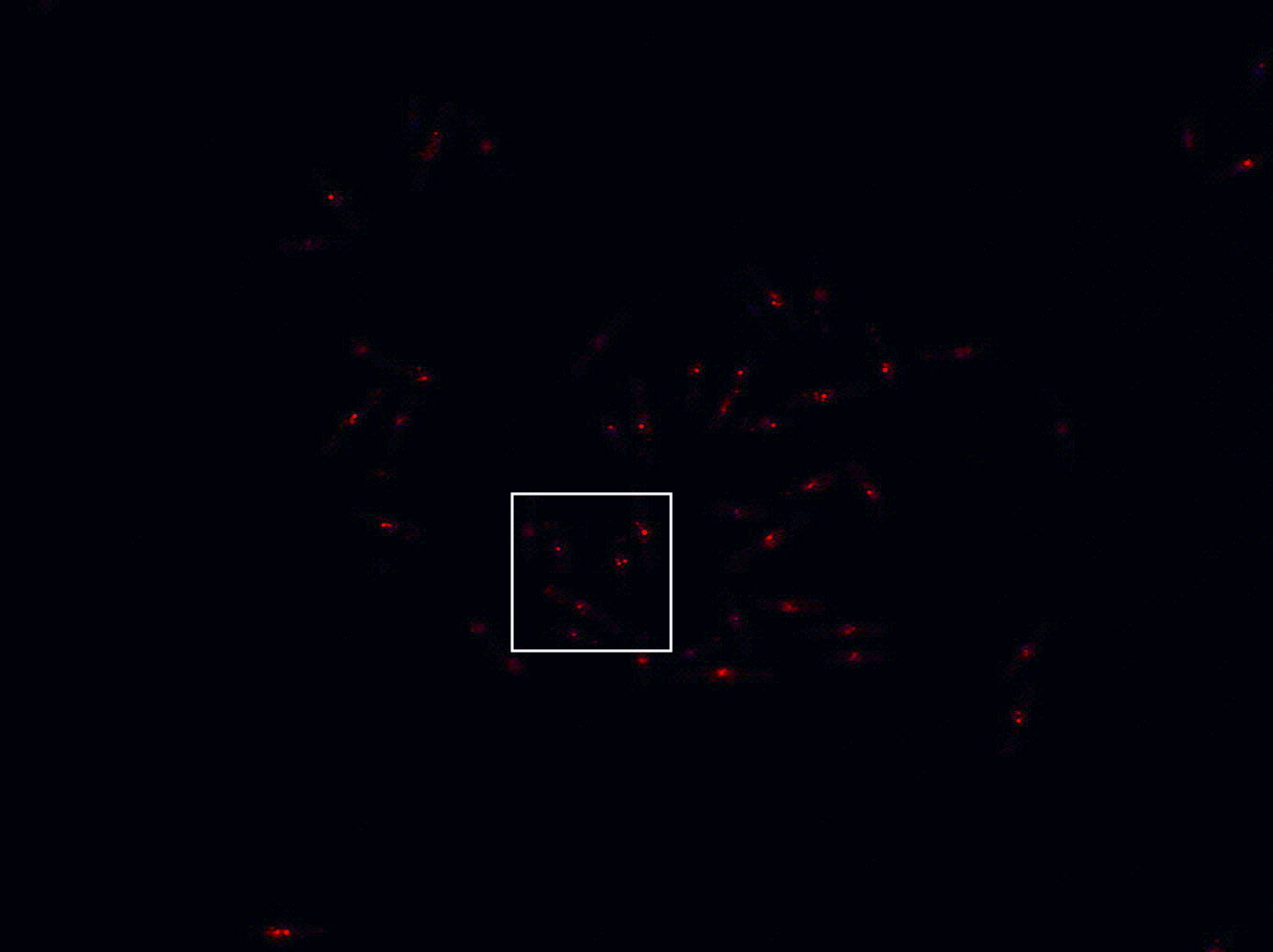

Supplement: Supplementary file 4 — Source Data [file 41467_2022_32542_MOESM4_ESM.zip › Source_data/Figure5&SupFigure8/Sup8f/SupFig8f_WT.JPG]

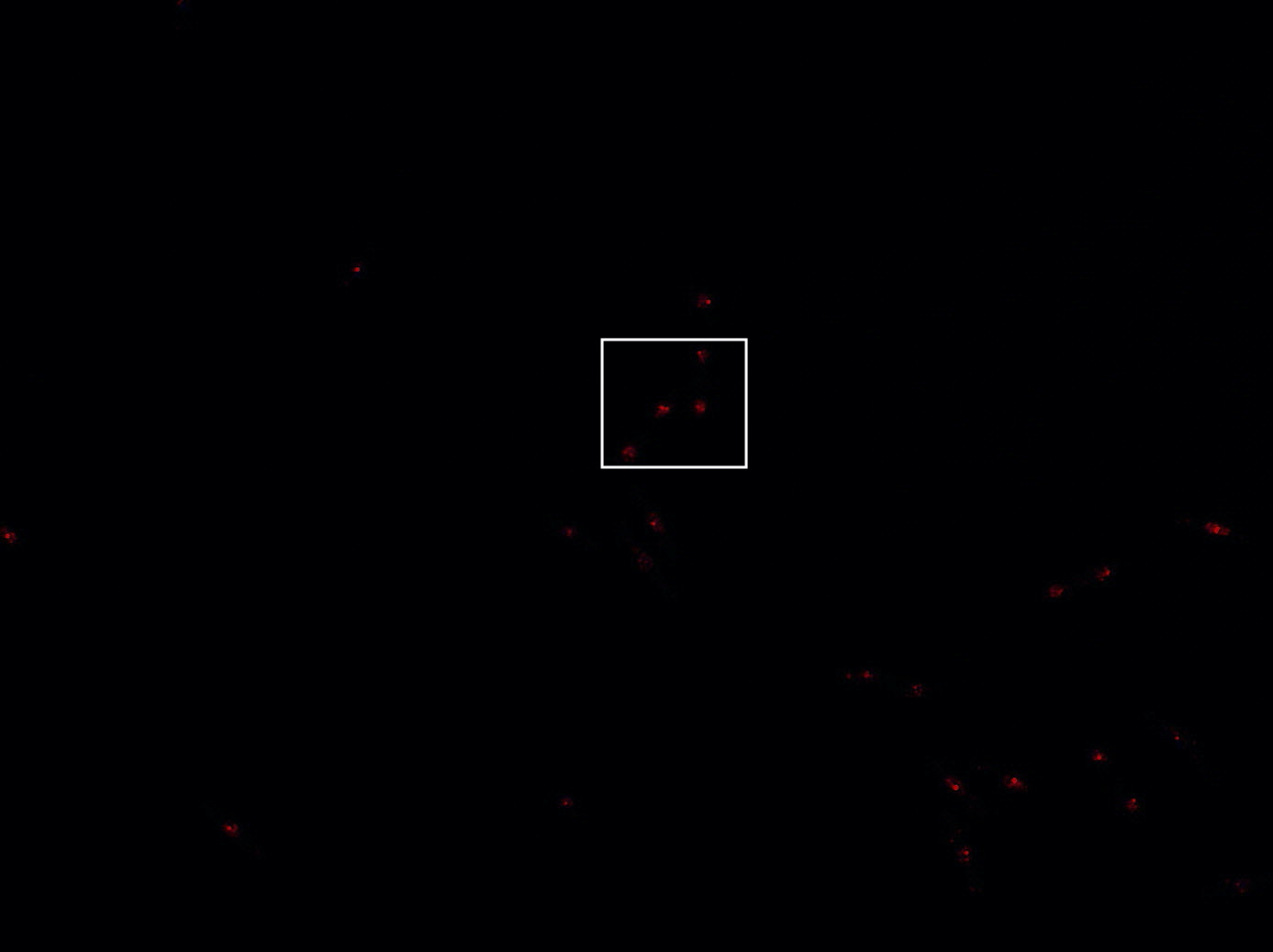

Supplement: Supplementary file 4 — Source Data [file 41467_2022_32542_MOESM4_ESM.zip › Source_data/Figure5&SupFigure8/Sup8f/SupFig8f_E32R.JPG]

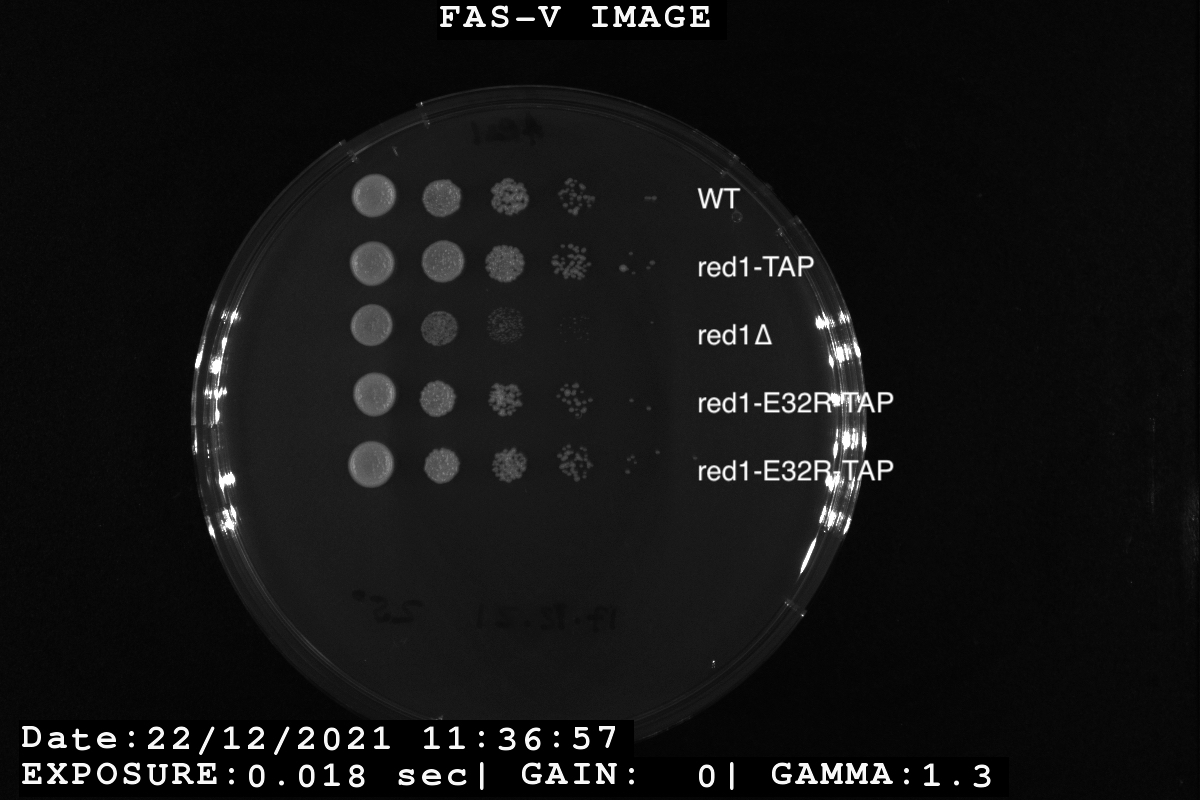

Supplement: Supplementary file 4 — Source Data [file 41467_2022_32542_MOESM4_ESM.zip › Source_data/Figure5&SupFigure8/Sup8a/SupFig8a_RM_25.jpeg]

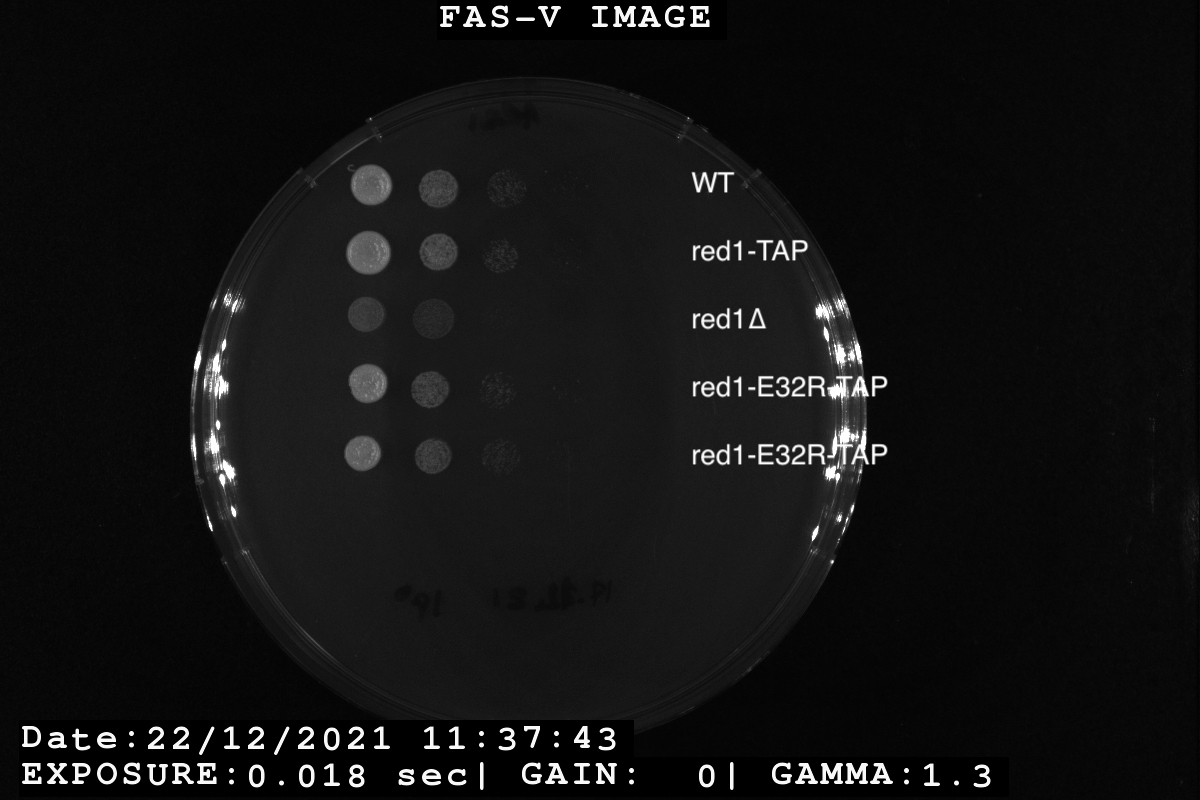

Supplement: Supplementary file 4 — Source Data [file 41467_2022_32542_MOESM4_ESM.zip › Source_data/Figure5&SupFigure8/Sup8a/SupFig8a_RM_18.jpeg]

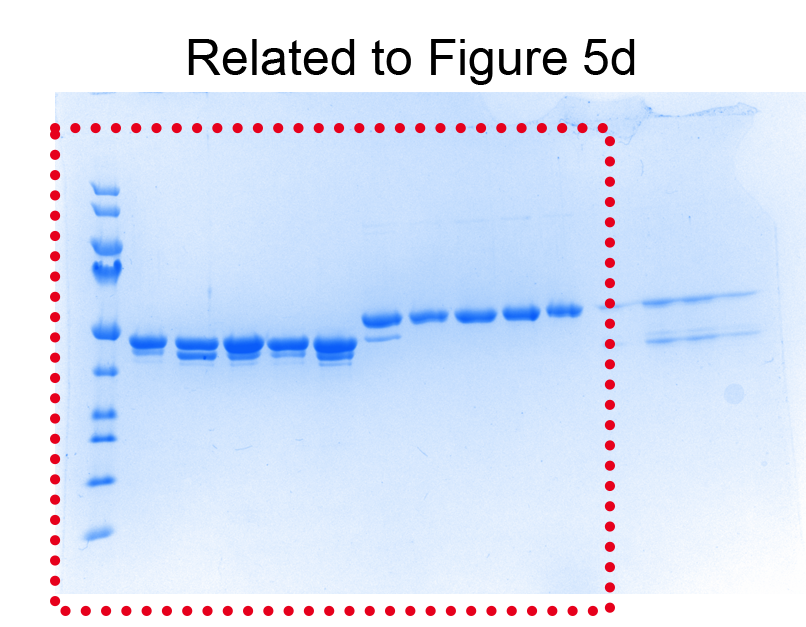

Supplement: Supplementary file 4 — Source Data [file 41467_2022_32542_MOESM4_ESM.zip › Source_data/Figure5&SupFigure8/5d/5d.tif]

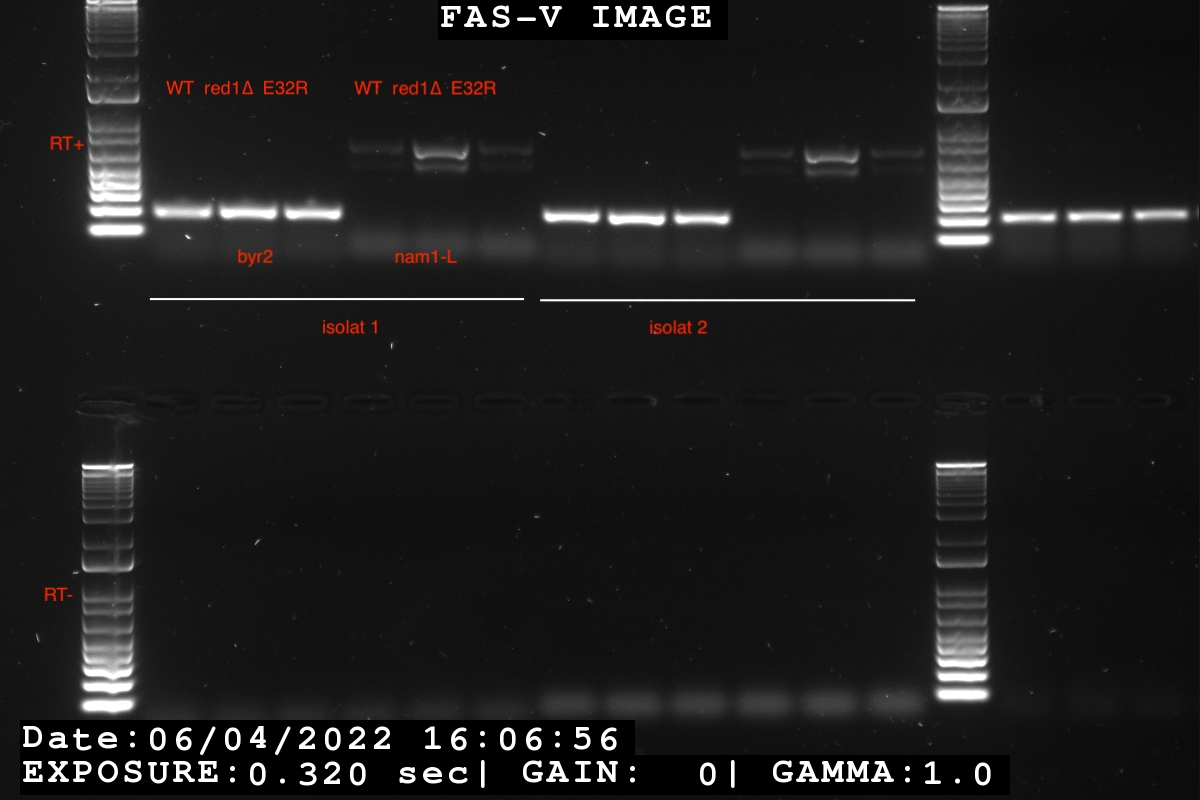

Supplement: Supplementary file 4 — Source Data [file 41467_2022_32542_MOESM4_ESM.zip › Source_data/Figure5&SupFigure8/Sup8d/SupFig8d_BYR2_NAML.jpeg]

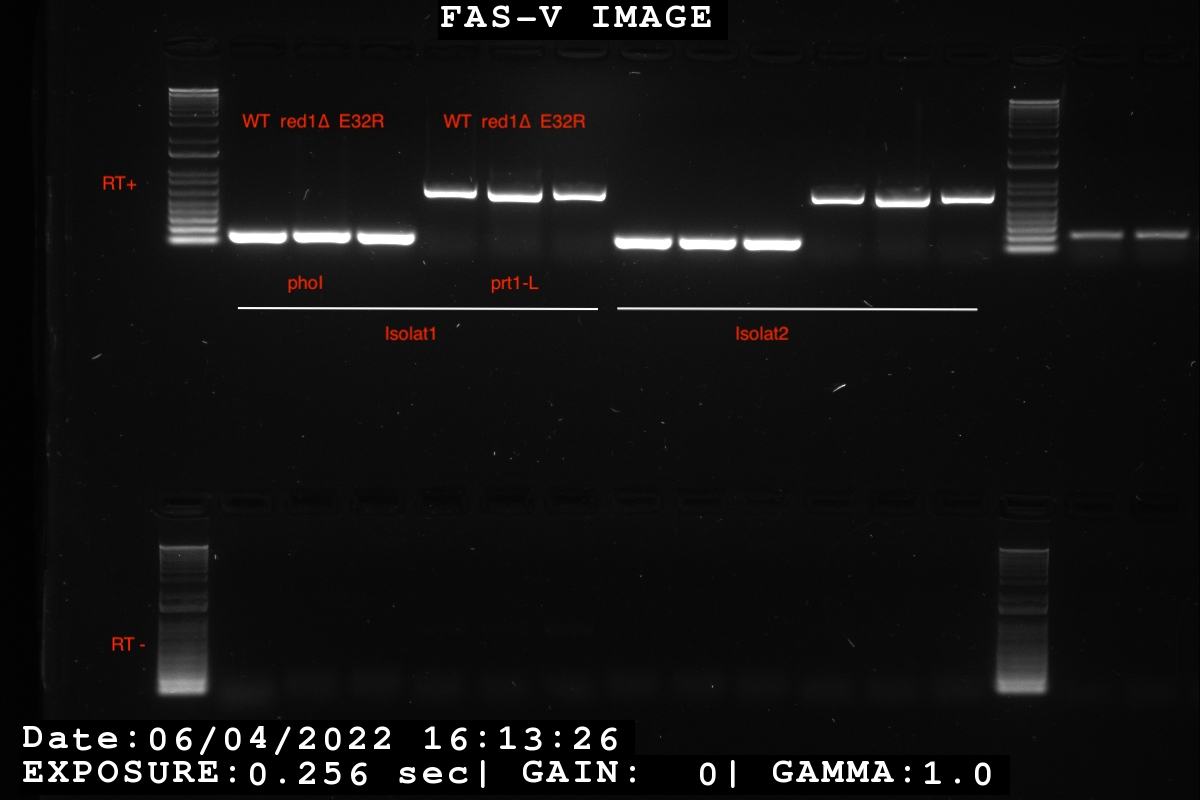

Supplement: Supplementary file 4 — Source Data [file 41467_2022_32542_MOESM4_ESM.zip › Source_data/Figure5&SupFigure8/Sup8d/SupFig8d_PRTL-PHO1.jpeg]

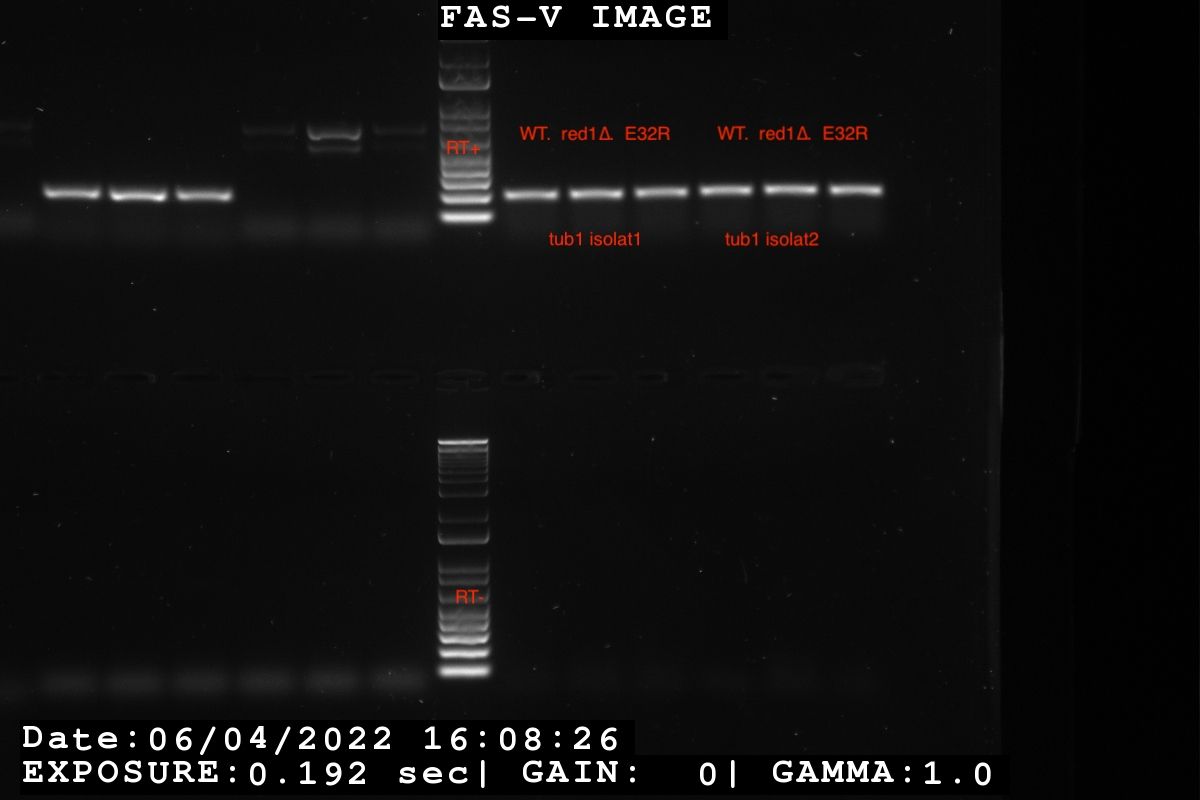

Supplement: Supplementary file 4 — Source Data [file 41467_2022_32542_MOESM4_ESM.zip › Source_data/Figure5&SupFigure8/Sup8d/SupFig8d_TUB1_BYR2.jpeg]

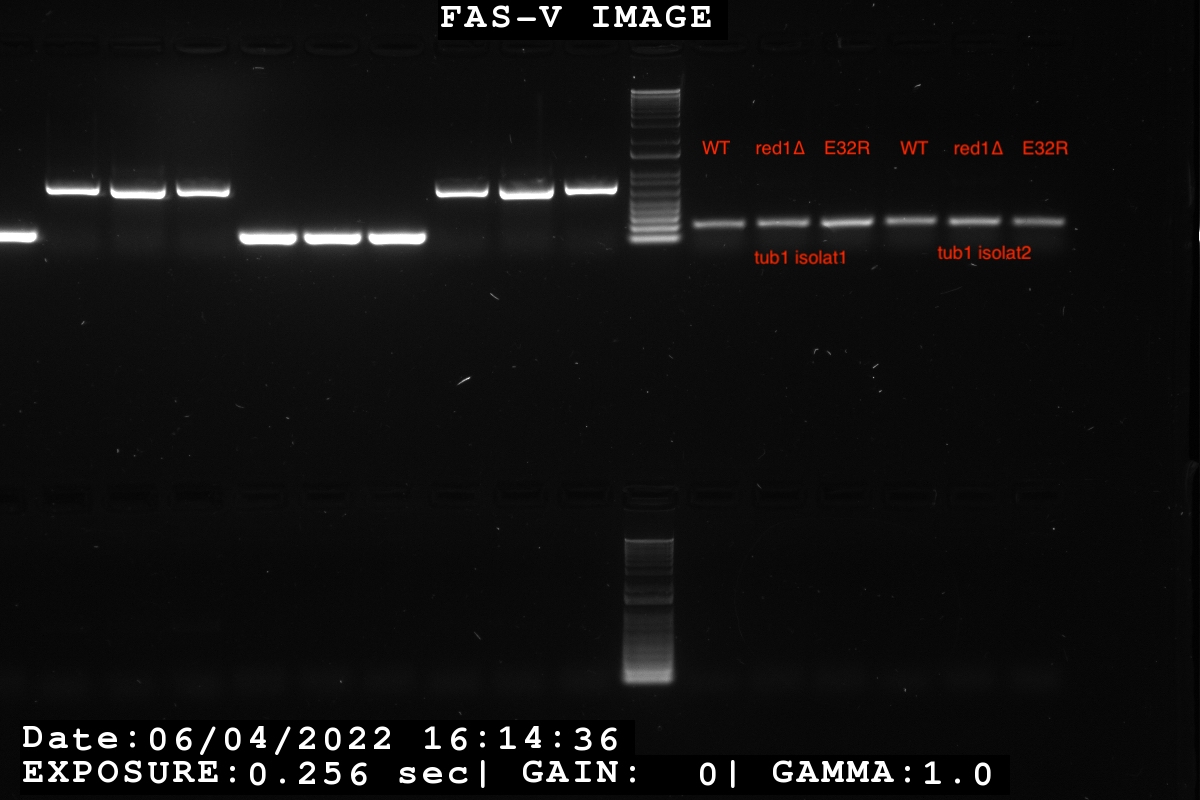

Supplement: Supplementary file 4 — Source Data [file 41467_2022_32542_MOESM4_ESM.zip › Source_data/Figure5&SupFigure8/Sup8d/SupFig8d_PHO1_TUB1.jpeg]

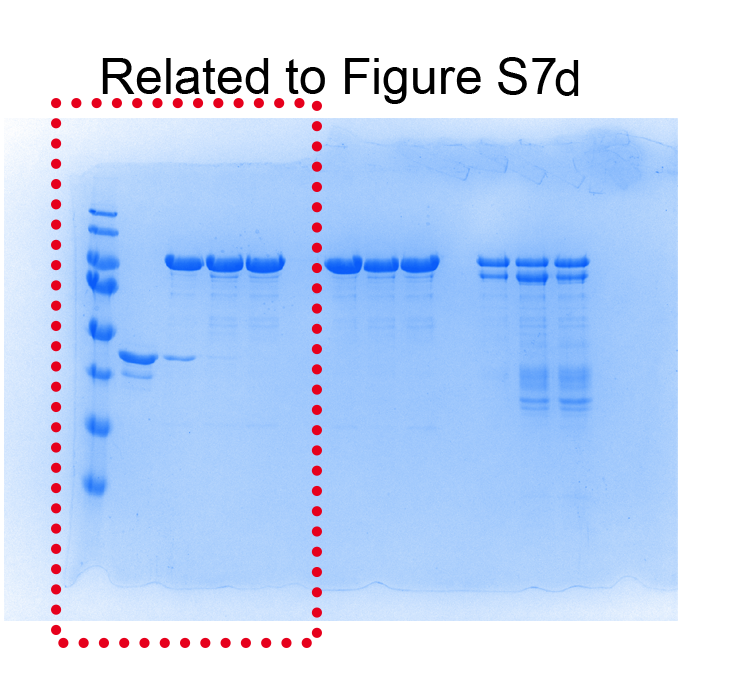

Supplement: Supplementary file 4 — Source Data [file 41467_2022_32542_MOESM4_ESM.zip › Source_data/SupFigure7/Sup7d.tif]

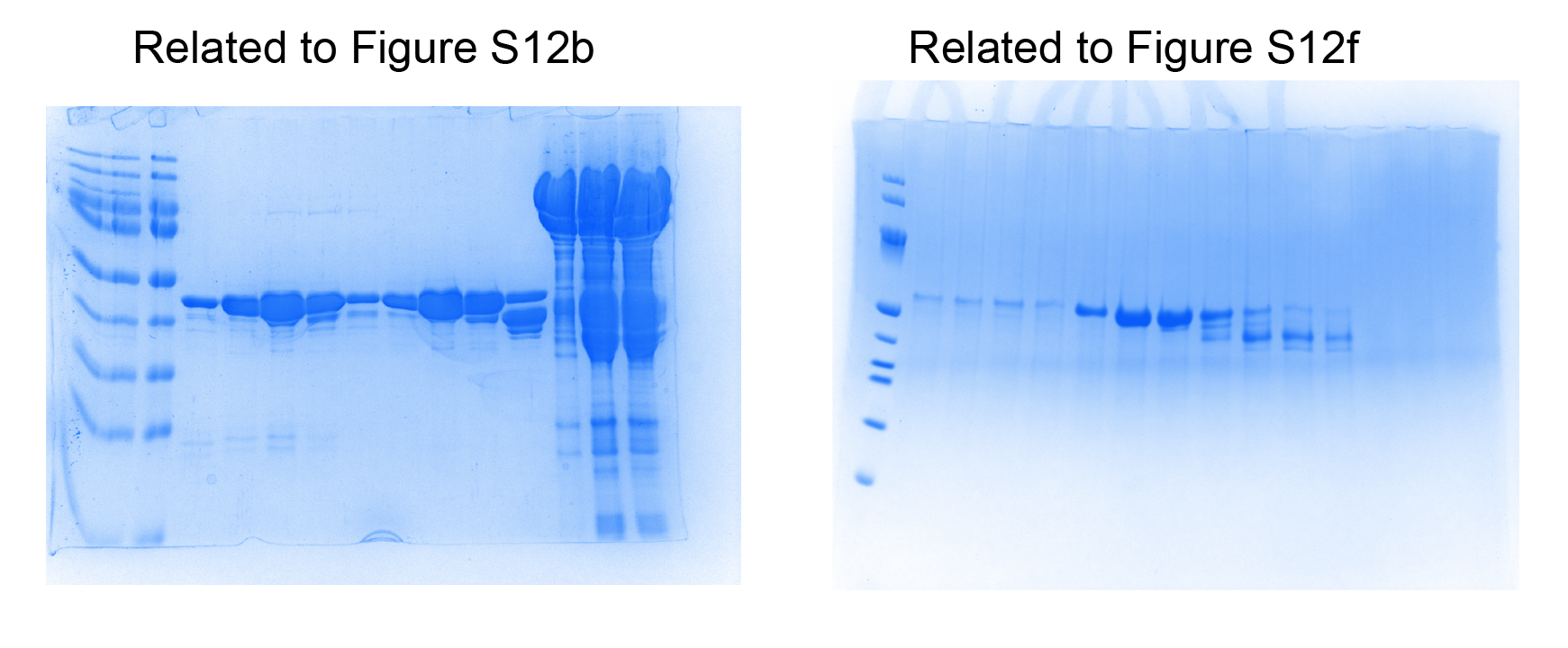

Supplement: Supplementary file 4 — Source Data [file 41467_2022_32542_MOESM4_ESM.zip › Source_data/SupFigure12/Sup12bf.tif]

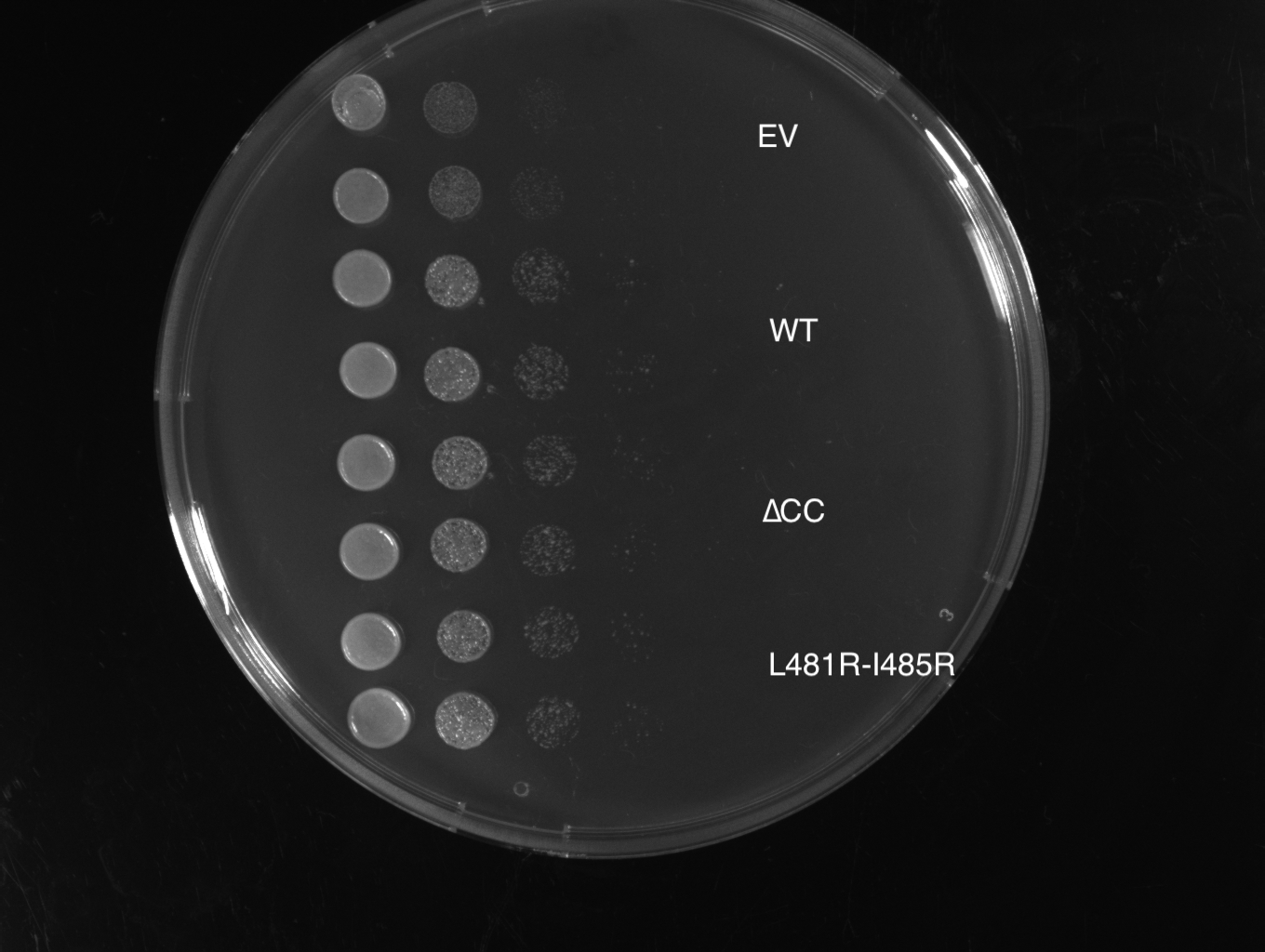

Supplement: Supplementary file 4 — Source Data [file 41467_2022_32542_MOESM4_ESM.zip › Source_data/Figure7&SupFigure13/Sup13c/SupFig13c-25.Tif]

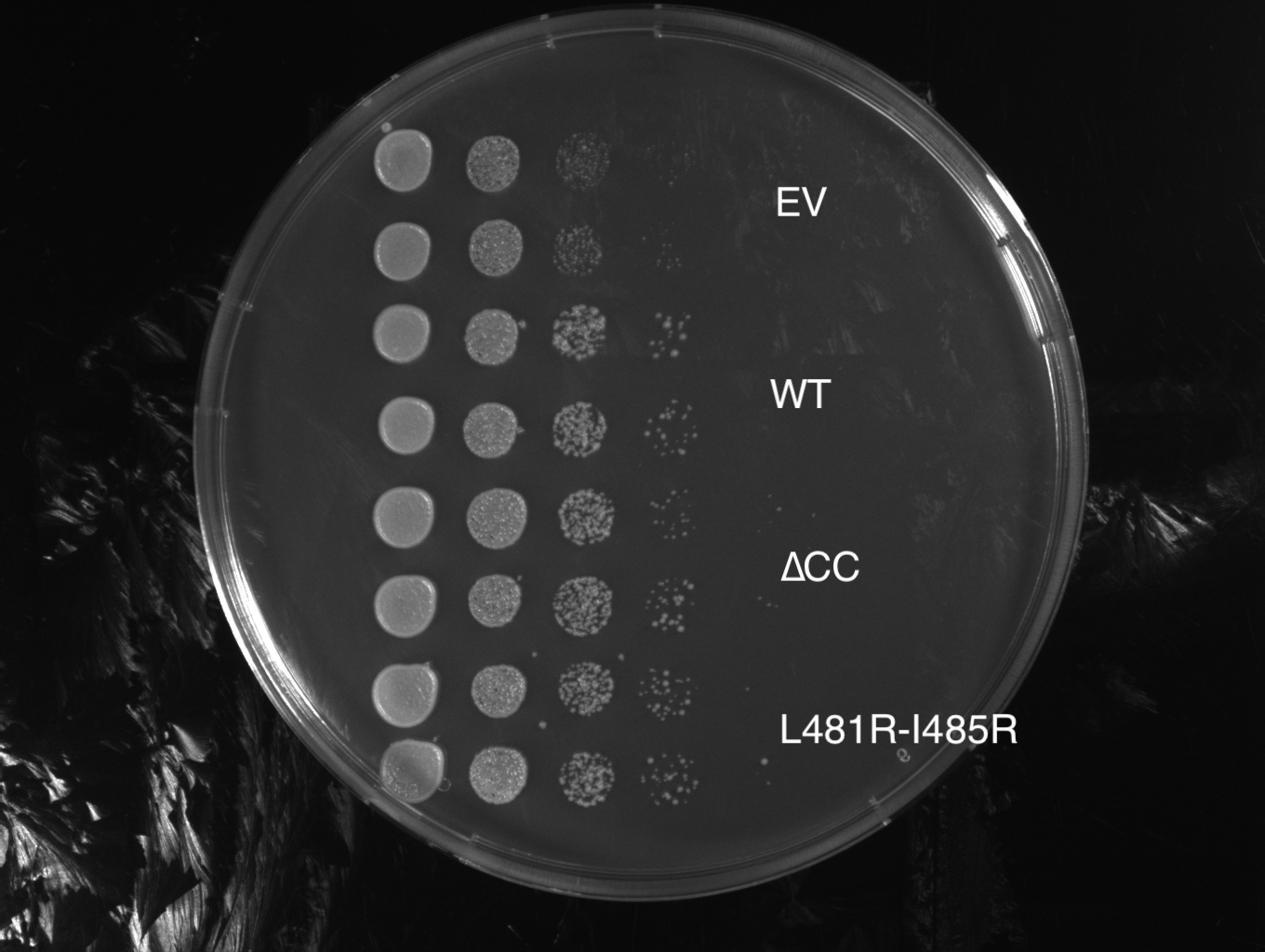

Supplement: Supplementary file 4 — Source Data [file 41467_2022_32542_MOESM4_ESM.zip › Source_data/Figure7&SupFigure13/Sup13c/SupFig13c-30.Tif]

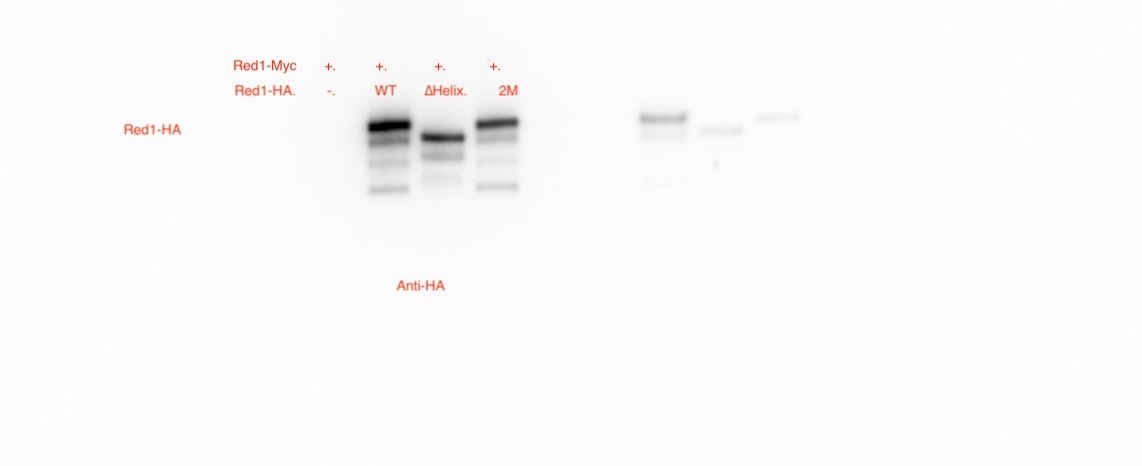

Supplement: Supplementary file 4 — Source Data [file 41467_2022_32542_MOESM4_ESM.zip › Source_data/Figure7&SupFigure13/7g/7g-HA.JPG]

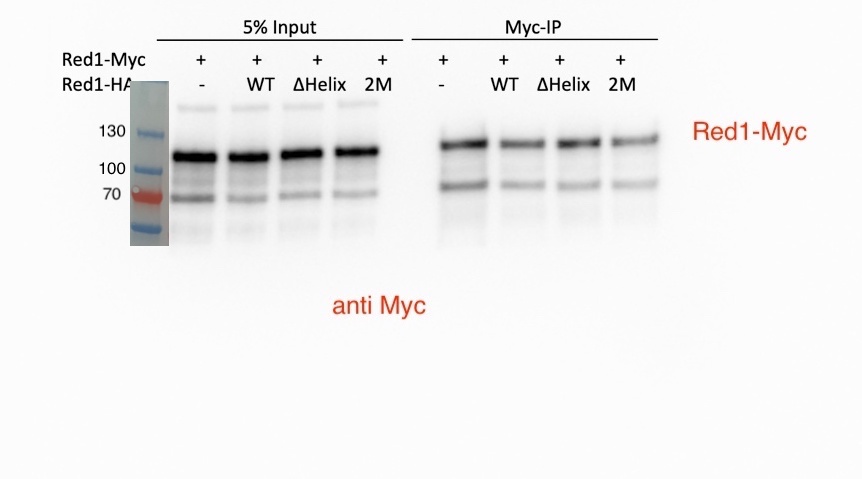

Supplement: Supplementary file 4 — Source Data [file 41467_2022_32542_MOESM4_ESM.zip › Source_data/Figure7&SupFigure13/7g/Fig7g_Myc_WithMolecularWeight.jpg]

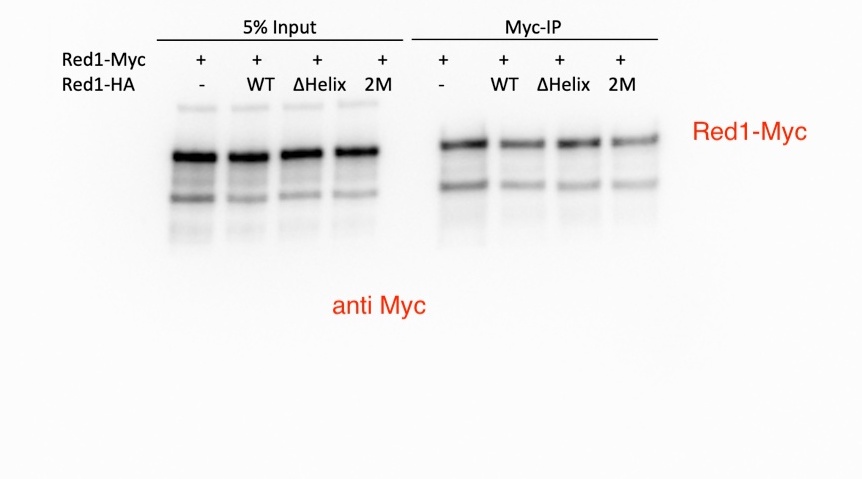

Supplement: Supplementary file 4 — Source Data [file 41467_2022_32542_MOESM4_ESM.zip › Source_data/Figure7&SupFigure13/7g/7g-Myc.JPG]

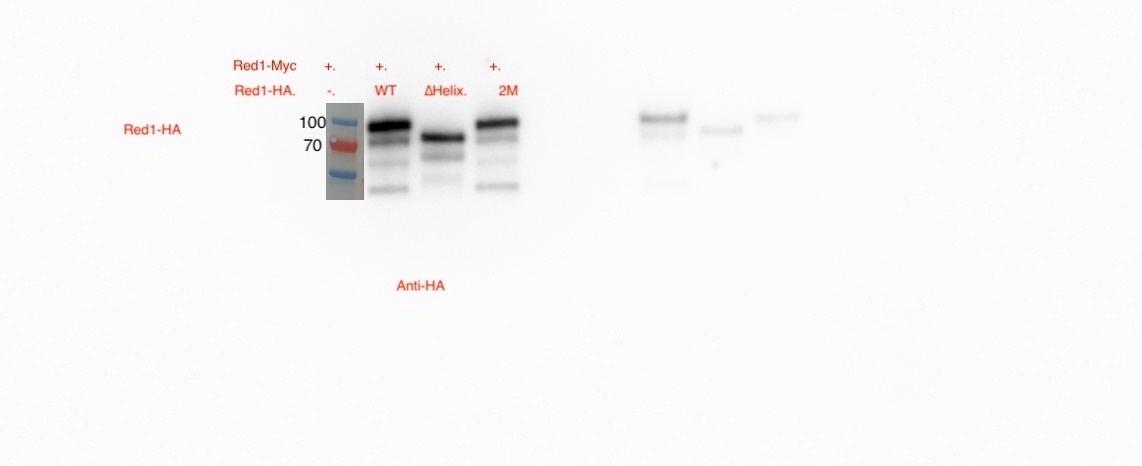

Supplement: Supplementary file 4 — Source Data [file 41467_2022_32542_MOESM4_ESM.zip › Source_data/Figure7&SupFigure13/7g/Fig7g_HA_WithMolecularWeight.jpg]
